# Interfacial Mn‐Carbon Nitride Synergy Enables Selective Photocatalytic C─H Chlorination via Single‐Atom Catalysis

**DOI:** 10.1002/anie.5772245

**Published:** 2026-09-26

**Authors:** Jaro Vanderghinste, Prakash Kumar Sahoo, Xinbang Wu, Thanh Huyen Vuong, Anna Rokicińska, Susanna Monti, Giovanni Barcaro, Alina Skorynina, Aleksander Jaworski, Vivien Michaelis, Jabor Rabeah, Julia Bornhorst, Laura Simonelli, Piotr Kuśtrowski, Adam Slabon, Paul J. Dyson, Shoubhik Das

**Affiliations:** ^1^ Department of Chemistry University of Antwerp Antwerp Belgium; ^2^ Institut des sciences et ingénierie chimiques Ecole polytechnique fédérale de Lausanne Lausanne Switzerland; ^3^ Magnetische Resonanz‐ und Röntgenmethoden Leibniz‐Institut für Katalyse (LIKAT) Rostock Germany; ^4^ Faculty of Chemistry Jagiellonian University Kraków Poland; ^5^ CNR‐ICCOM Institute of Chemistry of Organometallic Compounds Area della Ricerca Pisa Italy; ^6^ CNR‐IPCF Institute for Chemical and Physical Processes Area della Ricerca Pisa Italy; ^7^ ALBA Synchrotron Light Source Cerdanyola del Vallès Spain; ^8^ Department of Chemistry Stockholm University Stockholm Sweden; ^9^ Food Chemistry with Focus on Toxicology University of Wuppertal Wuppertal Germany; ^10^ State Key Laboratory of Low Carbon Catalysis and Carbon Dioxide Utilization Lanzhou Institute of Chemical Physics (LICP) Chinese Academy of Sciences Lanzhou China; ^11^ Chair of Inorganic Chemistry University of Wuppertal Wuppertal Germany; ^12^ Organic Chemistry I University of Bayreuth Bayreuth Germany

**Keywords:** chlorination, heterogeneous catalysis, manganese, photocatalysis, radical reactions, single‐atom catalysis

## Abstract

Single‐atom photocatalysts offer a structurally defined platform for merging homogeneous and heterogeneous catalysis, yet their application in selective C─H functionalisation remains limited. In this study, we report a manganese‐based single‐atom photocatalyst (Mn@f‐g‐C_3_N_4_) comprising atomically dispersed Mn─N_x_ sites anchored on an arylamino‐functionalised graphitic carbon nitride support. Band‐gap engineering was used to enhance visible‐light absorption to afford a robust and recyclable photocatalyst for selective visible‐light‐driven C(sp^2^)─H chlorination of arenes and heteroarenes under mild conditions. Mechanistic investigations support a dual‐site pathway initiated at the carbon nitride support and continued at the Mn centre. Photoexcitation of the carbon nitride support triggers single‐electron transfer to the N‐chlorosuccinimide chlorination reagent, generating reactive Cl species, leading to the formation of well‐defined Mn─Cl intermediates. Chlorine transfer to the arene substrate is mediated via a cyclohexadienyl radical intermediate, followed by oxidation and deprotonation, affording the chlorinated product. Cooperation between the Mn atom and support suppresses unselective free‐radical chlorination pathways and enforces surface‐confined selectivity.

## Graphical abstract

A manganese single‐atom photocatalyst on functionalised carbon nitride enables selective visible‐light‐driven C(sp^2^)─H chlorination. DFT‐supported mechanistic studies indicate that photoinduced charge separation activates NCS, while isolated Mn sites stabilize Mn─Cl intermediates at the catalyst interface. This Mn‐support synergy channels radical chlorination, suppresses overchlorination and enables efficient catalyst recycling.

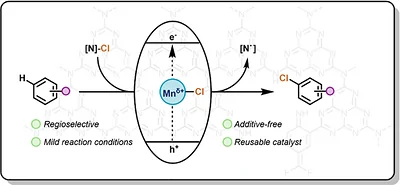

## 1 Introduction

In the pursuit of efficient chemical processes, heterogeneous catalysts are attractive due to their facile separation and recyclability [1, 2, 3]. However, conventional deposition of catalytically active metals on solid supports often leads to the formation of nanoclusters and nanoparticles, which reduces the number of active metal sites and obscures the identity of the active species [4, 5]. In this context, single‐atom catalysts (SACs) provide a compelling bridge between homogeneous and heterogeneous catalysis, combining the structural precision and mechanistic clarity of molecular systems with the robustness of solid materials, while enabling maximal metal utilisation (Figure 1a) [6, 7, 8, 9, 10, 11, 12]. When integrated with a light‐harvesting support, such systems evolve into single‐atom photocatalysts (SAPs), in which the isolated metal centres regulate light absorption, charge separation and interfacial reactivity [13, 14]. Graphitic carbon nitride (g‐C_3_N_4_) is a particularly attractive support material due to its capacity for visible‐light absorption, its chemical stability and electronically tunable framework [15, 16, 17, 18, 19]. Importantly, rational functionalisation of g‐C_3_N_4_ not only modulates its band structure and enhances its photophysical properties but also introduces well‐defined coordination motifs that effectively stabilise atomically dispersed metal sites and suppress aggregation [20, 21, 22, 23, 24]. Despite their rapid progress in energy‐ and environment‐related photocatalysis, the application of SAPs in organic synthesis remains underdeveloped, largely because synthetic organic chemistry has traditionally relied on homogeneous catalysts with predictable, well‐defined reactivity [25]. However, by reducing metal ensembles to atomically precise sites on engineered carbon nitride supports, SAPs provide molecular‐level uniformity at heterogeneous interfaces, thereby lowering the conceptual barrier for their application in selective organic transformations.

**FIGURE 1:**
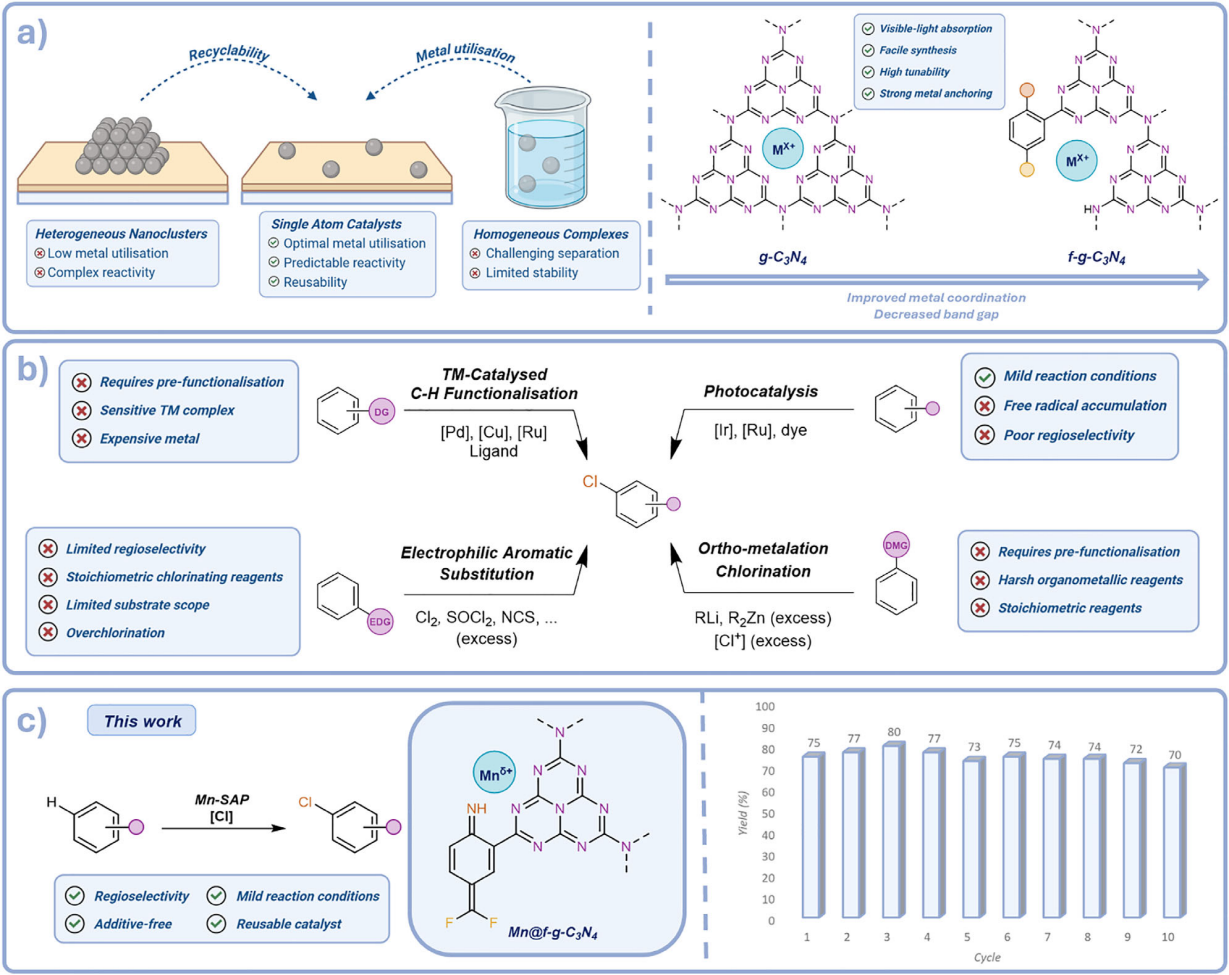
(a) Single atom catalysts as the bridge between heterogeneous and homogeneous catalysis (left) and the benefits of graphitic carbon nitride as a SAP support and the additional advantages from its functionalisation (right). (b) Current strategies for the C(sp^2^)─H chlorination of arenes. (DMG = Directed Metalation Group) (c) The strategy for the selective C(sp^2^)─H chlorination of arenes reported herein and recyclability of the catalyst.

Chlorinated arenes are privileged motifs in pharmaceuticals and agrochemicals, but also serve as versatile intermediates in cross‐coupling reactions [26, 27], nucleophilic substitution [28] and late‐stage diversification [29, 30, 31, 32]. Consequently, the direct C─H functionalisation of (hetero)arenes represents an attractive strategy to streamline synthesis by bypassing pre‐functionalised substrates, thereby reducing step count and waste [33, 34, 35, 36, 37, 38, 39, 40]. To date, C─H chlorination has been achieved primarily through electrophilic aromatic substitution [41, 42, 43], homogeneously catalysed C─H activation [44, 45, 46, 47, 48] and an *ortho* metalation/chlorination pathway (Figure 1b) [49, 50, 51]. While transition‐metal (TM) complexes based on Mn, Co, Cu, Ru and Pd and stoichiometric oxidants can enable directed or undirected C─H chlorination, these methods frequently rely on harsh conditions, limited selectivity control, or pre‐installed directing groups. In recent years, photocatalysis has emerged as a powerful platform for organic transformations under mild conditions [52, 53, 54]. In particular, visible‐light photocatalysts, including TM complexes and organic dyes, have been shown to activate N─Cl reagents or other chloride sources via single‐electron transfer (SET), generating chlorine radicals or electrophilic chlorine equivalents [55, 56, 57, 58, 59]. For example, König and co‐workers demonstrated visible‐light chlorination using flavin‐ or Ru‐based photocatalytic systems, although these protocols required sacrificial reagents or stoichiometric oxidants [55, 56]. Catalyst‐free photochemical approaches have also been reported, but typically rely on large excesses of HCl, stoichiometric additives such as NaNO_2_ or oxone, or pre‐installed functional groups that predetermine the chlorination site [60, 61]. More recently, heterogeneous photocatalysts have been reported for selective chlorination, enabling more sustainable and recyclable protocols [62, 63]. For example, Savateev and co‐workers employed a potassium poly(heptazine imide) (K‐PHI) photocatalyst to oxidise chloride under visible light [64]. However, the system required strongly acidic conditions, showed limited recyclability, and could suffer from regioisomer mixtures and overchlorination. Nevertheless, developing robust systems for heterogeneous photocatalytic C(sp^2^)─H chlorination remains challenging, particularly regarding catalyst stability. Overall, reactions involving radical intermediates often lead to diminished control, resulting in overhalogenation, broad product distributions and limited functional group tolerance [55, 56, 57, 58].

Based on our previous studies, we sought to design a manganese‐based single‐atom photocatalyst to address the key limitations of photocatalytic C─H chlorination, including poor regioselectivity arising from freely diffusing chlorine radicals in conventional photoredox catalysis (Figure 1c) [62]. Manganese, the third most abundant transition metal, was selected due to its established ability to form reactive yet controllable Mn─Cl species, which are ideally suited for selective chlorine transfer when confined to atomically defined coordination environments [44, 45, 62, 65, 66, 67, 68, 69, 70, 71]. Accordingly, we envisioned atomically dispersed Mn─N_x_ sites anchored on an arylamino‐functionalised graphitic carbon nitride framework, which should simultaneously enhance visible‐light absorption via band‐gap modulation and stabilise isolated Mn centres against aggregation, thereby ensuring a robust and structurally well‐defined catalytic platform. We reasoned that upon visible‐light irradiation, the carbon nitride support would function as the primary photoactive unit and engage in SET to N‐chlorosuccinimide, generating a chlorine‐containing reactive species that would be selectively channeled to Mn sites to form transient Mn─Cl intermediates. These species should direct chlorine transfer to arenes via cyclohexadienyl radical intermediates, followed by oxidation and deprotonation, furnishing the corresponding chlorinated products. This interfacial Mn‐mediated chlorine delivery should suppress unselective free‐radical chlorination pathways and potentially overcome long‐standing challenges of overchlorination and poor positional selectivity in C─H functionalisation [55, 56, 57, 58].

## 2 Results and Discussion

Atomically dispersed Mn on aryl amino‐substituted g‐C_3_N_4_ (Mn@f‐g‐C_3_N_4_) was synthesised through a thermal condensation of dicyandiamide (DCDA), 2‐amino‐5‐(trifluoromethyl)benzonitrile and Mn(OAc)_3_·2H_2_O, followed by pyrolysis at 550°C. Finally, the photocatalyst was ground into a fine powder before use and the Mn‐loading was quantitatively determined via ICP‐MS to be 0.81 µg/mg.

The optical and photovoltaic properties of Mn@f‐g‐C_3_N_4_ were investigated using UV–vis diffuse reflectance spectroscopy (DRS). A Tauc plot derived from the UV–vis DRS data was constructed to reveal a band‐gap in the visible region (2.60 eV), confirming that the incorporation of the arylamino moiety induces a red shift in the absorption maximum of the carbon nitride structure. Additionally, Mott–Schottky measurements indicated n‐type behaviour with a flat‐band potential of −0.964 V (vs SCE), allowing the valence (VB) and conduction band (CB) energies to be estimated at +1.44 V (vs SCE) and −1.16 V (vs SCE), respectively. These values indicate that activation of NCS via single‐electron reduction (−0.27 V vs. SCE), thereby generating a succinimidyl radical and chloride [59, 72], should be feasible with the Mn@f‐g‐C_3_N_4_ photocatalyst [73].

The Mn@f‐g‐C_3_N_4_ photocatalyst was evaluated in the C(sp^2^)─H chlorination of arenes and heterocycles. Using 1,3‐dimethoxybenzene (**1a**) was used as a model substrate with 1 equivalent of N‐chlorosuccinimide (NCS) as the chlorinating reagent in MeCN. The corresponding chlorinated product, 1‐chloro‐2,4‐dimethoxybenzene (**3a**), was obtained in 79% yield within 4 h (entry 1, Table S1). Increasing the NCS loading afforded 58% of the desired product, along with 36% of dichlorination (entry 2, Table S1). It should be noted that substituting NCS with the stronger “Palau'chlor” reagent resulted in a lower yield and poorer selectivity (entry 3, Table S1) [35]. Chloride‐containing salts such as LiCl and NaCl did not lead to any chlorinated product (entries 4–5, Table S1) and changing the solvent from MeCN to ethyl acetate, 1,2‐dichloroethane (DCE) or tetrahydrofuran (THF) did not improve the reaction (entries 6–8, Table S1). For comparison, several other TM‐based SAPs based on Pd and Fe, were investigated and related Cu and Mn catalysts previously developed by our group were evaluated, but none of these systems matched the performance of the optimised catalyst, giving lower product yields (entries 9–12, Table S1) [22, 62]. As a control, the bare graphitic carbon nitride support was also examined under the reaction conditions, however, the product was obtained in only 52% yield (entry 13, Table S1). This background reactivity can be attributed to direct photoredox activation of NCS by the photoexcited carbon nitride support [55]. Notably, incorporation of the isolated Mn sites markedly enhances the reaction efficiency, increasing the yield from 52% to 79%. This pronounced improvement highlights the beneficial role of Mn in opening a more productive chlorine‐transfer pathway involving the proposed Mn─Cl intermediates. The Mn single atoms therefore act as key catalytic sites that complement the intrinsic photoredox activity of the carbon nitride support and substantially improve productive chlorination. The optimised reaction conditions were applied on a 10 mmol scale, affording 1.73 g of isolated product **3c** (79%). Moreover, the stability of the Mn@f‐g‐C_3_N_4_ photocatalyst was confirmed by performing 10 consecutive cycles with minimal loss of reactivity (Figure 1c).

The Mn@f‐g‐C_3_N_4_ photocatalyst was evaluated against a structurally diverse set of arene substrates (Figure 2). Electron‐rich arenes are intrinsically prone to unselective electrophilic aromatic substitution, typically driven by the generation of reactive halogenating species [55, 56, 57, 58]. Advantageously, Mn@f‐g‐C_3_N_4_ overcomes this problem, suppressing uncontrolled pathways and enforcing a site‐selective mechanism. Consistent with this hypothesis, a broad range of electron‐rich substrates (**3a–3i**) undergo efficient and selective chlorination, affording the desired products in excellent yields. Notably, the Mn@f‐g‐C_3_N_4_ photocatalyst exhibited pronounced activity even with aza‐heterocycles (**3j–3n**), with no evidence of overreaction under the applied conditions. This level of control was extended to structurally and electronically challenging substrates bearing a free NH group (**3o**) or sulphur‐containing heterocycle (**3p**) that can induce catalyst deactivation [74, 75, 76, 77]. These results suggest that Mn@f‐g‐C_3_N_4_ not only mediates selective chlorination but also mitigates undesired side reactions through controlled, surface‐confined reactivity.

**FIGURE 2:**
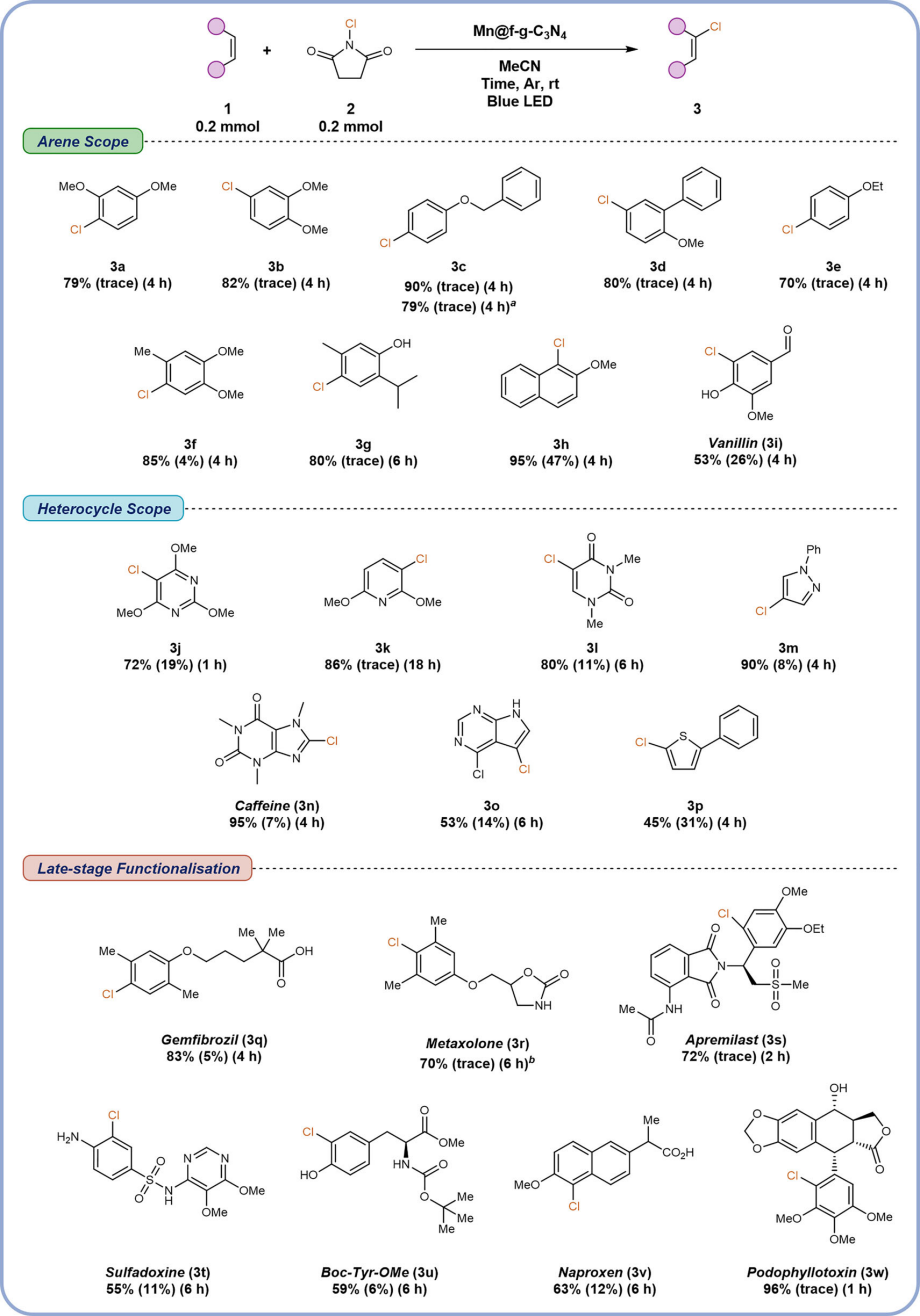
Reaction conditions: **1** (0.2 mmol), **2** (0.2 mmol), Mn@f‐g‐C_3_N_4_ (5 mg), MeCN (0.2 M), blue LED (24 W). Yields were determined by ^1^H NMR spectroscopy using dibromomethane as an internal standard. Yield of reaction in absence of catalyst in parentheses. (ab) Isolated yield from a 10 mmol scale reaction using Mn@f‐g‐C_3_N_4_ (250 mg) and MeCN (0.2 M). 1.2 equiv. **2** were used.

The mechanistic advantages of the Mn@f‐g‐C_3_N_4_ photocatalyst become even more apparent when applied to complex molecular architectures [78, 79]. Late‐stage functionalisation of pharmaceutically relevant compounds (**3q–3w**) proceed with high chemo‐ and regioselectivity, delivering moderate to excellent yields while leaving sensitive functional groups such as carboxylic acids, alcohols and amines intact. The preservation of these functionalities provides strong evidence against the involvement of highly reactive, non‐selective intermediates in solution and instead supports a mechanism in which reactive species are generated, modulated and consumed at the catalyst surface.

The structure of the Mn@f‐g‐C_3_N_4_ photocatalyst was determined using high‐resolution, high‐angle annular dark‐field scanning transmission electron microscopy (HAADF‐STEM), which confirmed the presence of isolated single manganese atoms distributed across the graphitic carbon nitride surface (Figure 3a,b). Notably, the surface of the used photocatalyst remains unchanged after reaction. Furthermore, energy‐dispersive x‐ray spectroscopy (EDX) confirmed the retention of Mn atoms after the reaction and revealed the presence of chlorine, suggesting the coordination of chlorine‐containing species to the catalyst surface.

**FIGURE 3:**
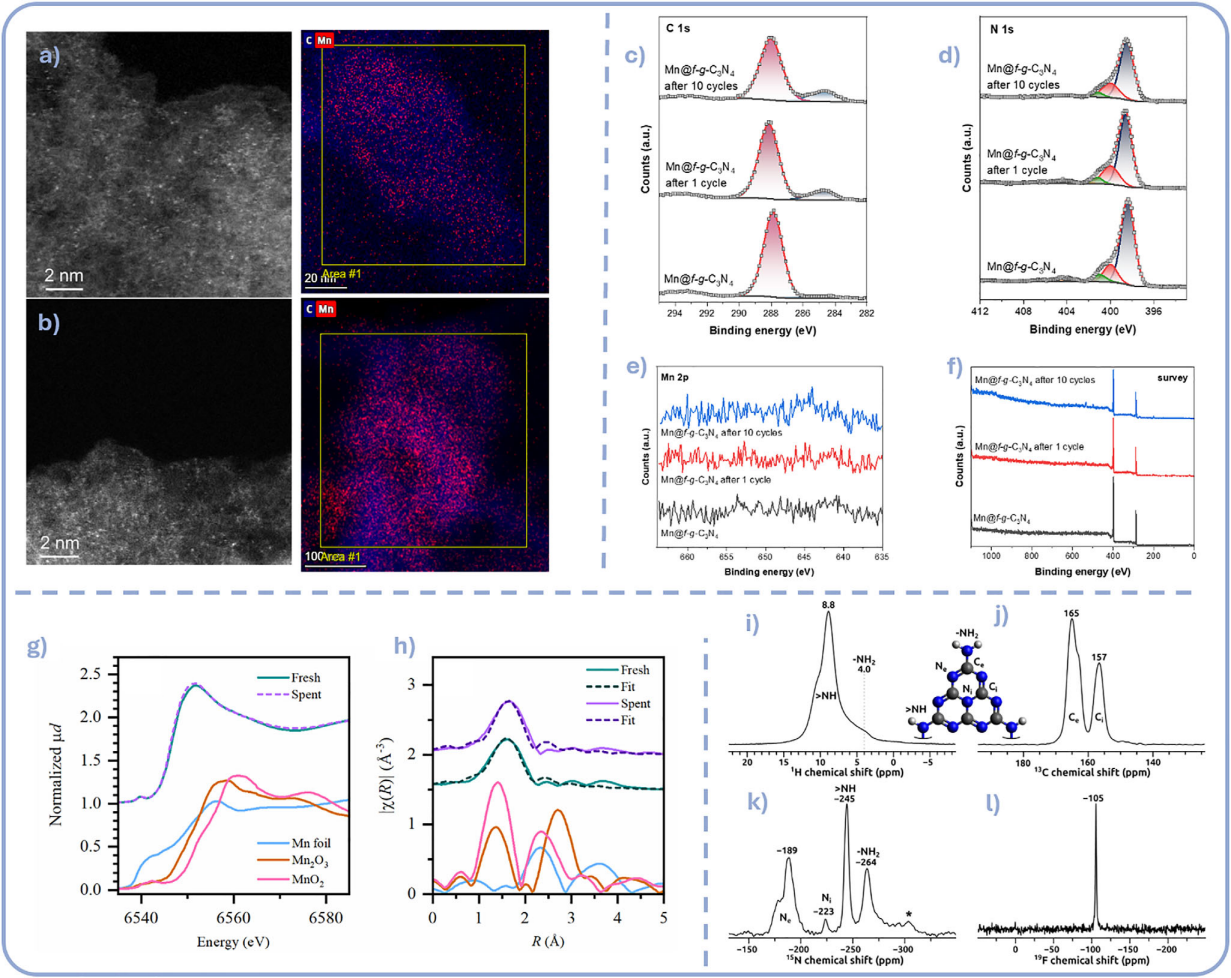
(a, b) HAADF‐STEM image and elemental EDX mapping of fresh and used Mn@f‐g‐C_3_N_4_ catalyst. (c–f) High‐resolution C 1s, N 1s, Mn 2p and survey XPS spectra of Mn@f‐g‐C_3_N_4_ before, after 1 and 10 reactions. (g, h) Normalized XANES spectra at the Mn K‐edge and Fourier transform (FT) of k^2^‐weighted EXAFS spectra with best fits. (i–l) ^1^H MAS, ^13^C CPMAS, ^15^N CPMAS and ^19^F MAS ssNMR spectra.

X‐ray photoelectron spectroscopy (XPS) was used to further investigate the surface composition of Mn@f‐g‐C_3_N_4_ and to monitor its evolution over 10 reaction cycles (Figure 3c–f). For this purpose, spectra were recorded in the C 1s, N 1s and Mn 2p regions, along with survey spectra, for the fresh photocatalyst and the photocatalyst after 1 and 10 batches. For the fresh photocatalyst, peaks are observed at 288.0 eV (sp^2^‐bonded C in N─C═N units), 398.4 eV (sp^2^‐bonded N in C─N═C), 400.0 eV (bridging N atoms in N─(C)_3_) and 401.0 eV (amino‐type N functionalities) confirming the formation of the f‐g‐C_3_N_4_ framework. Mn‐related signals are absent in the Mn 2p region, which indicates that manganese is highly dispersed on the surface [80]. After the reaction, an additional peak appears at approximately 200 eV in the survey spectrum, which is characteristic of the Cl 2p binding energy region and confirms the presence of chlorine‐containing species on the photocatalyst surface. In addition, the intensities of characteristic C 1s and N 1s peaks from the f‐g‐C_3_N_4_ structure decrease and a new carbon signal appears at 284.8 eV, which increases in intensity over time. This peak may be attributed to the formation of a carbonaceous deposit on the surface of the Mn@f‐g‐C_3_N_4_ photocatalyst. Quantitative analysis shows that the relative contribution of the C─C/C═C component associated with this carbonaceous material increases from 4% to 13% after the first run and plateaus at 17% after ten consecutive reactions.

X‐ray absorption near edge spectroscopy (XANES) was used to study the Mn@f‐g‐C_3_N_4_ photocatalyst before and after reaction (Figure 3g), together with standard reference compounds, that is Mn foil, MnO_2_ and Mn_2_O_3_. The oxidation state of the Mn‐atoms in the fresh and used Mn@f‐g‐C_3_N_4_ photocatalyst is positively charged (Mn^δ+^, 1< *δ* < 2). The Fourier transform (FT) of the *k^2^*‐weighted EXAFS oscillations (Figure 3h) indicates that Mn─Mn bonds are not present, implying the presence of Mn as isolated ions dispersed on the carbon nitride‐matrix.

Solid‐state ^1^H, ^13^C, ^15^N and ^19^F MAS NMR spectra were recorded to probe the local structure and chemical integrity of the Mn@f‐g‐C_3_N_4_ photocatalyst (Figure 3i–l). The ^1^H MAS NMR spectrum contains a main signal at 8.8 ppm arising from bridging ─NH─ groups together with a much lower intensity signal at 4.0 ppm that may be assigned to terminal ─NH_2_ groups. The ^13^C CPMAS and ^15^N CPMAS NMR spectra (Figure 3l,m) contain the characteristic resonances expected for carbon nitride, confirming the structural integrity of the material (see the structural model in the inset for signal assignments) [81]. The ^19^F MAS NMR spectrum (Figure 3n) contains a single fluorine resonance at −105 ppm which may be attributed to the presence of a = CF_2_ group and indicates, together with the ^13^C MAS NMR signals observed between 140 and 150 ppm, successful functionalisation of the g‐C_3_N_4_ with the fluorinated aryl moiety [21]. To determine the reaction mechanism, initially on–off irradiation experiments were performed and intriguingly, these experiments revealed a continuous increase in product yield even in the absence of light, suggesting a radical chain‐propagation mechanism (Figure 4a). The absence of any significant background reactivity was further confirmed by control experiments, in which only 6% of product was observed after 5 days in complete darkness. In contrast, 2 h of irradiation followed by 4 h of dark incubation results in a yield of 56%, corresponding to an additional 45% product formation during the dark period (Figure 4b). Extending the dark incubation time to 18 h did not further increase the yield, which plateaued at approximately 55%, indicating that the reaction proceeds until depletion of the reactive species generated during the irradiation phase. Notably, shortening the irradiation time to 1 h reduces the plateau to 26%, independent of the duration of the subsequent dark period. Collectively, these observations indicate the presence of radical species during the reaction, while suggesting the photoexcitation generates reactive intermediates capable of sustained, yet limited, product formation in the absence of continued light irradiation.

**FIGURE 4:**
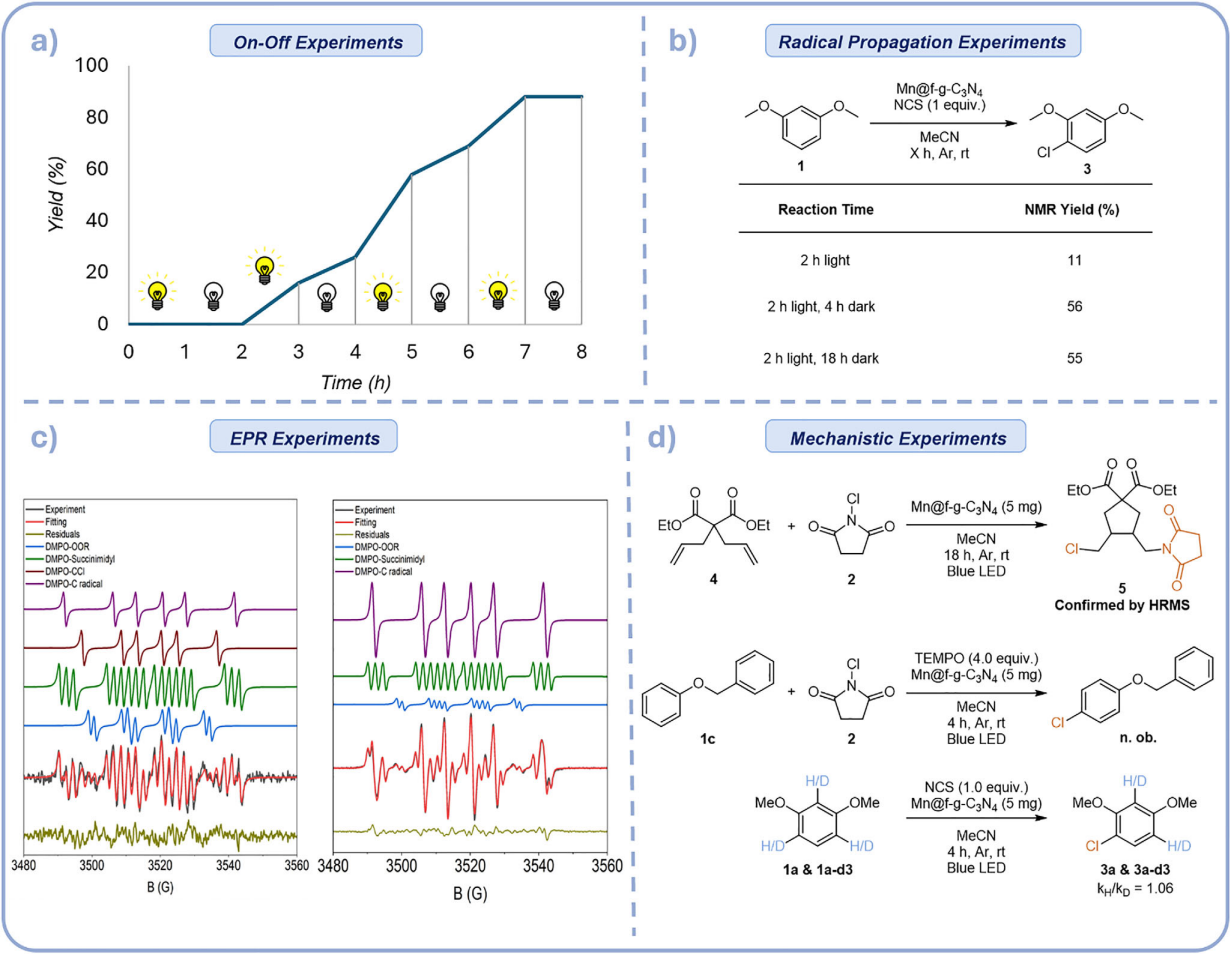
Overview mechanistic study. (a) On–off experiments. (b) Radical propagation experiments. (c) EPR spectra of reaction mixtures in the presence of DMPO recorded at room temperature after 6 h irradiation outside of the resonant cavity beforehand (left) and after 6 h irradiation outside of the resonant cavity and an additional 20 min irradiation inside the resonant cavity of the EPR spectrometer (right), together with fitting spectra and all simulated spectral components. (d) Radical probe experiment, radical quenching experiment and KIE experiment.

EPR spectroscopy of the crude model reaction mixture after 6 h light irradiation reveals four radical components (Figure 4c, **left**). The dominant species, accounting for 62% of the total signal intensity, exhibits a strong signal at *g*_iso_ = 2.007 (*A*_N_ = 14.04 G, *A*_H_^β^ = 20.53 G, *A*_N_^β^ = 2.15 G) and is assigned to the DMPO‐succinimidyl radical adduct. In addition, two carbon‐centred DMPO radical adducts are observed, contributing 11% (*A*_N_ = 11.69 G, *A*_H_^β^ = 14.50 G) and 12% (*A*_N_ = 14.55 G, *A*_H_^β^ = 21.31 G) of the total signal intensity, respectively. The comparatively low *A*_H_^β^ value of the former may indicate the presence of chlorine in the carbon‐centred radical. A fourth component, accounting for 15% of the total intensity, is assigned to a DMPO–COO^•^ radical adduct (*g*_iso_ = 2.0067 (*A*_N_ = 12.04 G, *A*_H_^β^ = 9.78 G, *A*_H_^γ^ = 1.68 G). To probe the radical population under operating conditions, the reaction mixture was subjected to an additional 20 min of in situ irradiation inside the resonant cavity of the EPR spectrometer. Under these conditions, the carbon‐centred DMPO adduct with *A*_N_ = 14.55 G and *A*_H_^β^ = 21.31 G becomes the dominant species, accounting for 53% of the total intensity. This pronounced shift in the radical distribution upon continuous irradiation indicates that the relative populations of the trapped radical intermediates strongly depend on the irradiation conditions, with formation of the carbon‐centred radical being particularly enhanced under continuous illumination.

To further substantiate the presence of radical species, a series of trapping and spectroscopic experiments were designed to probe the involvement of both succinimidyl and chlorine radicals in the reaction mechanism. Diethyl 2,2‐diallyl malonate (**4**) was used as a dual radical trap capable of intercepting two distinct radical species per molecule (Figure 4d) [26]. The formation of the mixed adduct (**5**), incorporating both a chlorine and a succinimidyl radical, was confirmed by high‐resolution mass spectrometry (HRMS), providing direct evidence that both radical species are generated under the reaction conditions and contribute to product formation. Additionally, the radical nature of the transformation was further validated by quenching experiments with 2,2,6,6‐tetramethylpiperidin‐1‐yl)oxyl (TEMPO), which completely suppresses product formation, as confirmed by GC–MS analysis. These results provided strong evidence for a radical mechanism, in which both chlorine‐ and succinimidyl‐derived radicals are essential for sustained reactivity.

A series of control experiments was conducted to probe the underlying reaction mechanism. In the absence of either the photocatalyst or light irradiation, product formation was not observed, confirming the necessity of both components for catalytic turnover. Furthermore, performing the reaction under air rather than an inert atmosphere resulted in a pronounced decrease in yield, likely due to competitive quenching of photogenerated electrons by oxygen at the CB, thereby inhibiting the reduction of NCS. In addition, the introduction of butan‐1‐amine as a coordinating ligand (catalyst poison) results in a decrease in reactivity of the accessible Mn sites.

DFT calculations were performed to probe the reaction mechanism using a representative model of the catalyst consisting of two melem units with a chelated Mn atom and the ligand (Figure 1c and in Section S5). The projected density of states (pDOS) (Figure 5a) indicates that the electronic states of the aryl moiety populate the upper region of the valence band of the Mn@f‐g‐C_3_N_4_ photocatalyst. As previously discussed, the incorporation of substituted benzonitriles into the g‐C_3_N_4_ matrix generates strong excitation bands between 400 and 450 nm. By means of time‐dependent DFT (TDDFT), a pronounced absorption band is observed at 432 nm (Figure 5b), dominated by a one‐electron excitation from the HOMO‐1 to LUMO. Upon excitation, the iso‐surface plots show that holes localise on the aryl moiety. In contrast, electrons are mainly shifted to neighbouring melem units, which dominate the lower‐energy region of the CB. Two additional features of the pDOS are noteworthy: (i) the presence of virtual states approximately 1 eV above the CB minimum (CBM), originating from the Cl atom of NCS and accessible upon photoexcitation; (ii) the presence of substrate‐derived states at the VB maximum (VBM) edge, suggesting that hole migration to the adsorbed aromatic substrate can take place. Figure 5c shows the optimised configuration (also labelled as **State 1** in the catalytic cycle of Figure 6) together with **3a** and NCS co‐adsorbed on the Mn@f‐g‐C_3_N_4_ photocatalyst in proximity to the aryl group. The substrate coordinates via the aromatic ring to the Mn centre, similarly to that observed for a related Cu@f‐g‐C_3_N_4_ [22]. The NCS interacts with the PC through weak, non‐covalent interactions.

**FIGURE 5:**
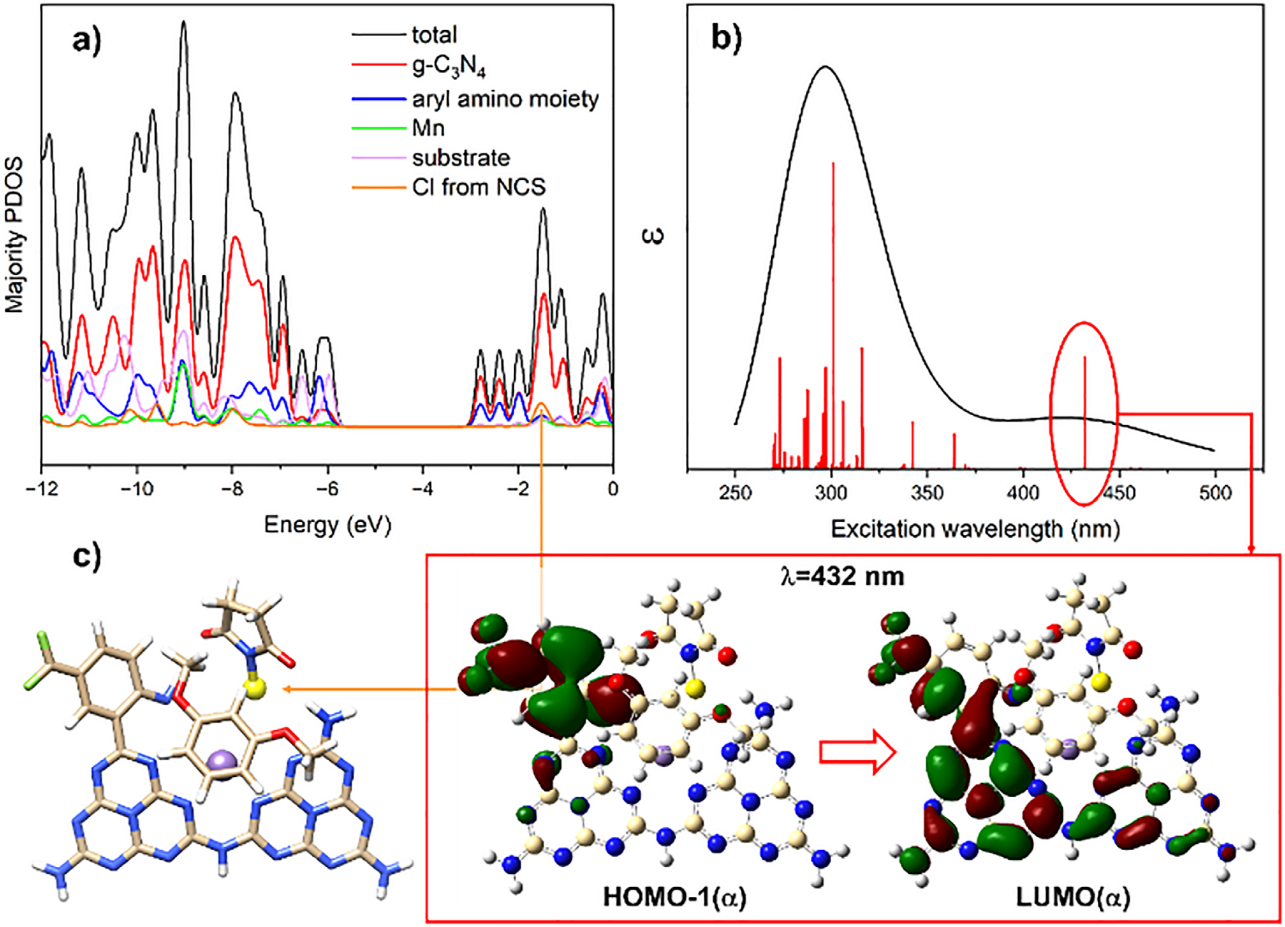
(a) Projected density of states (pDOS) and (b) TDDFT absorption spectrum of the molecular system shown in panel (c), corresponding to **State 1** of Figure 6. The orange arrow in panel (a) marks the energy region of the virtual states localised on the Cl atom of NCS. The excitation at 432 nm is dominated by the single‐particle transition from the HOMO‐1 to the LUMO orbitals, whose iso‐surfaces are displayed in the bottom‐right panel. Colour code: C (light brown), N (blue), F (green), O (red), H (white), Cl (yellow) and Mn (purple).

**FIGURE 6:**
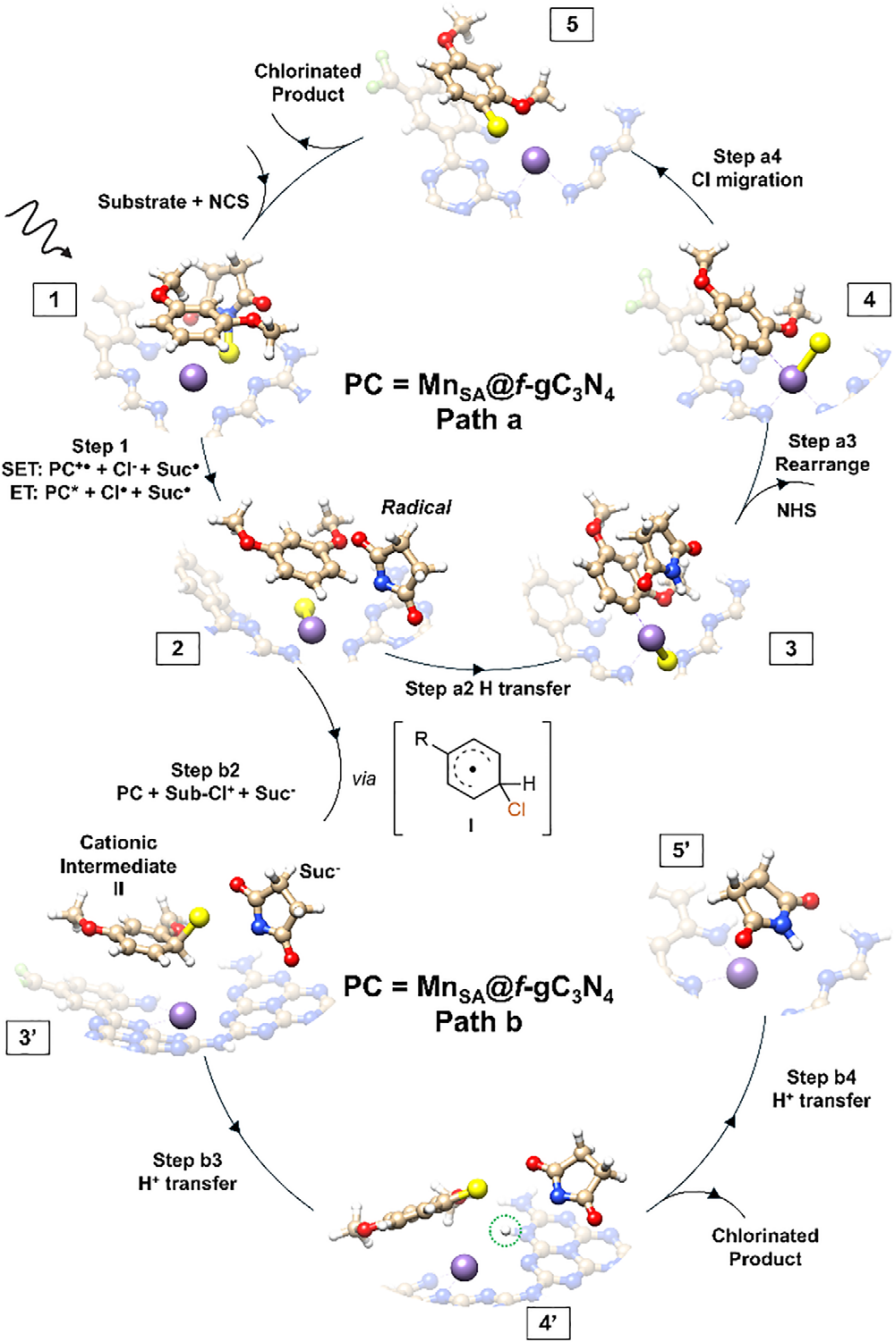
(a) Catalytic cycles showing the evolution from reactants to products according to Path a and Path b. Colour code: C (light brown), N (blue), F (green), O (red), H (white), Cl (yellow) and Mn (purple).

The reaction can proceed by following the Path depicted in Figure 6. Step 1 of the reaction consists of NCS activation, which can proceed either via SET or via energy transfer (ET) [59, 82]. Both channels can be activated when low‐lying NCS virtual states are present in the pDOS, centred on the Cl atom. In the SET pathway, an electron is transferred from the photocatalyst CBM to NCS, generating Cl^−^ and a succinimidyl radical (Suc^•^), while leaving a hole in the VBM. In this scenario, the Mn@f‐g‐C_3_N_4_ photocatalyst acts as the electron donor in an oxidative quenching cycle, whereas NCS serves as the acceptor [83]. In the ET pathway, excitation of the photocatalyst promotes homolytic cleavage of the N─Cl bond in NCS, yielding Cl^•^ and Suc^•^ radicals. In both cases, the chlorine species becomes strongly bound to the Mn centre (**State 2** in Figure 6), initiating the reactive sequence. Although both SET and ET pathways were considered feasible for NCS activation, the detection of radical intermediates by trapping experiments supports the involvement of an ET‐type process leading to chlorine‐radical formation (Figure 4d).

Upon adsorption, natural bond orbital (NBO) analysis indicates that Cl acquires a substantial negative charge (≈ −0.7e), while the Mn centre correspondingly decreases its positive charge from about +1.7 to +1.4e. In parallel, a net positive shift in the charge of the aryl moiety relative to the ground state before NCS activation is observed. These charge‐redistribution patterns are compatible with both the SET and ET scenarios. In the SET case, the negative charge on Cl reflects the electron transferred from the PC to NCS, whereas the positive charge on the aryl moiety corresponds to the hole left on the PC. In the ET case, formal charge transfer does not accompany NCS activation, therefore, the observed positive shift in the aryl group and the accumulation of negative charge in the Mn─Cl region arise from charge drift driven by the electrophilic character of the coordinated chlorine species. From this point onward, the reaction may proceed through two alternative pathways. When the Cl atom remains coordinated to the Mn centre (**Path a**, corresponding to the upper cycle in Figure 6), hydrogen atom transfer from substrate **3a** to the succinimidyl radical takes place (step a2). This process yields a stable N‐hydrosuccinimide (NHS) species and simultaneously anchors substrate **3a** to the Mn centre. The final configuration (**State 3**) is approximately 14.1 kcal mol^−1^ more stable than State 2. This H‐transfer step proceeds through a transition state with an energy barrier of about 13.2 kcal mol^−1^ (Figure S22). The next step involves migration of the Cl atom from the Mn site to the aromatic ring of the substrate, forming the final chlorinated product. The starting point of this process is a structural rearrangement around the metal centre, yielding a local minimum (**State 4** in Figure 6) that is nearly isoenergetic with **State 3**, indicating a dynamic character of the environment at the Mn site. This Cl‐transfer step (step a4 in Figure 6) is characterised by a very low energy barrier (≈ 2.3 kcal mol^−1^, Figure S22) and results in a strongly stabilised chlorinated product (**State 5**), lying about 40.1 kcal mol^−1^ below **State 4**. The final products can readily detach from the PC, restoring the system to its initial state.

Alternatively, the reaction may proceed via **Path b**, shown in the lower cycle of Figure 6, originating from **State 2**. In this pathway, chloride can be transferred to substrate **3a**, generating a short‐lived cyclohexadienyl radical intermediate **I** (Step b2 in Figure 6), which is rapidly oxidised to cationic intermediate **II**. This oxidation is enabled by the migration of photogenerated holes from the aryl moiety, a process facilitated by the presence of substrate‐derived states at the VBM edge (Figure 5a). In parallel, NBO analysis confirms that the excited electrons naturally localise on the succinimidyl radical, generating a succinimide anion (**State 3’** in Figure 6), restoring the photocatalyst to its native state. The formation of cationic intermediate **II** could explain why electron‐rich substrates are preferred, as stabilising intermediate **II** would facilitate the single‐electron oxidation of radical species **I**. Step b3 of this catalytic cycle involves H^+^ migration from cationic intermediate **II** to an edge site of the g‐C_3_N_4_ matrix, generating **State 4’**. The corresponding transition state (Figure S22) features a relatively low activation barrier of approximately 5.1 kcal mol^−1^ and this proton transfer leads directly to the formation of the chlorinated product. The catalytic cycle is then closed by a second H^+^ migration, this time from g‐C_3_N_4_ to the succinimide anion, generating NHS and restoring overall charge neutrality (**State 5’**). The transition state for this final step (Figure S22) displays an activation barrier comparable to that of the first step, again around 5.9 kcal mol^−1^.

Overall, regardless of whether the transformation follows Path a or Path b, the catalytic sequence is accompanied by a gradual drift of electronic charge from Cl back toward the aryl moiety. In agreement with our previous study [62], the catalytic cycle may be viewed as a sequence of charge‐flow events: an initial phase in which photoexcitation pumps negative charge from the PC to NCS (either via a direct SET or an indirect ET), enabling N─Cl cleavage and Cl coordination to Mn, followed by a second phase in which Cl migrates to the substrate, accompanied by a back‐flow of negative charge from the Mn─Cl region to the aryl moiety.

A competitive kinetic isotope experiment (KIE) using a mixture of non‐deuterated and deuterated 1,3‐dimethoxybenzene revealed only a very small isotope effect (*k*_H_/*k*_D_  =  1.04). This observation indicates that C─H (or C─D) bond cleavage is not involved in the rate‑determining step of the chlorination reaction. This behaviour is consistent with a late‐stage deprotonation event and supports the feasibility of the Path b mechanism. Given the lower activation barriers calculated for Path b relative to Path a, a mechanistic scenario is proposed in which Mn─Cl intermediates are stabilised, followed by chlorine transfer to the arene via a cyclohexadienyl radical intermediate. Subsequent oxidation and deprotonation steps then deliver the chlorinated products.

Importantly, this photocatalytic initiation step also provides a basis for the observed dark reaction behaviour. After the initial photoinduced generation of the reactive radical species, NCS can sustain the reaction in the absence of light by oxidising radical intermediate **I** to the corresponding cationic intermediate, while simultaneously generating a succinimidyl radical and chloride anion that propagate the radical chain. However, once the reactive radical species are depleted through termination pathways, the chain is no longer self‐sustaining and product formation ceases in the absence of light. This provides a mechanistic rationale for the experimentally observed finite dark propagation and the lower overall yield under continuous irradiation (see above).

## 3 Conclusions

We report a manganese‐based single‐atom photocatalytic system for the selective chlorination of a broad range of arenes and heteroarenes under mild conditions, overcoming limitations in regioselectivity and catalyst recyclability. The method may be applied to late‐stage functionalisation of complex bioactive molecules, delivering good to excellent yields. Mechanistic studies support a light‐initiated radical chain process, in which the photocatalyst acts primarily as an initiator rather than a continuous driver of reactivity. A combination of on–off experiments, trapping studies, EPR spectroscopy and quenching tests confirms the involvement of succinimidyl and chlorine radicals, with propagation sustained in the dark until termination events limit chain length. This work provides a mechanistic understanding of a recyclable heterogeneous photocatalytic system and highlights a promising strategy for sustainable radical chlorination chemistry.

## Author Contributions

**Jaro Vanderghinste**: investigation, writing – original draft, methodology, validation. **Prakash Kumar Sahoo**: investigation, methodology, validation. **Xinbang Wu**: investigation, methodology, validation, writing – original draft. **Thanh Huyen Vuong**: investigation, writing – original draft, methodology, validation. **Anna Rokicińska**: investigation, writing – original draft, methodology, validation. **Susanna Monti**: investigation, writing – original draft, supervision. **Giovanni Barcaro**: investigation, writing – original draft, supervision. **Alina Skorynina**: investigation, methodology, validation. **Aleksander Jaworski**: investigation, writing – original draft, methodology, validation. **Vivien Michaelis**: investigation, writing – original draft, methodology, validation. **Jabor Rabeah**: investigation, writing – original draft, methodology, validation. **Julia Bornhorst**: investigation, writing – original draft, methodology, validation. **Laura Simonelli**: investigation, writing – original draft, validation, methodology, supervision. **Piotr Kuśtrowski**: investigation, writing – original draft, supervision. **Adam Slabon**: investigation, writing – original draft, supervision. **Paul J. Dyson**: investigation, supervision, writing – review and editing. **Shoubhik Das**: conceptualization, writing – review and editing, supervision.

## Conflicts of Interest

The authors declare no conflicts of interest.

## Supporting information




**Supporting File**: anie74940‐sup‐0001‐SuppMat.pdf.

## Data Availability

The data that supports the findings of this study are available on request from the corresponding author. The data are not publicly available due to privacy or ethical restrictions.
