# Supplementary material for: Interfacial Mn‐Carbon Nitride Synergy Enables Selective Photocatalytic C─H Chlorination via Single‐Atom Catalysis
Source: Angew Chem Int Ed Engl. 2026 Sep 26;65(46):e5772245. doi: 10.1002/anie.5772245 (PMC13749365; doi:10.1002/anie.5772245)
Supplement: Supplementary file 1 [file ANIE-65-e5772245-s001.pdf]

Supporting information

Interfacial Mn-Carbon Nitride Synergy Enables Selective Photocatalytic C–H Chlorination *via* Single-Atom Catalysis

Jaro Vanderghinste,[a] Prakash Kumar Sahoo,[a] Xinbang Wu,[b] Thanh Huyen Vuong,[c] Anna Rokicińska,[d] Susanna Monti,[e] Giovanni Barcaro,[f] Alina Skorynina,[g] Aleksander Jaworski,[h] Vivien Michaelis,[i] Jabor Rabeah,[c,j] Julia Bornhorst,[i] Laura Simonelli,[g] Piotr Kuśtrowski,[d] Adam Slabon,[k] Paul J. Dyson,[b] and Shoubhik Das*[a,l]

[a] J. Vanderghinste, Dr. P. K. Sahoo, Prof. Dr. S. Das
Department of Chemistry
University of Antwerp
Groenenborgerlaan 171, 2020 Antwerp, Belgium
E-mail: Shoubhik.Das@uni-bayreuth.de

[b] Dr. X. Wu, Prof. Dr. P. J. Dyson
Institut des sciences et ingénierie chimiques
Ecole polytechnique fédérale de Lausanne
1015 Lausanne, Switzerland

[c] Dr. T. H. Vuong, Prof. Dr. J. Rabeah
Magnetische Resonanz- und Röntgenmethoden
Leibniz-Institut für Katalyse (LIKAT)
Albert-Einstein-Straße 29a, 18059 Rostock, Germany

[d] Dr. A. Rokicińska, Prof. Dr. P. Kuśtrowski
Faculty of Chemistry
Jagiellonian University
Gronostajowa 2, 30-387 Kraków, Poland

[e] Dr. S. Monti
CNR-ICCOM
Institute of Chemistry of Organometallic Compounds
Area della Ricerca, Pisa I-56124, Italy

[f] Dr. G. Barcaro
CNR-IPCF
Institute for Chemical and Physical Processes
Area della Ricerca, Pisa I-56124, Italy

[g] Dr. A. Skorynina, Dr. L. Simonelli
ALBA Synchrotron Light Source
Carrer de la Llum 2-26, 08290 Cerdanyola del Vallès, Spain

[h] Dr. A. Jaworski
Department of Chemistry
Stockholm University
Svante Arrhenius väg 16C, 114 18 Stockholm, Sweden

[i] Dr. V. Michaelis, Prof. Dr. J. Bornhorst
Food Chemistry with Focus on Toxicology
University of Wuppertal
Gaussstrasse 20, 42119 Wuppertal, Germany

[j] Prof. Dr. J. Rabeah
State Key Laboratory of Low Carbon Catalysis and Carbon Dioxide Utilization

Lanzhou Institute of Chemical Physics (LICP), Chinese Academy of Sciences
730000 Lanzhou, China

[k] Prof. Dr. A. Slabon
Chair of Inorganic Chemistry
University of Wuppertal
Gaussstrasse 20, 42119 Wuppertal, Germany

[l] Prof. Dr. S. Das
Organic Chemistry I
University of Bayreuth
Universitätstr. 30, 95447 Bayreuth, Germany

Table of Contents

1 Chemicals and materials 3

2 Experimental procedures 3

2.1 Synthesis of the Mn@f-g-C_3N_4 photocatalyst 3

2.2 Photocatalytic reaction setup 3

2.3 General procedure for the C(*sp*^{2})–H chlorination of arenes and heterocycles 4

2.4 Optimisation of the C(*sp*^{2})–H chlorination of unsaturated ring systems 4

2.5 Large-scale experiments 5

3 Mechanistic studies 6

3.1 Time course experiments 6

3.2 Recycling experiments 6

3.3 Radical probe experiments (Diethyl 2,2-diallylmalonate) 7

3.4 Radical quenching experiments 8

3.5 On-off experiment 9

3.6 Radical propagation experiments 9

3.7 Kinetic isotope effect (KIE) experiment 10

3.8 Manganese poisoning experiment 11

3.9 Electron paramagnetic resonance (EPR) spectroscopy 12

4 Analysis and characterisation 14

4.1 Chromatography 14

4.2 Inductively coupled plasma mass spectrometry (ICP-MS) 14

4.3 High-resolution mass spectrometry (HRMS) 14

4.4 UV-Vis DRS spectroscopy 14

4.5 Mott-Schottky measurements 16

4.6 Solid-state nuclear magnetic resonance (ssNMR) spectroscopy 17

4.7 X-ray photoelectron spectroscopy (XPS) 17

4.8 High-angle annular dark-field scanning transmission electron microscopy (HAADF-STEM) and energy-dispersive X-ray (EDX) spectroscopy ... 18
4.9 X-ray absorption spectroscopy (XAS) ... 19
4.10 Nuclear magnetic resonance spectroscopy ... 19
5 Computational modeling ... 25
6 References ... 27
7 Spectra ... 28

1 Chemicals and materials

Chemicals and solvents were purchased from certified chemical vendors (Acros, Sigma Aldrich, TCI, Thermo Fischer Scientific, J&K Scientific, Fluorochem and BLDpharm) and were used without prior purification. Demineralised water was obtained through deionization of tap water using a EUROTEC L4 reverse osmosis installation. This instrument filters out salts by means of a semi-permeable membrane. Water that was used never exceeded a conductivity of 0.5 µS/cm. Argon was purchased in high-pressured cylinders (200 bar) and converted to the desired pressure by means of a pressure regulator.

2 Experimental procedures

2.1 Synthesis of the Mn@f-g-C_3N_4 photocatalyst

Dicyandiamide (9 g, 107 mmol), $Mn(OAc)_3 \cdot 2H_2O$ (21.4 mg, 0.08 mmol) and 2-amino-5-(trifluoromethyl)benzonitrile (0.150 g, 0.8 mmol) were stirred in distilled water (45 mL) at 95 °C for 1 h in a closed system. The reaction vessel was then opened and heated at 100 °C until a completely dried solid was obtained. The solid was ground to a fine powder and heated at a rate of 2.2 °C/min to 550 °C, after which the temperature was kept constant for 4 h. After that, the furnace was left to cool to room temperature and the resulting solid, termed Mn@f-g-C_3N_4 was ground in an agate mortar.

2.2 Photocatalytic reaction setup

The inner surface of a round glass dish with a diameter of 140 mm was fixed with a blue LED strip with a total power of 24 W. A switch controls the intensity of illumination. An electric fan was placed over the glass dish to avoid heating by the LED irradiation. Inside the glass dish, a maximum of four vials were placed. The reaction mixture was stirred using a magnetic stirring plate and stirring bar at 400 rpm (rotations per minute) on average.

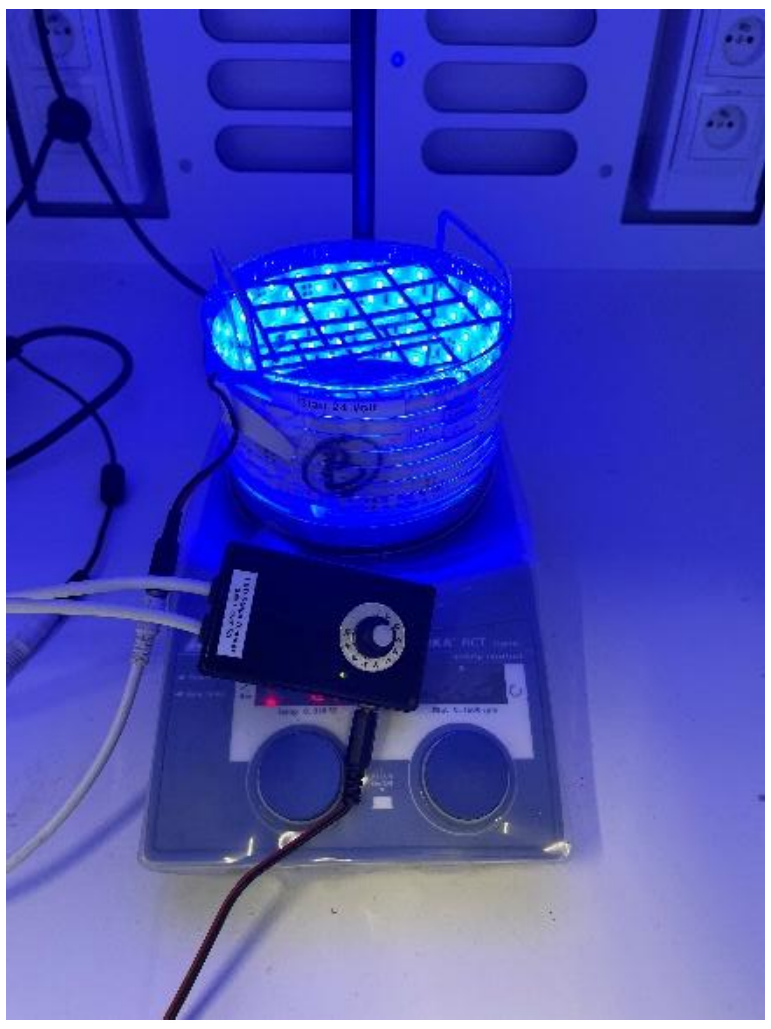

Figure S1: Photograph of the blue LED reaction setup.

The emission spectrum of the light setup was measured with a UV-Vis probe from Ocean optics (P200-5-UV-Vis). For the blue LED setup, the emission spectrum contained a wavelength band between 404 and 553 nm with a maximum at 456 nm (Figure S2).

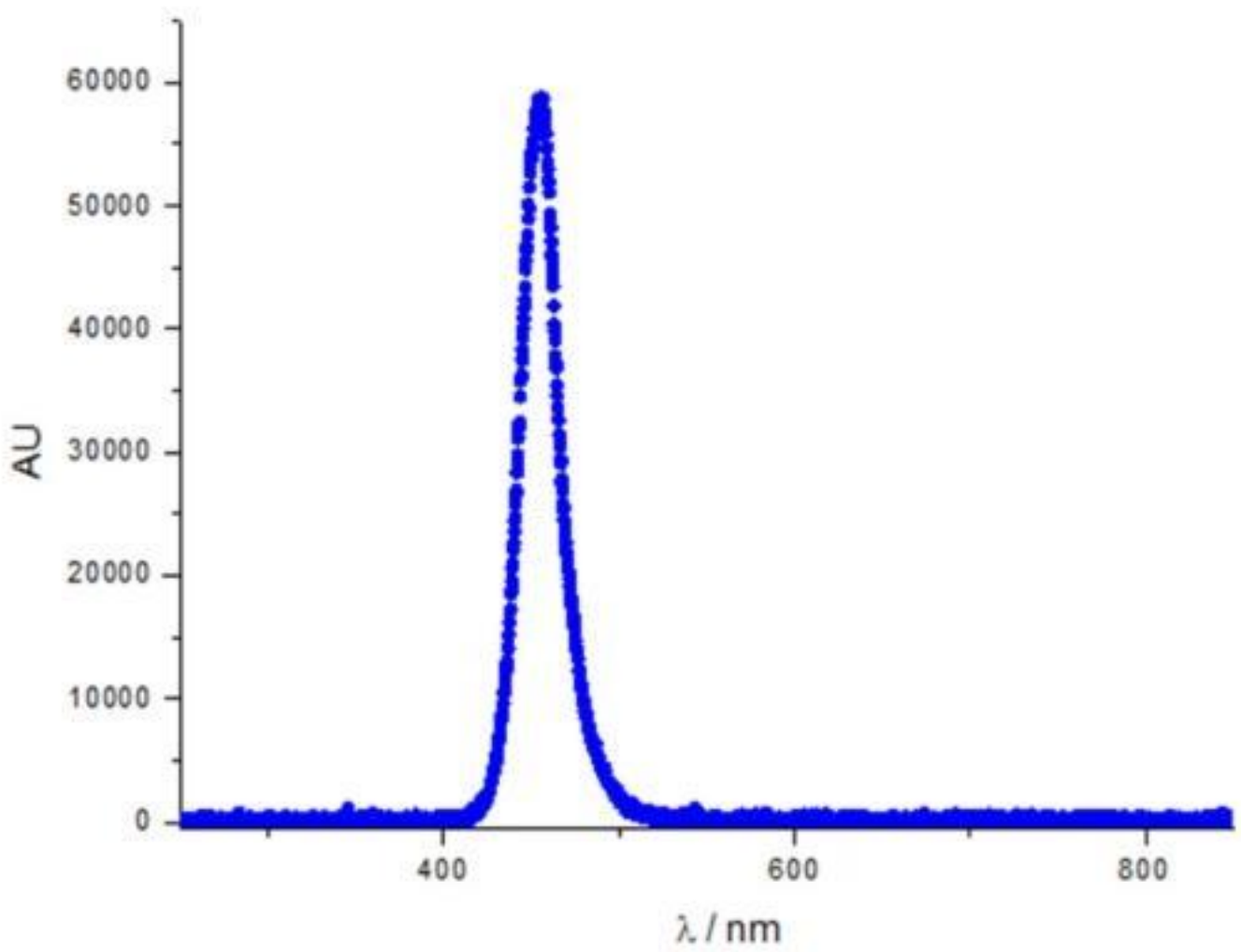

Figure S2: Emission spectrum of the blue LED light.

2.3 General procedure for the C(sp^2)–H chlorination of arenes and heterocycles

A reaction vial with magnetic stirring bar was charged with the appropriate substrate (0.2 mmol, 1.0 equiv.), NCS **2** (26.7 mg, 0.2 mmol, 1.0 equiv.), photocatalyst (5 mg), and acetonitrile (1 mL, 0.2 M) after which the vessel was closed and purged with argon for 3 minutes. The resulting mixture was stirred under irradiation of blue LED light (24 W) at room temperature. After the appropriate time, the reaction mixture was diluted with ethyl acetate (3 mL) and filtered over a cotton plug. The filtrate was concentrated in vacuo. Dibromomethane (7 µL, 0.1 mmol) was added as internal standard to determine the yield of the functionalised product using ^{1}H NMR spectroscopy.

2.4 Optimisation of the C(sp^2)–H chlorination of unsaturated ring systems

Table S1. Optimisation table of the chlorination of 1,3-dimethoxybenzene **1a**.

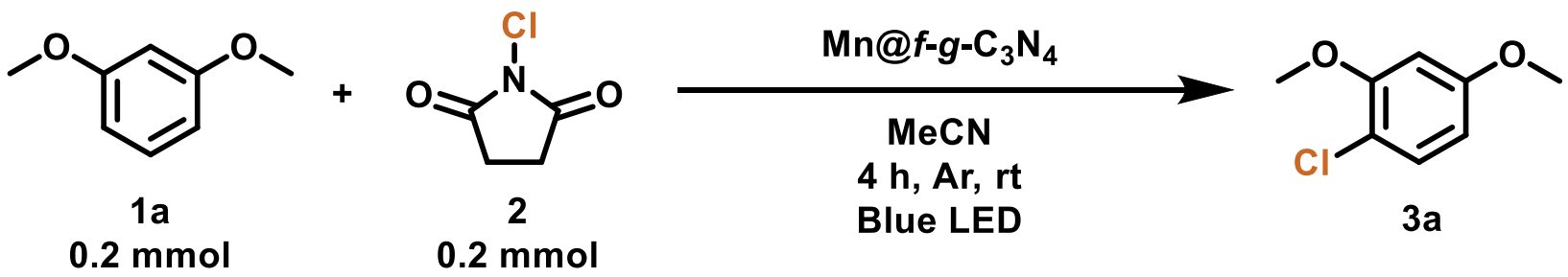

Entry[a]	Deviation	Yield (%)[b]
1	None	79
2	0.3 mmol NCS	58[c] (+ 36% dichlor.)
3	Palau'chlor instead of NCS	70 (+ 11% dichlor.)
4	LiCl instead of NCS	0
5	NaCl instead of NCS	0
6	Ethyl acetate as solvent	16
7	1,2-Dichloroethane as solvent	2
8	THF as solvent	76[c] (+ 5% dichlor.)
9	Cu@*f*-*g*-C_3N_4 from doi.org/10.1002/anie.202508512	13
10	Mn@*f*-*g*-C_3N_4 from doi.org/10.1021/jacs.4c16413	44
11	Pd@*f*-*g*-C_3N_4	59
12	Fe@*f*-*g*-C_3N_4	49
13	*f*-*g*-C_3N_4	52
14	Air	34
15	390 nm	79[c] (+ 5% dichlor.)
16	Without catalyst	0
17	Without light	0

[a] Model reaction conditions: **1a** (0.2 mmol), **2** (0.2 mmol), Mn@f-g-C_3N_4 (5 mg), MeCN (0.2 M), blue LED (24 W), 4 h, Ar, room temperature. [b] Yield determined by ^{1}H NMR spectroscopy using dibromomethane as internal standard. [c] Starting material was fully consumed

2.5 Large-scale experiments

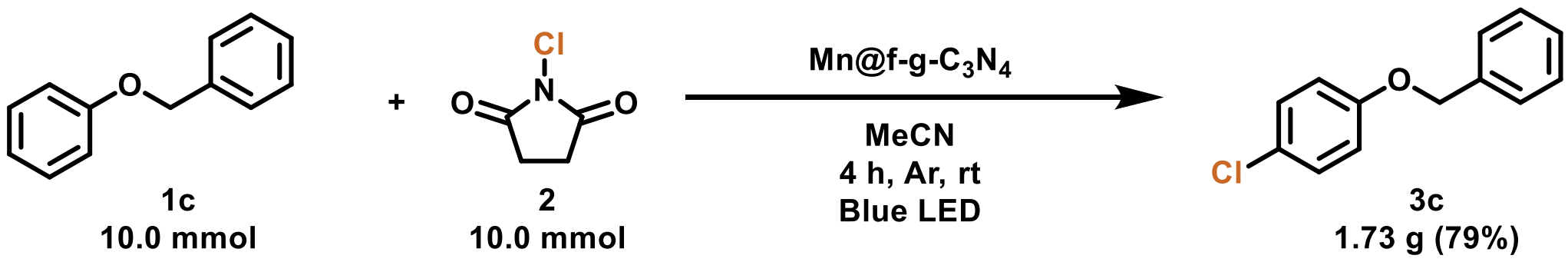

Figure S3: Reaction scheme of the radical quenching experiment.

A dried microwave vial equipped with a magnetic stirring bar was charged with benzoxybenzene **1c** (1.84 g, 10.0 mmol), NCS **2** (1.34 g, 0.2 mmol, 1.0 equiv.), and Mn@f-g-C_3N_4 (250 mg). The vessel was sealed with a septum, and acetonitrile (50 mL, 0.2 M) was added via syringe. The reaction mixture was purged with argon for 10 min and then stirred for 4 h under blue LED light irradiation at 20 °C. Upon completion, the

crude product was purified using column chromatography and the isolated product was obtained in 79% (1.73 g) yield.

3 Mechanistic studies

3.1 Time course experiments

General procedure 2.3 was used with 1,3-dimethoxybenzene **1a** (26.2 µL, 0.2 mmol) for various reaction times.

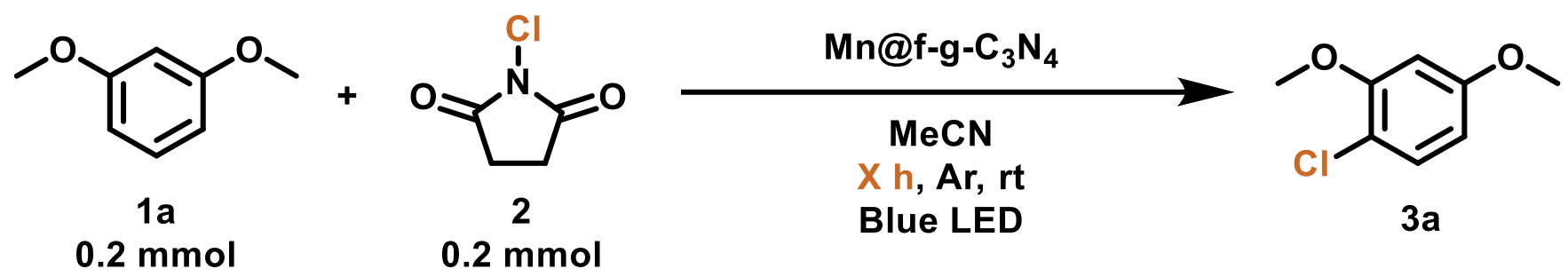

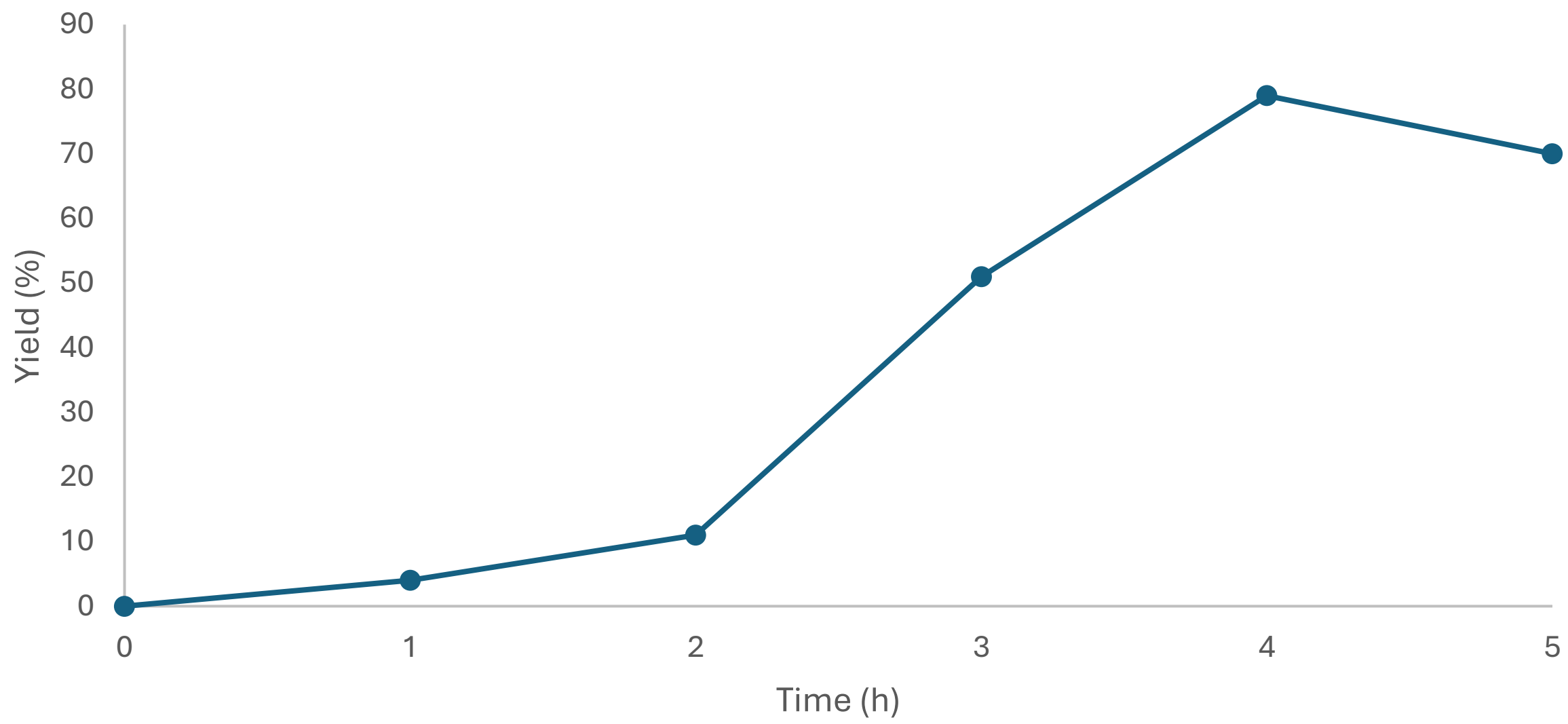

Figure S4: Product yield as a function of the reaction time.

3.2 Recycling experiments

To assess the stability of the catalyst, the C(sp^2)–H chlorination reaction of benzyl phenyl ether (**1c**) with NCS (**2**) was used in 10 consecutive experiments under identical reaction conditions with the same catalyst.

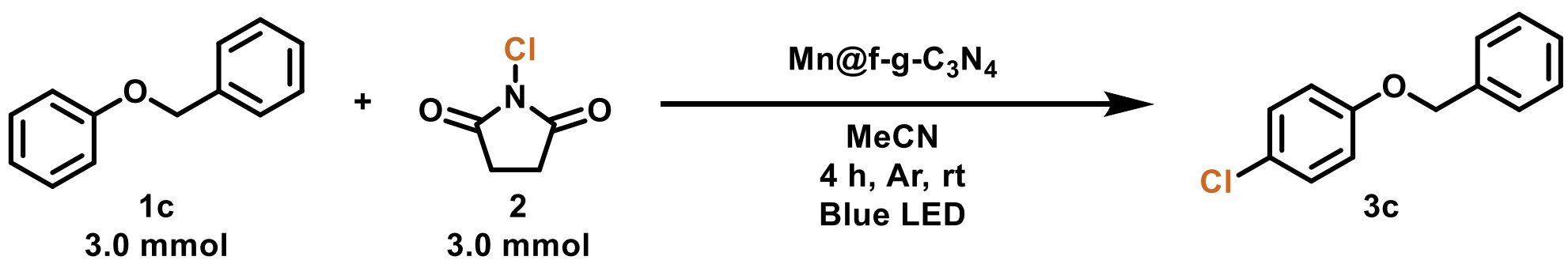

Figure S5: Reaction scheme of the recyclability experiment.

A dried round-bottom flask with magnetic stirring bar was charged with benzoxybenzene **1c** (553 mg, 3.0 mmol), NCS **2** (401 mg, 3.0 mmol, 1 equiv.), and Mn@f-g-C_3N_4 (75 mg). The vessel was closed with a septum and acetonitrile (15 mL, 0.2 M) was added using a syringe. The resulting mixture was purged with argon for

5 minutes and stirred 4 hours under irradiation of blue light at 20 °C. Then, 0.5 mL was removed from the crude mixture, and an NMR sample was prepared to check conversion. If the reaction was finished, the remaining crude mixture was centrifuged at 4000 rpm for 12 minutes. Next, the clear liquid phase was transferred to a round-bottom flask. The solid was washed with EtOAc and centrifugation was repeated 2 more times. After that, the combined liquid phases were evaporated, dibromomethane (104 μL,1.5 mmol) was added as internal standard for NMR spectroscopy. The solid phase was transferred to a round-bottom flask and dried under reduced pressure for the next cycle.

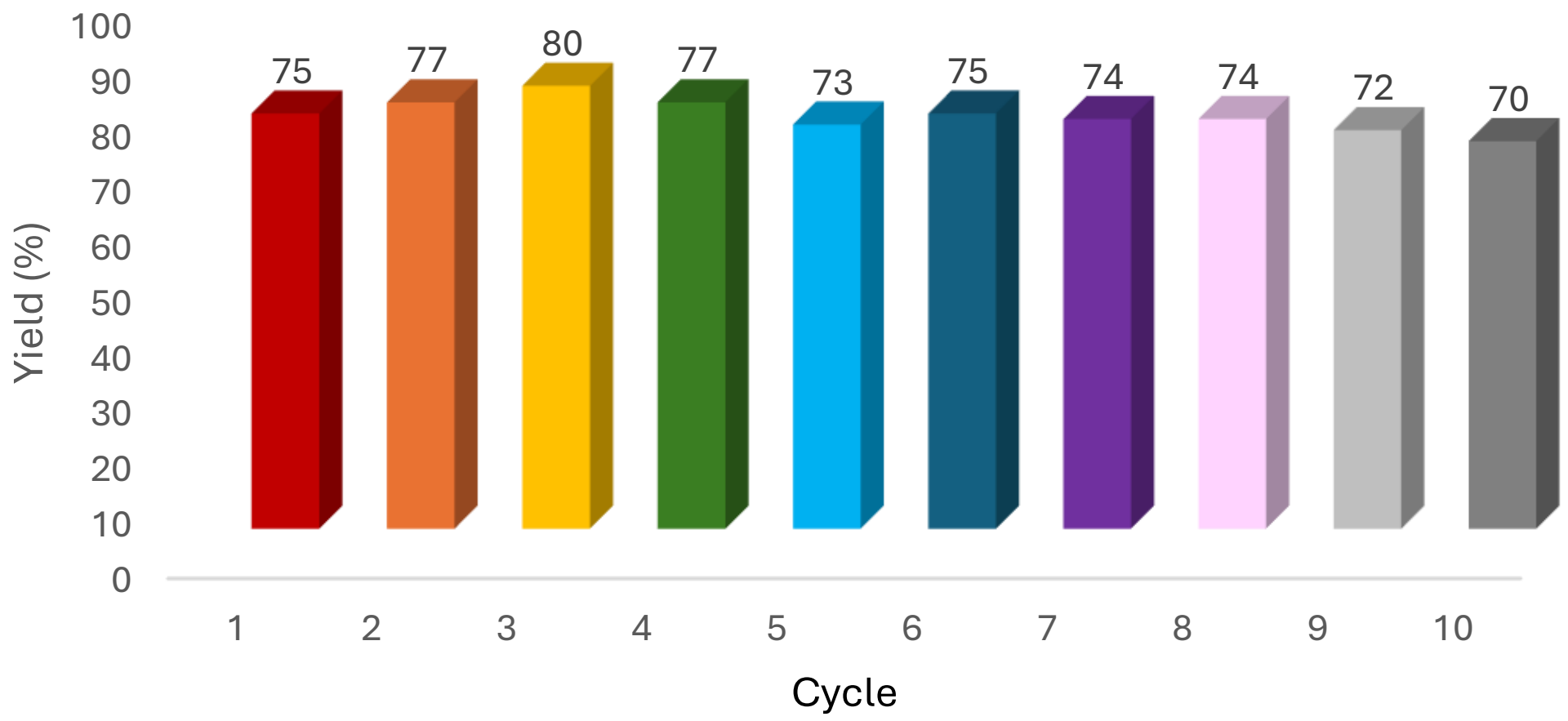

Figure S6: Yield for every reaction batch.

3.3 Radical probe experiments (Diethyl 2,2-diallylmalonate)

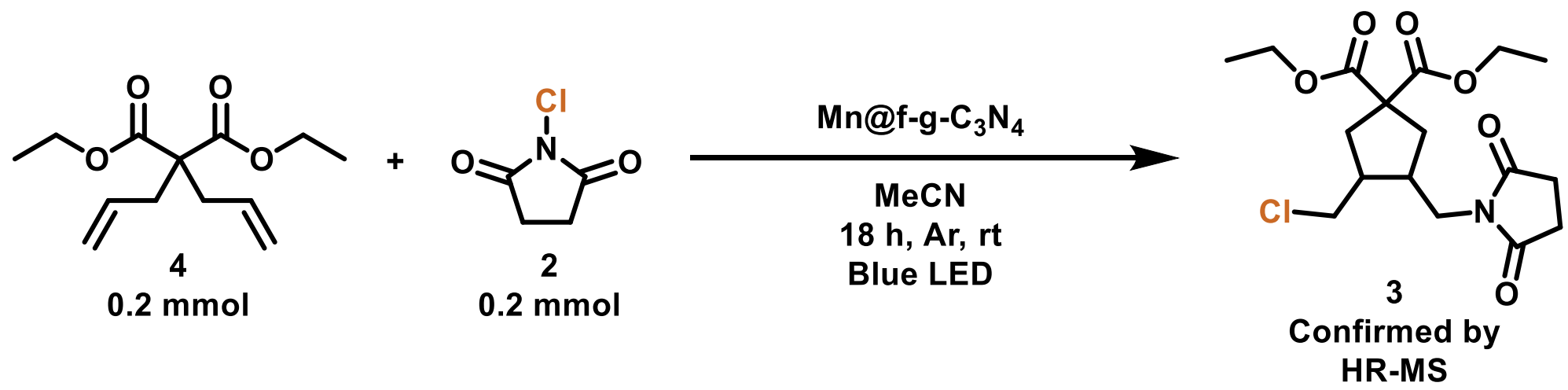

Figure S7: Reaction scheme of the radical probe experiment.

To assess the presence of radicals under the model reaction conditions, a radical probe experiment was performed using diethyl diallylmalonate. This reagent possesses two terminal alkene functionalities that are particularly susceptible to consecutive radical addition and ring closure, capturing two radical species in the process. Identification of the reaction products provides valuable information about the radical species present in the system.

A reaction vial equipped with a magnetic stirring bar was charged with diethyl 2,2-diallylmalonate **4** (48.4 μL, 0.2 mmol), NCS **2** (53.4 mg, 0.4 mmol, 2.0 equiv.), and Mn@f-g-C_3N_4 (5 mg). The vessel was sealed with a septum, and acetonitrile (1 mL, 0.2 M) was added via syringe. The reaction mixture was purged with argon

for 3 minutes and then stirred for 18 hours under blue LED light irradiation at 25 °C. The presence of the radical-added product was confirmed using HRMS. (ESI$^+$, *m/z*) [M+H$^+$] calcd. for $C_{17}H_{24}N_1O_6Cl$ 374.1365, found 374.1357.

3.4 Radical quenching experiments

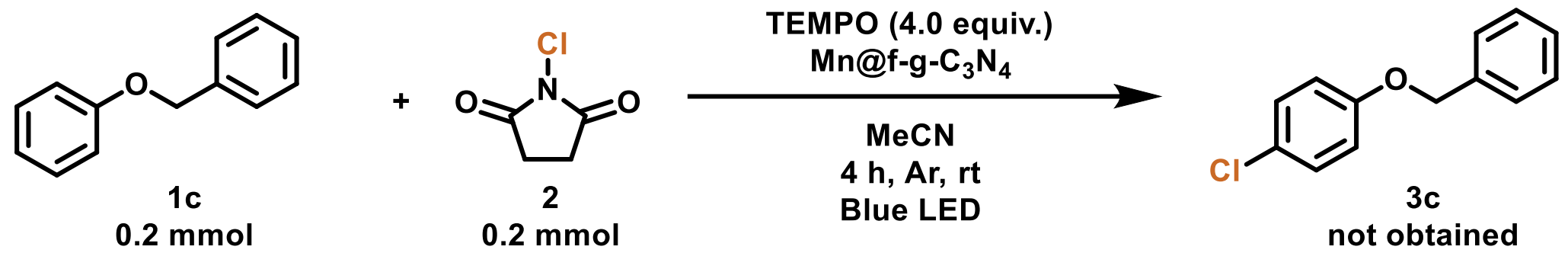

Figure S8: Reaction scheme of the radical quenching experiment.

To investigate whether chlorine radicals were generated under the reaction conditions, a radical quenching experiment was performed using 2,2,6,6-tetramethylpiperidine-1-oxyl (TEMPO). TEMPO is a stable radical capable of trapping other radical species, thereby preventing them from participating in subsequent transformations. If the reaction proceeded through a chlorine radical pathway, addition of TEMPO would suppress product formation due to radical capture. In contrast, if the transformation occurred via a chloride anion pathway, the reaction would be expected to proceed unaffected in the presence of TEMPO.

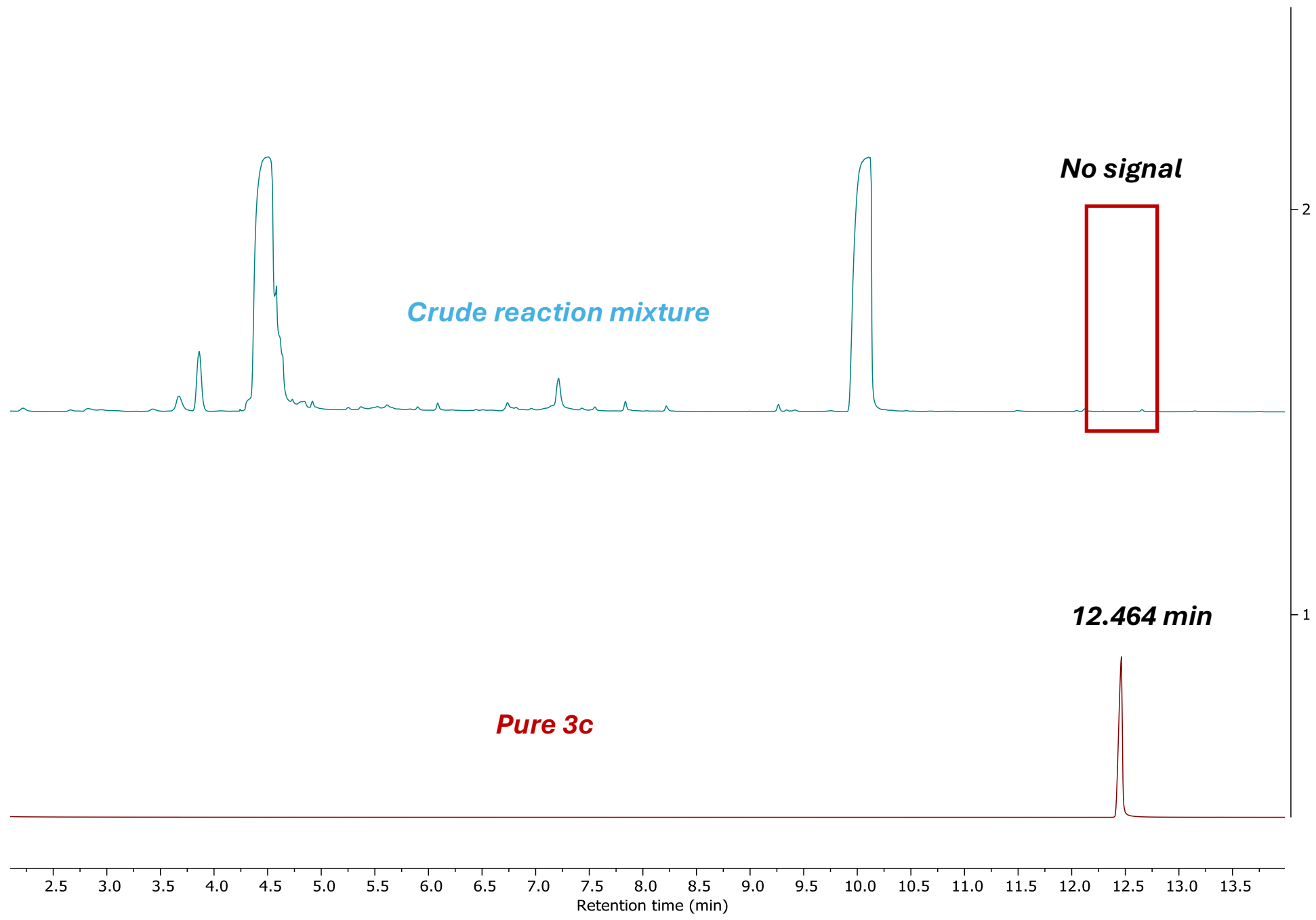

Figure S9: GC-MS chromatograms of the crude radical quenching reaction mixture (blue) and pure product (red).

A dried microwave vial equipped with a magnetic stirring bar was charged with benzoxybenzene **1c** (36.8 mg, 0.200 mmol), TEMPO (125 mg, 0.8 mmol, 4.0 equiv.), NCS **2** (26.7 mg, 0.2 mmol, 1.0 equiv.), and Mn@f-g-C_3N_4 (5 mg). The vessel was sealed with a septum, and acetonitrile (1 mL, 0.2 M) was added via syringe. The reaction mixture was purged with argon for 3 min and then stirred for 4 h under blue LED light irradiation at 20 °C. Upon completion, ethyl acetate and water were added, and the organic phase was washed 3 times with water. The combined organic extracts were filtered through a syringe filter, and the filtrate was

concentrated under reduced pressure. Dibromomethane (7 μL, 0.1 mmol) was added as an internal standard, and a sample was prepared for NMR spectroscopy to determine product yield.

3.5 On-off experiment

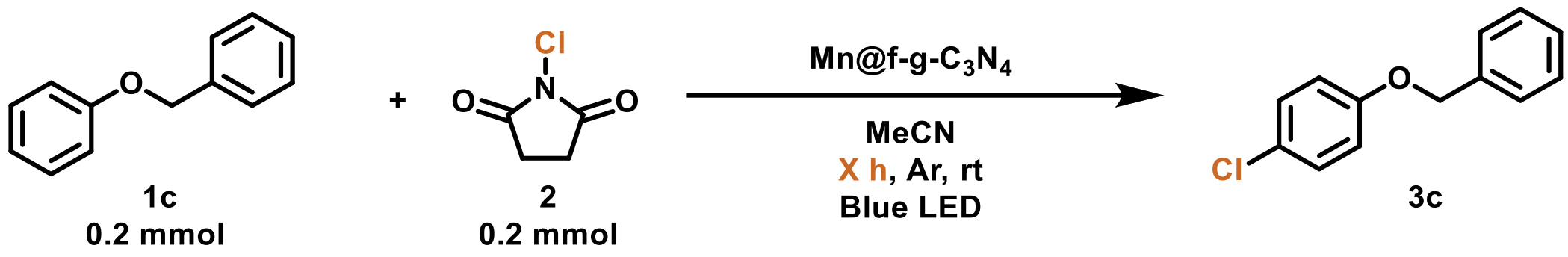

Figure S10: Reaction scheme for the on-off experiment.

A dried microwave vial with magnetic stirring bar was charged with benzoxybenzene **1c** (221 mg, 1.2 mmol), NCS **2** (160 mg, 1.2 mmol, 1 equiv.), and Mn@f-g-C_3N_4 (30.0 mg, 1.2 mmol, 1 equiv.). The vessel was closed with a septum and acetonitrile (6 mL) was added using a syringe. The resulting mixture was purged with argon for 3 minutes and stirred for 8 hours under irradiation of blue light at 20 °C. Every 60 min. the light was switched on/off and a sample of 0.5 mL was removed for analysis. Then, EtOAc was added to the sample and the mixture was filtered through a syringe filter. The filtrate was concentrated under reduced pressure, dibromomethane (7 μL, 0.10 mmol) was added as internal standard and a sample was prepared for NMR spectroscopy to determine the product yield.

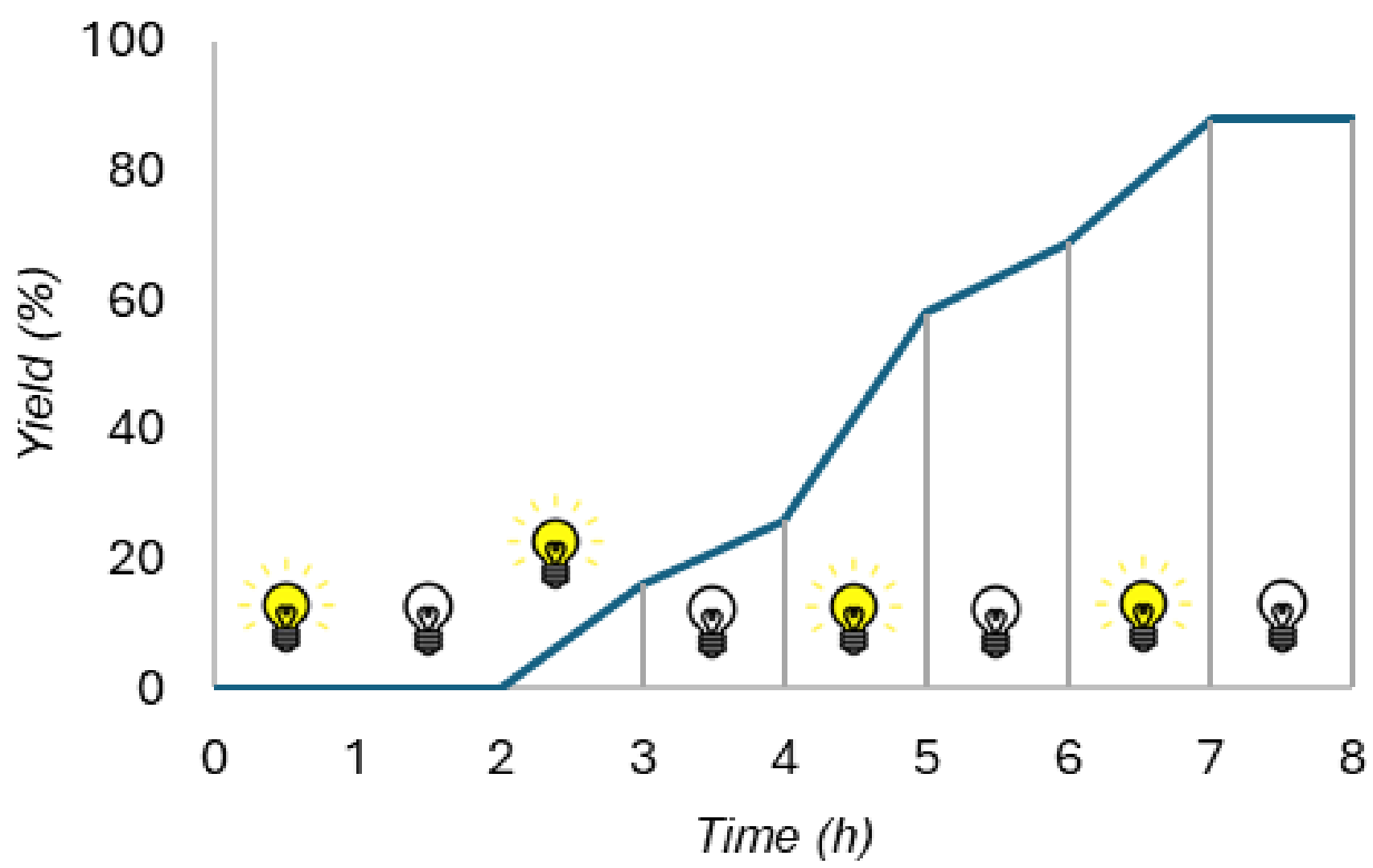

Figure S11: Yield of **3c** during the light on-off experiment, monitored at hourly intervals under alternating irradiation and dark periods.

3.6 Radical propagation experiments

A reaction vial with magnetic stirring bar was charged with the appropriate substrate (0.2 mmol, 1.0 equiv.), NCS **2** (26.7 mg, 0.2 mmol, 1.0 equiv.), photocatalyst (5 mg), and acetonitrile (0.2 M) after which the vessel was closed and purged with argon for 3 minutes. The resulting mixture was stirred under irradiation of blue LED light (24 W) at room temperature. After 0-2 hours, the light source was turned off and the reaction was left continuously stirring in the dark. After 0-18 h, the reaction mixture was diluted with ethyl acetate (3 mL)

and filtered over a cotton plug. The filtrate was concentrated in vacuo and dibromomethane (7 µL, 0.1 mmol) was added as internal standard to determine the yield of the product using ^1H NMR spectroscopy.

Table S2. Radical propagation experiments.

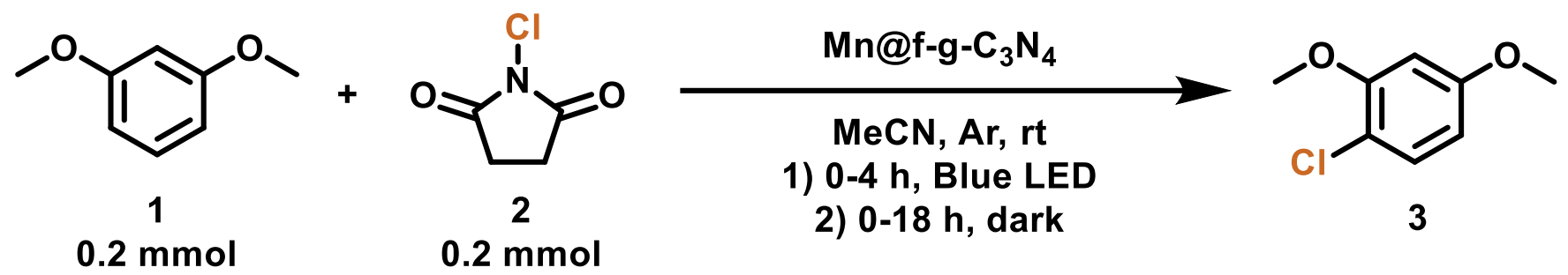

Entry[a]	Irradiation time (h)	Propagation time (h)	Yield (%)[b]
1	0	120	6
2	2	0	11
3	2	4	56
4	2	6	52
5	2	18	55
6	1	0	4
7	1	4	26
8	1	6	25

[a] Model reaction conditions: 1 (0.2 mmol), 2 (0.2 mmol), Mn@*f*-*g*-C_3N_4 (5 mg), MeCN (0.1 M), blue LED (24 W). [b] ^1H NMR yield determined using dibromomethane as internal standard.

3.7 Kinetic isotope effect (KIE) experiment

1,3-Dimethoxybenzene-2,4,6-d3 was prepared using a literature procedure.[1]

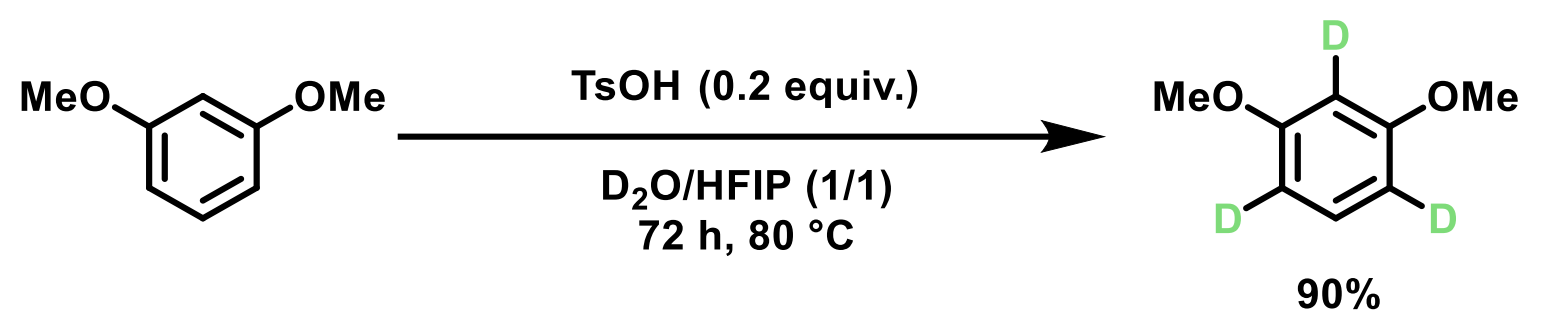

Figure S12: Synthesis of 1,3-dimethoxybenzene-2,4,6-d3.

To a dry reaction tube (10 mL) equipped with a magnetic stir bar, tosylic acid monohydrate (7.6 mg, 40 µmol, 0.2 equiv.) and 1,3-dimethoxybenzene (26.2 µL, 0.2 mmol, 1.0 equiv.) were added after which the tube was sealed. The tube was evacuated and backfilled with nitrogen three times and then D_2O (0.20 mL) and HFIP (0.20 mL) were added using a syringe. The resulting solution was heated in an oil bath at 80 °C and stirred vigorously for 72 hours. The reaction mixture was then cooled to room temperature and diluted with chloroform (5 mL) and filtered through a plug of celite, and the filtrate was concentrated under reduced pressure to obtain the products. The extent of deuterium incorporation was determined by using ^1H NMR

spectroscopy, integrated relative to unlabelled protons in the same compound and determined as 90% d2,d4,d6 incorporation.

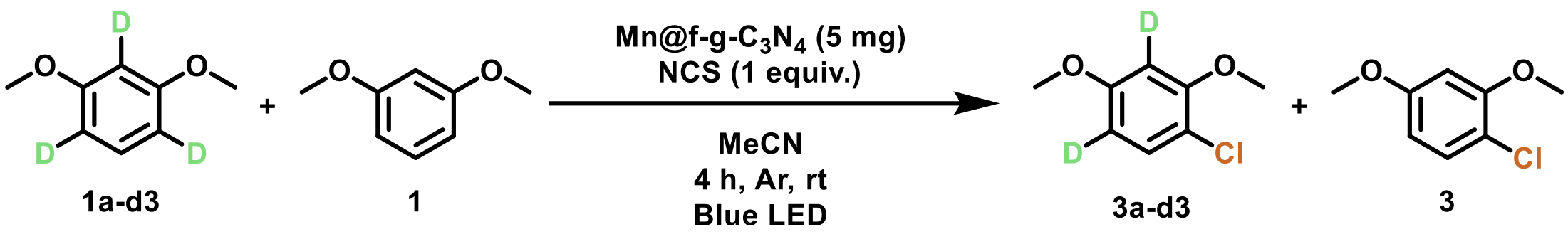

Figure S13: Reaction scheme for the KIE experiment.

A reaction vial with magnetic stirring bar was charged with the mixture as obtained in the previous step (26.2 µL, 0.2 mmol, 1.0 equiv.), NCS **2** (26.7 mg, 0.2 mmol, 1.0 equiv.), Mn@f-g-C_3N_4 (5 mg), and acetonitrile (0.2 M) after which the vessel was closed and purged with argon for 3 minutes. The resulting mixture was stirred under irradiation of blue LED light (24 W) at room temperature. After 4 hours, the reaction mixture was diluted with ethyl acetate (3 mL) and filtered over a cotton plug. The filtrate concentrated in vacuo. Dibromomethane (7 µL, 0.10 mmol) was added as internal standard to determine the yield of the product by using ^{1}H NMR spectroscopy.

The following composition was observed:

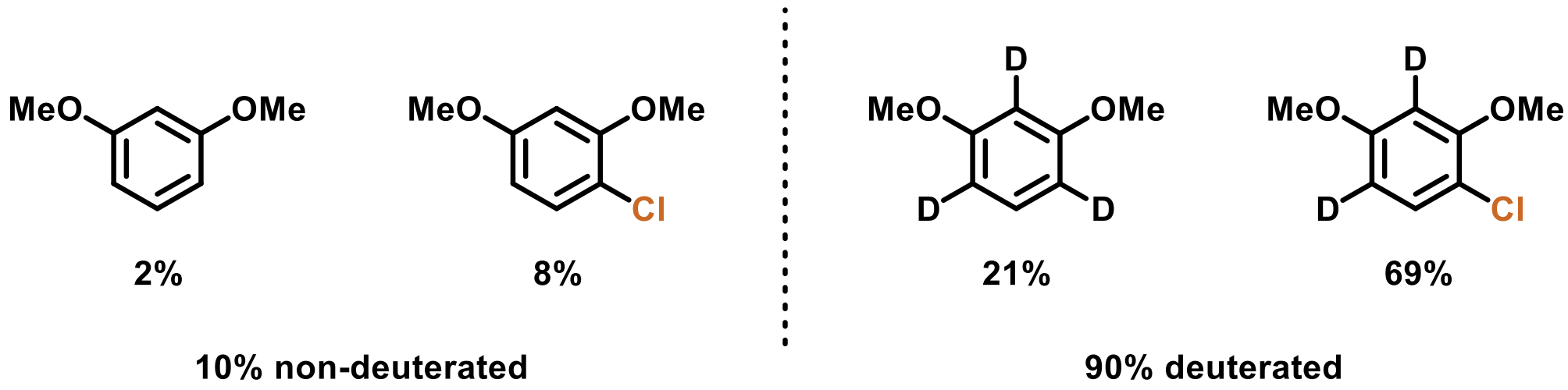

Figure S14: Results of the KIE experiment.

According to these values, the conversions for the non-deuterated and deuterated substrates are 80% (k_H) and 77% (k_D), respectively, giving a KIE value of 1.04 (k_H/k_D).

3.8 Manganese poisoning experiment

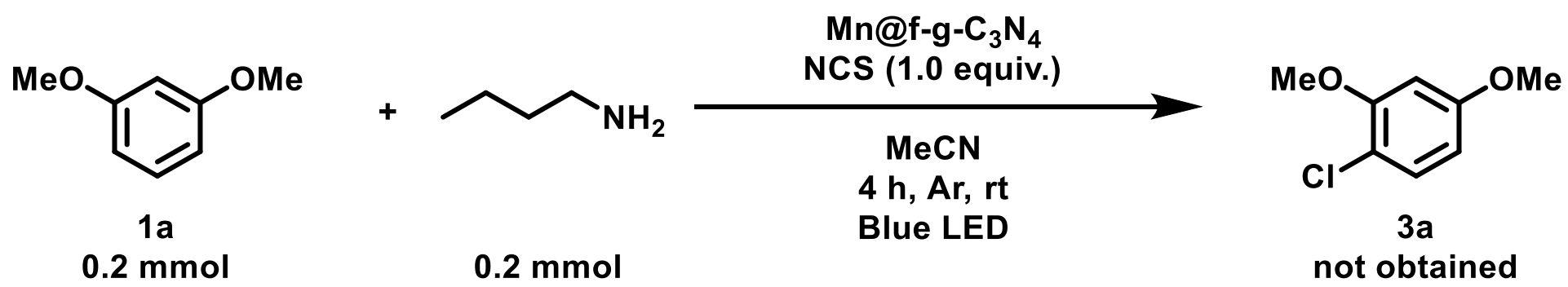

Figure S15: Reaction scheme of the manganese poisoning experiment.

A dried microwave vial equipped with a magnetic stirring bar was charged with 1,3-dimethoxybenzene **1a** (26.2 µL, 0.200 mmol), *n*-butylamine (19.8 µL, 0.2 mmol, 1 equiv.), NCS **2** (26.7 mg, 0.2 mmol, 1.0 equiv.), and Mn@*f*-*g*-C_3N_4 (5 mg). The vessel was sealed with a septum, and acetonitrile (1 mL, 0.2 M) was added via syringe. The reaction mixture was purged with argon for 3 minutes and then stirred for 4 hours under blue LED light irradiation at 20 °C. Upon completion, the reaction mixture was diluted with ethyl acetate (3

mL) and filtered over a cotton plug. The filtrate concentrated in vacuo. Dibromomethane (7 μL, 0.1 mmol) was added as internal standard to determine the yield of the product by using ^{1}H NMR spectroscopy.

3.9 Electron paramagnetic resonance (EPR) spectroscopy

A 25 mL Schlenck tube with magnetic stirring bar was charged with 1,3-dimethoxybenzene **1a** (26.2 μL, 0.4 mmol), NCS **2** (53.4 mg, 0.4 mmol, 1.0 equiv.), Mn@f-g-C_3N_4 (5 mg), and acetonitrile (0.2 M) and three freeze-pump-thaw/Ar cycles were performed. The resulting mixture was stirred under irradiation of a 456 nm Kessil lamp (40 W) at room temperature. At appropriate times, 0.1 mL of the reaction mixture was collected and mixed with 10 μL DMPO, and EPR spectra were recorded either in the absence or presence of a 456 nm light inside the resonant cavity.

EPR spectra were recorded on an X-band Bruker EMX CW-micro EPR spectrometer equipped with an ER4119HS high-sensitivity resonator using a microwave power of around 6.9 mW, modulation frequency of 100 kHz and modulation amplitude up to 5 G.

EPR spectral simulations and least squares fitting were performed using EasySpin (MATLAB). The experimental spectrum was modelled as a linear combination of four distinct DMPO spin adduct species. Fitting optimized g factors, hyperfine coupling constants, linewidth parameters and relative component weights. For multi component overlapping EPR spectra, strong parameter correlations are inherent. Therefore, model validation was based primarily on visual agreement between experimental and simulated spectra and analysis of the residual (experimental minus simulated spectrum), rather than raw RMSD values. Fitted hyperfine coupling constants were cross checked against reported literature values for DMPO derived radical adducts to confirm physical plausibility. The residual trace showing the difference between experiment and simulation is displayed alongside the main spectrum.

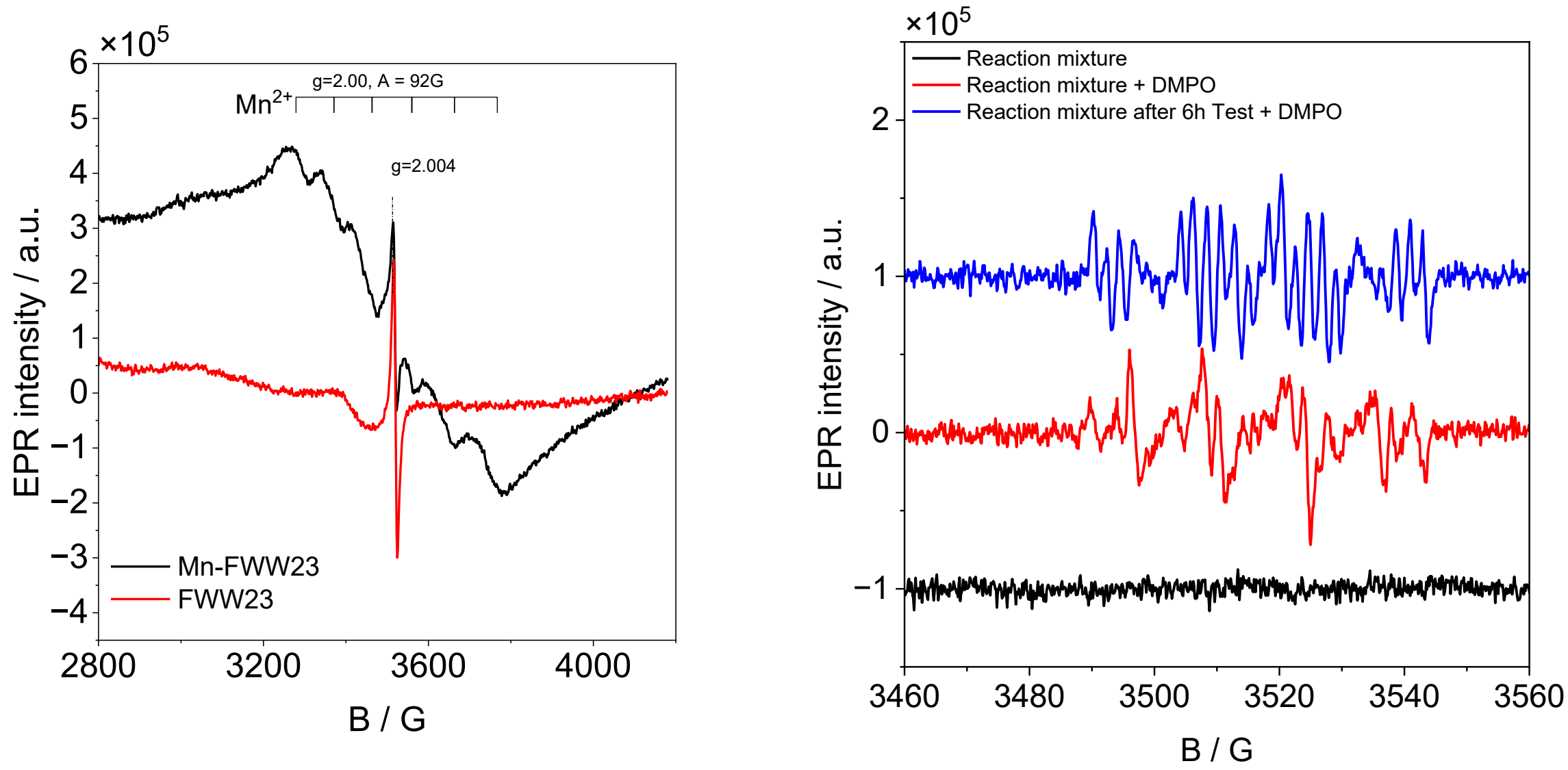

Figure S16: EPR spectra of a) Mn@f-g-C_3N_4 and bare f-g-C_3N_4 and b) the reaction mixture with (red) and without DMPO (black) as prepared and where DMPO was added after 6 h of light irradiation (blue).

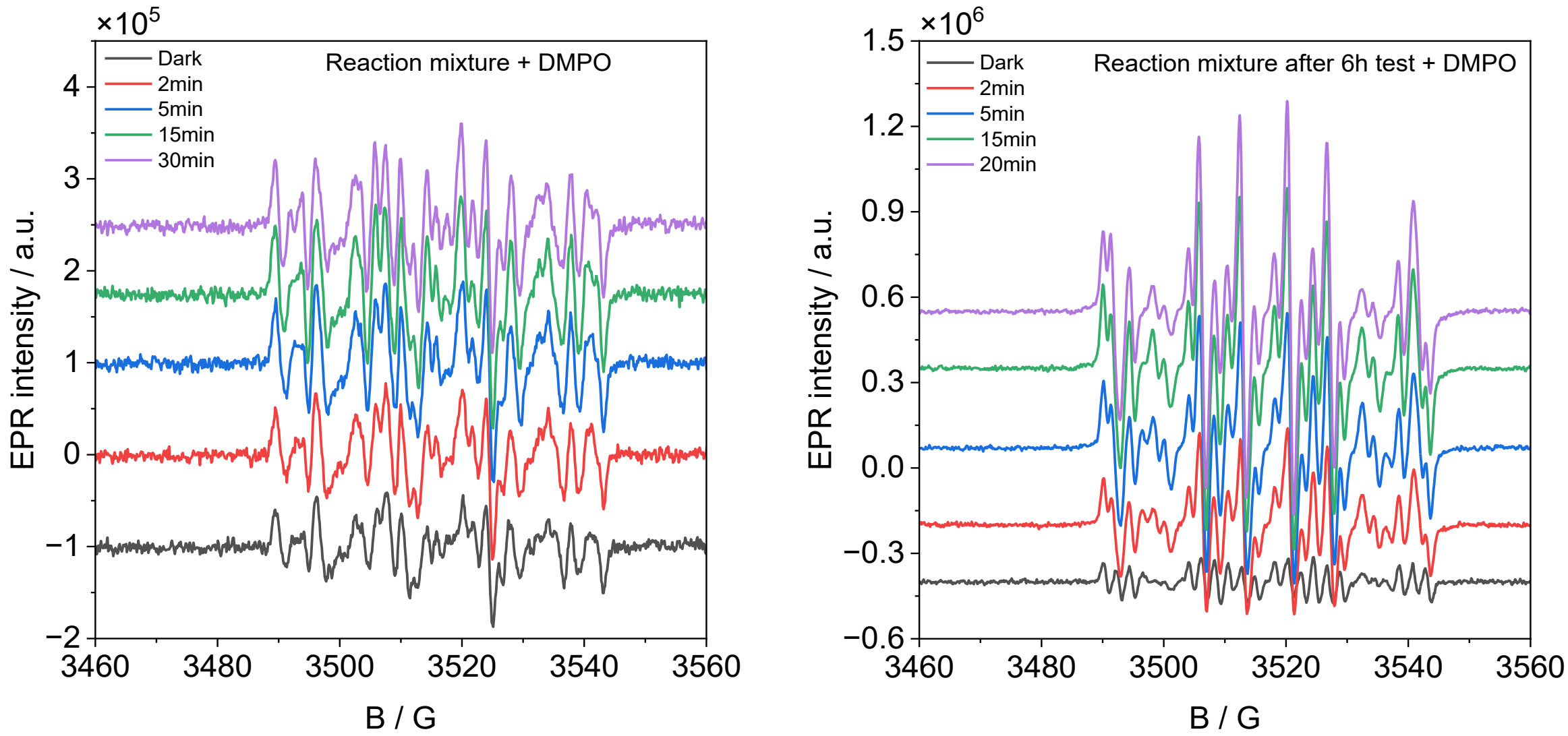

Figure S17: EPR spectra of reaction mixtures in the presence of DMPO recorded at room temperature under dark and during irradiation inside the resonant cavity of the EPR spectrometer: a) reaction mixture as prepared and b) after 6 h irradiation outside the resonant cavity beforehand.

The simulation of the EPR spectra of the reaction mixture in the presence of DMPO, shown in **Figure 4c** after 6 h of irradiation outside of the resonant cavity, exhibits 3 components. The major component, 71.5% of the total intensity, exhibits a strong signal at g_{iso} = 2.007, A_N = 14.04 G; A_H^{β} = 20.53 G; A_N^{β} = 2.15 G (green line) can be assigned to DMPO-succinimidyl$^{\bullet}$ radical adduct, while a doublet of triplets with A_N = 11.69 G, A_H^{β} = 16.19 G is related to a carbon-centred radical-DMPO adduct. The last component, 15.7% of the total intensity, a quartet signal at g_{iso} = 2.0067, A_N = 12.04 G; A_H^{β} = 9.78 G; A_H^{γ} = 1.68 G (blue line), may be assigned to the DMPO-COO$^{\bullet}$ radical adduct.

In-situ irradiation of this reaction mixture inside the resonant cavity of the EPR spectrometer results in the appearance of a different DMPO-C$^{\bullet}$ signal (strong doublet of triplets with A_N = 14.55 G, A_H^{β} = 21.31 G), which gives rise to the highest contribution of the signal at 53% of the total intensity (Fig. 4c, purple).

4 Analysis and characterisation

4.1 Chromatography

Thin-layer chromatography was performed using a petroleum ether/ethyl acetate (EtOAc) solvent system as mobile phase and a 0.20 mm silica gel on an aluminium plate (Machery-Nagel Precoated TLC sheets Alugram® SIL G/UV254) as stationary phase. Visualization of the ‘spots’ was enhanced by illumination with UV-light (254 nm).

Flash chromatography was performed using an automatic chromatographic system (Biotage® or Combiflash®) with on-line UV-detection (254 nm and 280 nm unless explicitly stated otherwise). Commercial silica cartridges (12-80 g, Grace®) were used as stationary phase.

4.2 Inductively coupled plasma mass spectrometry (ICP-MS)

The material was weighed and incubated with the ashing mixture (65% HNO_3/30% H_2O_2 (1:11) (both from Merck) at 95°C for at least 12 h. The remaining ash was diluted using 2% HNO_3 spiked with 1 µg/L Rh as internal standard to compensate drift effects. Mn was determined using an inductively coupled plasma mass spectrometer (ICP-MS; Agilent 7700, Agilent Technologies, USA) without cell gas. The parameters of lenses, collision cell and Q1 were tuned daily on a daily basis for maximum sensitivity (an oxide ratio of <0.5% ($^{140}Ce^{16}O+$/$^{140}Ce+$) and a double charged ratio of <1.5% ($^{140}Ce++$/$^{140}Ce+$). Conditions for the ICP-MS were as follows: RF Power 1550 W; Plasma gas flow 15 L/min; Nebulizer (MicroMist™) gas flow 1.05 L/min; Auxiliary gas flow 0.9 L/min; Q1 ^{55}Mn, ^{103}Rh (ISTD); 3 Replicates, 30 Sweeps/Replicate.

Determinations of blank digedtion and certified reference material (BCR-274) (Community Bureau of Reference of the Commission of the European Communities) were performed every measuring run. Data were normalized to weight.

4.3 High-resolution mass spectrometry (HRMS)

High-resolution mass spectrometry (HRMS) samples were prepared by dissolving 0.1-5.0 mg of the product in acetonitrile (or methanol) and further diluting to a concentration of 10^{-5}-10^{-6} M with 80/20 MeCN/H_2O (or MeOH)/H_2O + 0.1% formic acid. 5 µl of each sample was injected using the Acquity UPLC system (Waters, Manchester, UK) in a flow of 90/10 MeOH/H_2O 0.1% FA at 40 µl/min and electrosprayed using a standard electrospray source. Samples were injected with an interval of 2 minutes. Positive ion mode accurate mass spectra were acquired using a Synapt HDMS Q-TOF instrument (Waters, Manchester, UK). The MS was calibrated prior to use with a 0.1% H_3PO_4 solution. The spectra were lock-mass corrected using the known m/z of the nearest H_3PO_4 cluster resulting from a separate injection of a reference sample containing 0.1% phosphoric acid.

4.4 UV-Vis DRS spectroscopy

The optical properties of the heterogeneous catalyst were characterised using UV-Vis diffuse reflectance spectroscopy (UV-DRS) on a Shimadzu UV-2600i spectrophotometer equipped with an integrating sphere attachment using $BaSO_4$ as a reference standard.

Band gap energies were estimated using the Tauc plot method,[2] based on the Tauc–Davis–Mott model, which relates the optical absorption coefficient (*α*) to the photon energy (*hv*) through the equation:

$$(\alpha h\nu)^{1/n} = A\left(h\nu - E_g\right) \tag{1}$$

where *α* is the absorption coefficient, *h* is Planck's constant, *v* is the photon frequency, *A* is a proportionality constant, and E_g is the band gap energy. The exponent *n* depends on the nature of the electronic transition: n = ½ for direct allowed transitions and n = 2 for indirect allowed transitions.

Since UV-DRS measures reflectance rather than direct absorbance, the Kubelka–Munk function $F(R_\infty)$ was used to convert reflectance data to absorbance-like values:

$$F(R_\infty) = \frac{(1 - R_\infty)^2}{2R_\infty} \tag{2}$$

where $R_\infty = R_{sample}/R_{standard}$, and R_{sample} and $R_{standard}$ represent the reflectance of the sample and standard, respectively. The Kubelka–Munk function $F(R_\infty)$ is proportional to *α* and was substituted into the Tauc equation to yield:

$$[F(R_\infty)h\nu]^{1/n} = A\left(h\nu - E_g\right) \tag{3}$$

Tauc plots were generated by plotting $[F(R_\infty)h\nu]^{1/n}$ versus photon energy (*hv*), and the band gap energy was determined by extrapolating the linear portion of the plot to the photon energy axis (Figure S18). Using this method, the band gap energy of Mn@f-g-C_3N_4 was determined to be 2.60 eV.

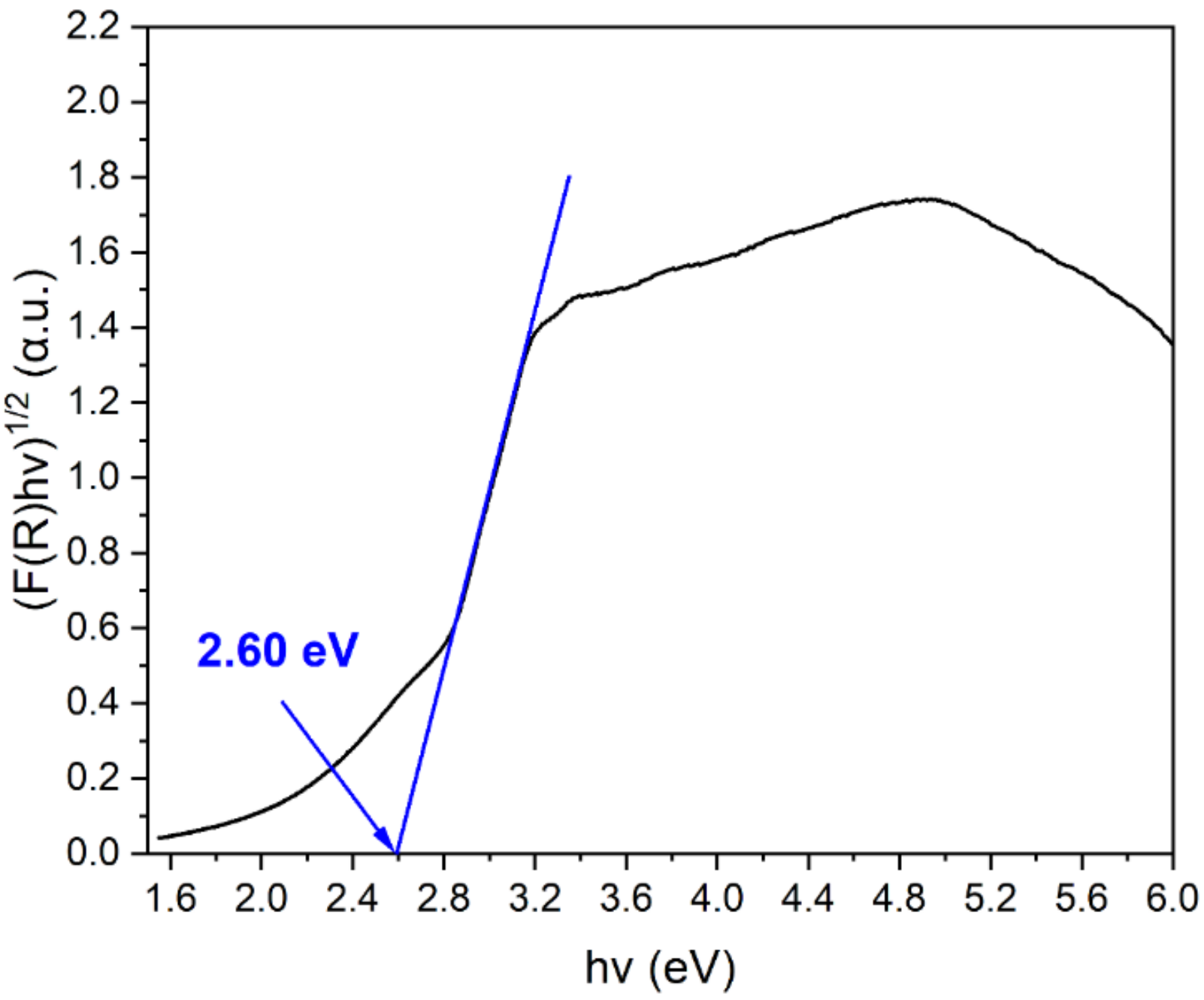

Figure S18: Band gap energy of Mn@f-g-C_3N_4 determined by extrapolation of the linear portion of the Tauc plot to the photon energy axis.

4.5 Mott-Schottky measurements

Mott-Schottky experiments were conducted using a three-electrode configuration cell with a Pt foil counter electrode and a Ag/AgCl 3 M KCl reference electrode. The working electrode consisted of a thin film, prepared by combining 5 mg of the Mn@f-g-C_3N_4 with a mixture of 50 µL ethanol and 10 µL of a 5 wt% Nafion solution, followed by sonication for 1 hour. Subsequently, 15 µL of the resulting mixture was pipetted onto a fluorine-doped tin oxide (FTO) glass surface to create 1 × 1 cm^2 coating films, which were then dried at room temperature and further dried at 40 °C in a vacuum oven overnight. The electrolyte solution employed comprised 0.5 M Na_2SO_4 (with a measured pH of 7.47), degassed with N_2. Measurements were carried out using a Metrohm Autolab potentiostat-galvanostat PGSTAT204 workstation in the absence of light, with an AC amplitude of 5 mV and frequencies of 400, 600 and 1000 Hz.

Then, to further clarify the electronic structure, electrochemical flat-band potential measurements were conducted. Typically, flat-band potential values are derived from the Mott-Schottky equation[3]:

$$\frac{1}{C^2} = \frac{2}{\varepsilon\varepsilon_0 A^2 e N_D}\left(E - E_{fb} - \frac{kT}{e}\right) \tag{4}$$

where C represents the interfacial capacitance, A the exposed area of the material, N_D the number of donors, and ε and ε_0 are the dielectric constants of the film electrode and free space, respectively. E refers to the applied potential, E_{fb} is the flat-band potential, k is Boltzmann's constant, T is the temperature, and e is the electronic charge. The flat-band potential E_{fb} can be obtained by extrapolating the plot to where $1/C^2 = 0$.

The positive slope of the Mott-Schottky curve suggests that the photocatalyst is n-type. Consequently, the flat band potential of Mn@f-g-C_3N_4 is approximately -0.964 V (vs. SCE), as determined from the intersection between the Mott-Schottky plot and the baseline. In n-type semiconductor photocatalysts, the conduction band generally lies 0.1 to 0.2 eV above the flat-band potential, with this offset influenced by factors such as electron effective mass and carrier concentration. So, the conduction band of the Mn@f-g-C_3N_4 may be calculated as -1.164 V (vs. SCE). Additionally, using the band gap energy E_g, the valence band (VB) can be determined by $E_{VB} = E_{CB} + E_g$. Therefore, the valence band of Mn@f-g-C_3N_4 was at approximately +1.436 V (vs SCE). Based on: $E_{NHE} = E_{SCE} + 0.244$ V, then $E_{CB} = -0.920$ V (vs. NHE), $E_{VB} = +1.680$ V (vs. NHE).

4.6 Solid-state nuclear magnetic resonance (ssNMR) spectroscopy

Magic-angle-spinning (MAS) NMR spectra were acquired at a magnetic field of 14.1 T (Larmor frequencies of 600.12, 150.92, 60.83, and 564.69 MHz for ^{1}H, ^{13}C, ^{15}N, and ^{19}F respectively) on a Bruker Avance-III spectrometer. ^{1}H and ^{19}F MAS NMR spectra were acquired using a 1.3 mm probehead and a 60 kHz MAS rate. Acquisition involved a use of a rotor-synchronized, double-adiabatic spin-echo sequence with a 90° excitation pulse of 1.25 μs (2.30 μs for ^{19}F) followed by a pair of 50.0 μs tanh/tan short high-power adiabatic pulses (SHAPs) with 5 MHz frequency sweep. All pulses operated at the nutation frequency of 200 kHz (110 kHz for ^{19}F). 128 signal transients (10240 for ^{19}F) were acquired using a relaxation delay of 5 s. Cross-polarization (CP) ^{1}H-^{13}C and ^{1}H-^{15}N CPMAS NMR spectra were recorded using a 7 mm probehead with a 7 kHz MAS rate and 65 kHz spinal64 proton decoupling. For ^{1}H-^{13}C CPMAS acquisition Hartmann-Hahn matched radiofrequency fields were applied for a contact interval of 1.5 ms and 2048 signal transients were collected using a relaxation delay of 5 s. The ^{1}H-^{15}N CPMAS acquisitions involved contact interval of 5ms, and 16384 scans collected with relaxation time of 5 s. Chemical shifts are reported with respect to tetramethylsilane (^{1}H, ^{13}C), nitromethane (^{15}N), and trichlorofluoromethane (^{19}F).

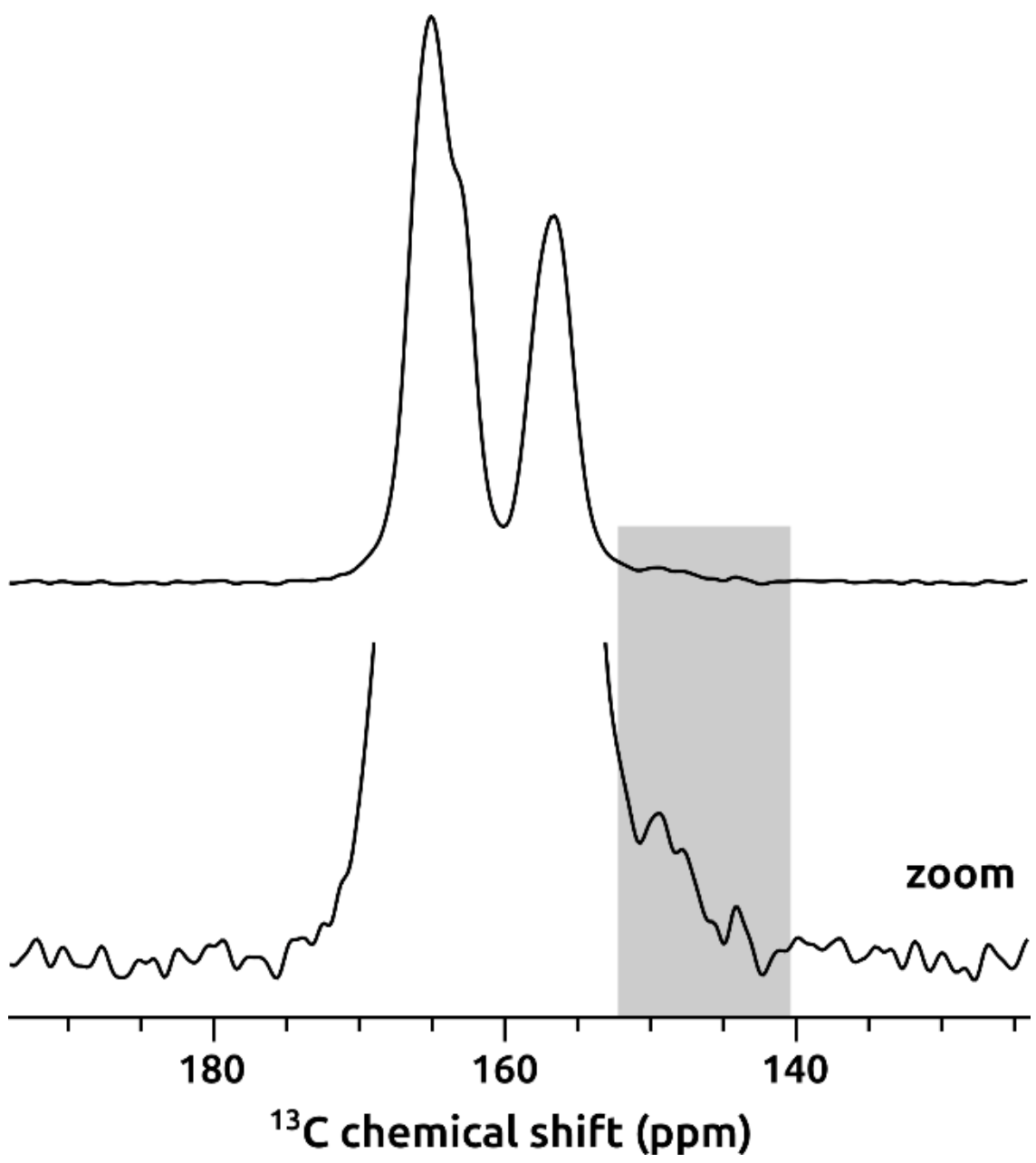

Figure S19: Zoom of ^{13}C ssNMR spectrum.

4.7 X-ray photoelectron spectroscopy (XPS)

XPS spectra were collected on a Prevac spectrometer equipped with a hemispherical VG SCIENTA R3000 analyser (pass energy of 100 eV) and a monochromatized aluminium source AlKα (1486.6 eV). Binding energies were calibrated using the C 1s line at 284.8 eV. After a Shirley background subtraction, raw spectra were fitted using Gaussian-Lorentzian peak shapes in the Casa XPS software. The surface composition of Mn@f-g-C_3N_4 was investigated by XPS, with spectra recorded in the C 1s, N 1s, and Mn 2p regions, as well as survey spectra (**Figures 3c-f**). In the C 1s and N 1s regions, the fresh Mn@f-g-C_3N_4 exhibits typical components corresponding to the presence of the C_3N_4 phase on the surface.

The C 1s spectrum is dominated by an intense peak at 288.0 eV, attributed to sp^2-bonded C atoms in N–C = N units, and a smaller contribution at 284.8 eV, arising from adventitious carbon contamination.

In the N 1s region, three distinct peaks are observed at 398.4 eV (sp^2-hybridized N atoms in C–N = C), 400.0 eV (bridging N atoms in N–$(C)_3$ species), and 401.0 eV (N atoms in amino groups). Additionally, a broad signal centered at approximately 404 eV is detected. This feature is may be ascribed to π-excitation of nitrogen atoms within graphitic structures.

No Mn-related signals are detected in the Mn 2p region, which indicates that manganese is present in very low concentrations (< 0.1 at.%) and is highly dispersed on the surface. The survey spectrum likewise does not reveal any additional surface components.

In the post-reaction samples, the survey spectrum does show signals of Cl (peaks at 271 eV and 200 eV) and O (peak at 531 eV), indicating that these elements bind to the catalyst during the reaction.

After the catalytic reaction, the characteristic features of the C_3N_4 structure remain visible, confirming its preservation. However, the intensity of the main peaks in both the C 1s and N 1s regions decreases, while the contribution of the carbon signal at 284.8 eV increases markedly. This change suggests the formation of a carbonaceous deposit on the surface.

The quantitative XPS analysis of the C 1s spectra provides valuable insight into the surface evolution of the catalyst during consecutive reaction cycles. The relative contribution of the C–C/C=C component, associated with carbonaceous species deposited on the surface, increases from 4% in the fresh sample to 13% after the first run and 17% after the tenth run, while the contribution of carbon originating from the C_3N_4 framework correspondingly decreases from 96% to 83%. This trend indicates that the formation of carbon deposits occurs primarily during the first catalytic cycle and that the surface composition stabilizes thereafter. The slight further increase after extended use suggests only limited additional accumulation of carbonaceous material. These findings are consistent with the maintained catalytic performance observed in subsequent runs.

4.8 High-angle annular dark-field scanning transmission electron microscopy (HAADF-STEM) and energy-dispersive X-ray (EDX) spectroscopy

EDX spectroscopy was performed at 20 KeV on a FEI Tecnai Osiris electron microscope. High resolution (HR) STEM images were captured on a FEI Titan Themis electron microscope at 300 keV. High-angle annular dark field imaging mode was used for all STEM imaging.

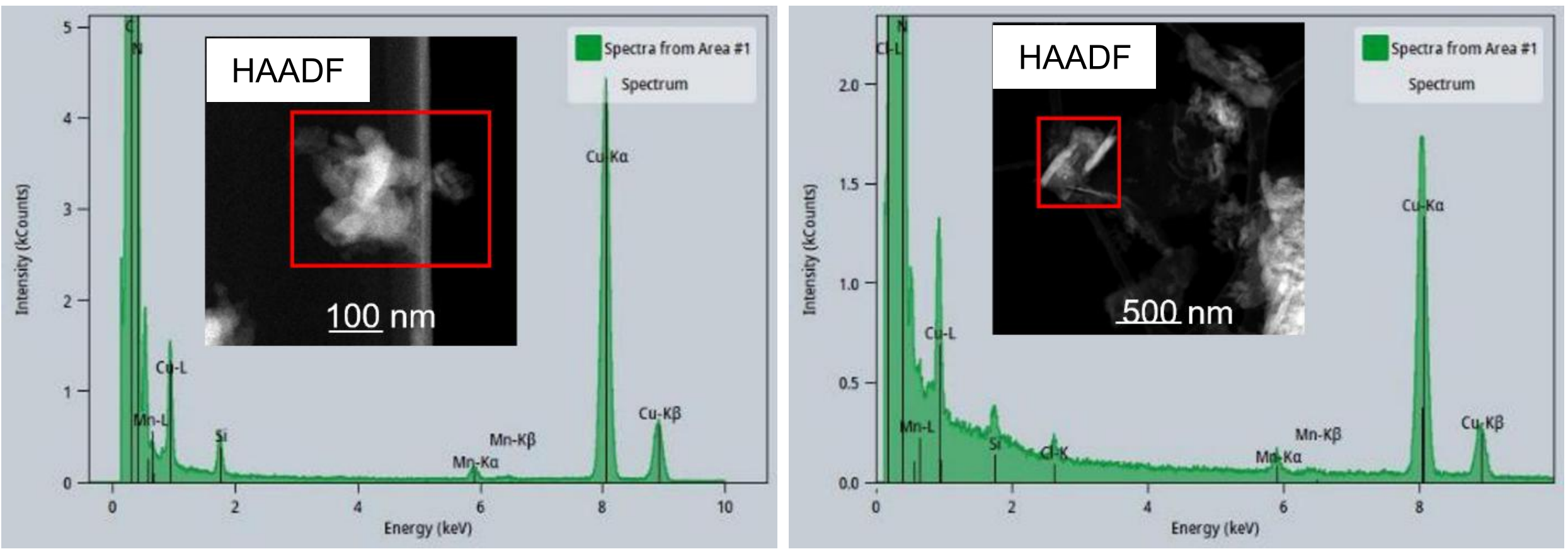

Figure S20: EDX spectra of fresh (left) and used (right) Mn@f-g-C_3N_4.

4.9 X-ray absorption spectroscopy (XAS)

XAS was measured at the ALBA Synchrotron Radiation Facility (CLÆSS beamline)[4], using a double-crystal Si(111) monochromator (proposal #20260380031). XAS spectra were collected at room temperature in air in transmission mode for the standard reference compounds (Mn foil, MnO_2 and Mn_2O_3) and in fluorescence mode for the samples using 4-channel silicon drift detector (SDD). The samples were prepared as 5 mm pellets of pure material. EXAFS fitting was conducted using DEMETER software in the k-range of 2.6–8.3 Å^{-1} with k^1, k^2, and k^3 weightings applied simultaneously.[5] The fitting was carried out over an R-range of 1–3 Å considering Mn–N scattering path.

Table S3. EXAFS fitting parameters at the Mn *K*-edge with Mn-N scattering path

Mn@f-g-C_3N_4	C. N.	Δ*E* (eV)	R (Å)	σ^2	R-factor
Fresh	5.78 ± 1.02	1.60 ± 1.69	2.16 ± 0.16	0.008 ± 0.003	0.0197
Used	4.69 ± 1.19	3.69 ± 2.35	2.17 ± 0.17	0.004 ± 0.005	0.0424

4.10 Nuclear magnetic resonance spectroscopy

^1H NMR (400 MHz) and ^{13}C NMR (101 MHz) spectra were recorded using a Bruker Avance III Fourier Transform NMR spectrometer at 303 K unless stated otherwise, using tetramethylsilane (TMS) or DMSO-d6 as internal standard (^1H: δ 0.00 ppm and ^{13}C (proton decoupled): δ 0.00 ppm in $CDCl_3$; ^1H: δ 2.50 ppm and ^{13}C (proton decoupled): δ 39.52 in DMSO-d6). Chemical shifts (δ) are expressed in ppm and coupling constants (*J*) in Hertz (Hz). Splitting patterns are reported as: s (singlet), d (doublet), t (triplet), q

(quadruplet), p (pentuplet), m (multiplet) or combinations thereof. Integration of the signals is presented as the number of hydrogen atoms.

1-Chloro-2,4-dimethoxybenzene (3a)

Following procedure 3.3 with 1,3-dimethoxybenzene (26.2 μL, 0.2 mmol), the crude product was purified using preparative TLC with petroleum ether/ethyl acetate (9/1) as the eluent. **^{1}H NMR** (400 MHz, $CDCl_3$) δ 7.24 (d, *J* = 8.7 Hz, 1H), 6.51 (d, *J* = 2.7 Hz, 1H), 6.43 (dd, *J* = 8.7, 2.7 Hz, 1H), 3.87 (s, 3H), 3.79 (s, 3H). **^{13}C NMR** (101 MHz, $CDCl_3$) δ 159.5, 155.7, 130.2, 114.3, 105.2, 100.1, 56.1, 55.6.

1,5-Dichloro-2,4-dimethoxybenzene (3a-D)

Following procedure 3.3 with 1,3-dimethoxybenzene (26.2 μL, 0.2 mmol), the crude product was purified using preparative TLC with petroleum ether/ethyl acetate (9/1) as the eluent. **^{1}H NMR** (400 MHz, $CDCl_3$) δ 7.35 (s, 1H), 6.53 (s, 1H), 3.90 (s, 6H). **^{13}C NMR** (101 MHz, $CDCl_3$) δ 154.7, 130.7, 114.3, 98.0, 56.7.

4-Chloro-1,2-dimethoxybenzene (3b)

Following procedure 3.3 with 1,2-dimethoxybenzene (25.6 μL, 0.2 mmol), the crude product was purified using preparative TLC with petroleum ether/ethyl acetate (9/1) as the eluent. **^{1}H NMR** (400 MHz, $CDCl_3$) δ 6.90 - 6.84 (m, 2H), 6.78 (d, *J* = 8.5 Hz, 1H), 3.87 (s, 3H), 3.86 (s, 3H). **^{13}C NMR** (101 MHz, $CDCl_3$) δ 149.6, 147.9, 125.7, 120.3, 112.2, 112.0, 56.1, 56.1.

1-(Benzyloxy)-4-chlorobenzene (3c)

Following procedure 3.3 with benzyl phenyl ether (36.8 mg, 0.2 mmol), the crude product was purified using preparative TLC with petroleum ether/ethyl acetate (9/1) as the eluent. **^{1}H NMR** (400 MHz, $CDCl_3$) δ 7.45 - 7.29 (m, 5H), 7.25 - 7.20 (m, 2H), 6.89 (m, 2H), 5.03 (s, 2H). **^{13}C NMR** (101 MHz, $CDCl_3$) δ 157.4, 136.6, 129.4, 128.6, 128.1, 127.4, 125.9, 116.2, 70.3.

5-Chloro-2-methoxy-1,1'-biphenyl (3d)

Following procedure 3.3 with 2-methoxybiphenyl (36.0 μL, 0.2 mmol), the crude product was purified using preparative TLC with petroleum ether/ethyl acetate (19/1) as the eluent. **^{1}H NMR** (400 MHz, DMSO-d6) δ 7.48 - 7.46 (m, 2H), 7.43 - 7.37 (m, 3H), 7.36 - 7.32 (m, 1H), 7.29 (d, *J* = 2.7 Hz, 1H), 7.13 (d, *J* = 8.8 Hz, 1H), 3.76 (s, 3H). **^{13}C NMR** (101 MHz, DMSO-d6) δ 155.0, 136.7, 131.6, 129.6, 129.2, 128.2, 128.1, 127.3, 124.4, 113.5, 55.9.

1-Chloro-4-ethoxybenzene (3e)

Following procedure 3.3 with phenetole (25.3 μL, 0.2 mmol), the crude product was purified using preparative TLC with petroleum ether/ethyl acetate (30/1) as the eluent. **^{1}H NMR** (400 MHz, $CDCl_3$) δ 7.23 - 7.19 (m, 2H), 6.82 - 6.78 (m, 1H), 3.99 (q, *J* = 7.0 Hz, 2H), 1.39 (t, *J* = 7.0 Hz, 3H). **^{13}C NMR** (101 MHz, $CDCl_3$) δ 157.6, 129.3, 125.4, 115.8, 63.8, 14.8.

1-Chloro-4,5-dimethoxy-2-methylbenzene (3f)

Following procedure 3.3 with 1,2-dimethoxy-4-methylbenzene (29.0 μL, 0.2 mmol), the crude product was purified using preparative TLC with petroleum ether/ethyl acetate (4/1) as the eluent. **^{1}H NMR** (400 MHz, $CDCl_3$) δ 6.84 (s, 1H), 6.71 (s, 1H), 3.85 (s, 3H), 3.84 (s, 3H), 2.30 (s, 3H). **^{13}C NMR** (101 MHz, $CDCl_3$) δ 147.8, 147.8, 127.8, 125.1, 113.8, 112.6, 56.3, 56.2, 19.6.

4-Chloro-2-isopropyl-5-methylphenol (3g)

Following procedure 3.3 with thymol (30.0 mg, 0.2 mmol), the crude product was purified using preparative TLC with petroleum ether/ethyl acetate (9/1) as the eluent. **^{1}H NMR** (400 MHz, $CDCl_3$) δ 7.12 (s, 1H), 6.61 (s, 1H), 4.69 (s, 1H), 3.12 (hept, *J* = 6.9 Hz, 1H), 2.27 (s, 3H), 1.22 (d, *J* = 6.9 Hz, 6H).**^{13}C NMR** (101 MHz, $CDCl_3$) δ 151.2, 134.0, 133.7, 126.9, 125.9, 117.7, 77.3, 77.0, 76.7, 26.9, 22.5, 19.5.

1-Chloro-2-methoxynaphthalene (3h)

Following procedure 3.3 with 2-methoxynaphtalene (31.6 mg, 0.2 mmol), the crude product was purified using preparative TLC with petroleum ether/ethyl acetate (20/1) as the eluent. **^{1}H NMR** (400 MHz, $CDCl_3$) δ 8.21 (dd, *J* = 8.6, 0.7 Hz, 1H), 7.78 - 7.73 (m, 1H), 7.55 (ddd, *J* = 8.5, 6.8, 1.3 Hz, 1H), 7.38 (ddd, *J* = 8.1, 6.8, 1.2 Hz, 1H), 7.27 (d, *J* = 9.0 Hz, 1H), 4.00 (s, 3H). **^{13}C NMR** (101 MHz, $CDCl_3$) δ 152.6, 131.9, 129.5, 128.0, 128.0, 127.4, 124.3, 123.5, 116.9, 113.7, 57.0.

3-Chloro-4-hydroxy-5-methoxybenzaldehyde (Cl-Vanillin) (3i)

Following procedure 3.3 with vanillin (30.4 mg, 0.2 mmol), the crude product was purified using flash column chromatography with petroleum ether/ethyl acetate (3/1) as the eluent. **^{1}H NMR** (400 MHz, DMSO-d6) δ 10.64 (s, 1H), 9.77 (s, 1H), 7.57 (d, *J* = 1.8 Hz, 1H), 7.39 (d, *J* = 1.8 Hz, 1H), 3.91 (s, 3H). **^{13}C NMR** (101 MHz, DMSO-d6) δ 190.4, 148.9, 148.8, 128.2, 125.6, 120.1, 109.2, 56.3. **HRMS** (ESI^+, *m/z*) [$M+H^+$] calcd. for $C_8H_7O_3Cl$ 187.0156, found 187.0157.

5-Chloro-2,4,6-trimethoxypyrimidine (3j)

Following procedure 3.3 with 2,4,6-trimethoxypyrimidine (30.4 mg, 0.2 mmol), the crude product was purified using preparative TLC with a dichloromethane/ethyl acetate (40/1) as the eluent. **^{1}H NMR** (400 MHz, $CDCl_3$) δ 4.01 (s, 6H), 3.95 (s, 3H). **^{13}C NMR** (101 MHz, $CDCl_3$) δ 166.8, 161.7, 92.5, 55.1, 55.0. **HRMS** (ESI^+, *m/z*) [$M+H^+$] calcd. for $C_7H_9N_2O_3Cl$ 205.0374, found 205.0378.

3-Chloro-2,6-dimethoxypyridine (3k)

General procedure 3.3 was followed with 2,6-dimethoxypyridine (26.4 μL, 0.2 mmol), the crude product was purified using preparative TLC with a dichloromethane/dichloromethane (4/1) as the eluent. **^{1}H NMR** (400 MHz, $CDCl_3$) δ 7.49 (d, *J* = 8.3 Hz, 1H), 6.27 (d, *J* = 8.3 Hz, 1H), 4.01 (s, 3H), 3.91 (s, 3H). **^{13}C NMR** (101 MHz, $CDCl_3$) δ 161.3, 157.6, 140.6, 107.9, 102.0, 54.1, 53.8. **HRMS** The product could not be ionized and, therefore, measured using our equipment.

5-Chloro-1,3-dimethylpyrimidine-2,4(1H,3H)-dione (3l)

General procedure 3.3 was followed with 1,3-dimethyluracil (28.0 mg, 0.2 mmol), the crude product was purified using flash column chromatography with petroleum ether/ethyl acetate (1/1) as the eluent. **^{1}H**

NMR (400 MHz, $CDCl_3$) δ 7.41 (s, 1H), 3.43 (s, 3H), 3.41 (s, 3H). **^{13}C NMR** (101 MHz, $CDCl_3$) δ 159.5, 150.9, 139.8, 108.1, 37.3, 29.0. **HRMS** (ESI$^+$, *m/z*) [M+H$^+$] calcd. for $C_6H_7N_2O_2Cl$ 175.0269, found 175.0274.

4-Chloro-1-phenylpyrazole (3m)

General procedure 3.3 was followed with 1-phenylpyrazole (26.4 µL, 0.2 mmol), the crude product was purified using preparative TLC with petroleum ether/ethyl acetate (4/1) as the eluent. **^{1}H NMR** (400 MHz, $CDCl_3$) δ 7.89 (d, *J* = 0.7 Hz, 1H), 7.64 - 7.61 (m, 3H), 7.47 – 7.41 (m, 2H), 7.30 (tt, *J* = 7.5, 1.2 Hz, 1H). **^{13}C NMR** (101 MHz, $CDCl_3$) δ 139.8, 139.5, 129.5, 127.0, 124.8, 119.0, 112.4. **HRMS** (ESI$^+$, *m/z*) [M+H$^+$] calcd. for $C_9H_7N_2Cl$ 179.0371, found 179.0377.

8-Chloro-1,3,7-trimethyl-3,7-dihydro-1H-purine-2,6-dione (Cl-Caffeine) (3n)

General procedure 3.3 was followed with caffeine (38.8 mg, 0.2 mmol), the crude product was purified using preparative TLC with petroleum ether/ethyl acetate (9/11) as the eluent. **^{1}H NMR** (400 MHz, $CDCl_3$) δ 3.95 (s, 1H), 3.54 (s, 1H), 3.40 (s, 1H). **^{13}C NMR** (101 MHz, $CDCl_3$) δ 154.6, 151.3, 147.1, 139.0, 108.3, 32.7, 29.8, 28.0. **HRMS** (ESI$^+$, *m/z*) [M+H$^+$] calcd. for $C_8H_9N_4O_2Cl$ 229.0487, found 229.0487.

4,5-Dichloro-7H-pyrrolo[2,3-d]pyrimidine (3o)

General procedure 3.3 was followed with 6-chloro-7-deazapurine (30.7 mg, 0.2 mmol), the crude product was purified using flash column chromatography with petroleum ether/ethyl acetate (4/1) as the eluent. **^{1}H NMR** (400 MHz, DMSO-d6) δ 12.86 (bs, 1H), 8.62 (s, 1H), 7.88 (d, *J* = 2.6 Hz, 2H). **^{13}C NMR** (101 MHz, DMSO-d6) δ 151.0, 150.4, 149.8, 126.0, 112.5, 101.5. **HRMS** (ESI$^+$, *m/z*) [M+H$^+$] calcd. for $C_6H_3N_3Cl_2$ 187.9777, found 187.9783.

2-Chloro-5-phenylthiophene (3p)

General procedure 3.3 was followed with 2-phenylthiophene (32.0 mg, 0.2 mmol), the crude product was purified using flash column chromatography using 100% petroleum ether as the eluent. **^{1}H NMR** (400 MHz, $CDCl_3$) δ 7.48 - 7.45 (m, 2H), 7.45 - 7.31 (m, 2H), 7.27 - 7.23 (m, 1H), 7.01 (d, *J* = 3.9 Hz, 1H), 6.84 (d, *J* = 3.9 Hz, 1H). **^{13}C NMR** (101 MHz, $CDCl_3$) δ 142.9, 133.7, 129.1, 129.0, 127.8, 127.1, 125.5, 122.2.

5-(4-Chloro-2,5-dimethylphenoxy)-2,2-dimethylpentanoic acid (Cl-Gemfibrozil) (3q)

General procedure 3.3 was followed with gemfibrozil (50.1 mg, 0.2 mmol), the crude product was purified using flash column chromatography with petroleum ether/ethyl acetate (10/1) as the eluent. **^{1}H NMR** (400 MHz, $CDCl_3$) δ 7.06 (s, 1H), 6.62 (s, 1H), 3.90 (t, *J* = 5.9 Hz, 2H), 2.30 (s, 3H), 2.14 (s, 3H), 1.82 - 1.70 (m, 2H), 1.25 (s, 6H). **^{13}C NMR** (101 MHz, $CDCl_3$) δ 184.7, 155.6, 133.7, 130.6, 125.9, 124.9, 113.5, 68.3, 42.0, 36.8, 25.1, 25.0, 20.0, 15.5. **HRMS** (ESI$^+$, *m/z*) [M+Na$^+$] calcd. for $C_{15}H_{21}O_3Cl$ 307.1071, found 307.1071.

5-((4-Chloro-3,5-dimethylphenoxy)methyl)oxazolidin-2-one (Cl-Metaxolone) (3r)

General procedure 3.3 was followed with metaxolone (44.3 mg, 0.2 mmol) and 1.2 equiv. of NCS **2** (32.0 mg, 0.24 mmol), the crude product was purified using flash column chromatography with petroleum ether/ethyl acetate (1/1) as the eluent. **^{1}H NMR** (400 MHz, $CDCl_3$) δ 6.65 (s, 2H), 6.10 (bs, 1H), 4.96 - 4.92 (m, 1H), 4.09 (d, *J* = 4.9 Hz, 2H), 3.76 (td, *J* = 8.8, 0.7 Hz, 1H), 3.58 (ddd, *J* = 8.8, 6.2, 0.7 Hz, 1H), 2.33 (s, 6H).

^{13}C NMR (101 MHz, $CDCl_3$) δ 159.6, 156.0, 137.4, 127.2, 114.7, 74.2, 68.3, 42.7, 20.9. **HRMS** (ESI$^+$, *m/z*) [M+Na$^+$] calcd. for $C_{12}H_{14}NO_3Cl$ 278.0554, found 278.0556.

(S)-N-(2-(1-(2-Chloro-5-ethoxy-4-methoxyphenyl)-2-(methylsulfonyl)ethyl)-1,3-dioxoisoindolin-4-yl)acetamide (Cl-Apremilast) (3s)

General procedure 3.3 was followed with apremilast (92.1 mg, 0.2 mmol), the crude product was purified using flash column chromatography with petroleum ether/ethyl acetate (1/1) as the eluent. **^{1}H NMR** (400 MHz, DMSO-d6) δ 9.68 (s, 1H), 8.43 (dd, *J* = 8.4, 0.8 Hz, 1H), 7.79 (dd, *J* = 8.4, 7.3 Hz, 1H), 7.57 (dd, *J* = 7.3, 0.8 Hz, 1H), 7.40 (s, 1H), 7.05 (s, 1H), 6.16 (dd, *J* = 10.5, 4.0 Hz, 1H), 4.38 (dd, *J* = 14.5, 10.5 Hz, 1H), 4.09 (q, *J* = 7.0 Hz, 2H), 4.01 (dd, *J* = 14.5, 4.0 Hz, 1H), 3.77 (s, 3H), 3.05 (s, 3H), 2.19 (s, 3H), 1.35 (t, *J* = 7.0 Hz, 3H). **^{13}C NMR** (101 MHz, DMSO-d6) δ 169.24, 167.60, 166.83, 149.73, 146.69, 136.47, 135.92, 131.33, 126.17, 125.86, 123.58, 118.23, 116.63, 114.61, 112.73, 64.30, 55.93, 52.82, 44.57, 40.95, 40.15, 39.94, 39.73, 39.52, 39.31, 39.10, 38.89, 24.17, 14.53. **HRMS** (ESI$^+$, *m/z*) [M+H$^+$] calcd. for $C_{22}H_{23}N_2O_7ClS$ 495.0987, found 495.0997.

4-Amino-3-chloro-N-(5,6-dimethoxypyrimidin-4-yl)benzenesulfonamide (Cl-Sulfadoxine) (3t)

General procedure 3.3 was followed with sulfadoxine (62.1 mg, 0.2 mmol), the crude product was purified using flash column chromatography with petroleum ether/ethyl acetate (2/3) as the eluent. **^{1}H NMR** (400 MHz, DMSO-d6) δ 10.76 (bs, 1H), 8.13 (s, 1H), 7.78 (d, *J* = 2.2 Hz, 1H), 7.62 (dd, *J* = 8.6, 2.2 Hz, 1H), 6.82 (d, *J* = 8.7 Hz, 1H), 6.27 (bs, 2H), 3.90 (s, 3H), 3.69 (s, 3H). **^{13}C NMR** (101 MHz, DMSO-d6) δ 161.5, 150.5, 148.8, 129.2, 128.0, 127.0, 126.7, 115.3, 113.8, 60.2, 54.0. **HRMS** (ESI$^+$, *m/z*) [M+H$^+$] calcd. for $C_{12}H_{13}N_4O_4ClS$ 345.0419, found 345.0426.

Methyl (S)-2-((tert-butoxycarbonyl)amino)-3-(3-chloro-4-hydroxyphenyl)propanoate (Cl-Boc-Tyr-OMe) (3u)

General procedure 3.3 was followed with Boc-L-tyrosine methyl ester (59.1 mg, 0.2 mmol), the crude product was purified using flash column chromatography with petroleum ether/ethyl acetate (6/1) as the eluent. **^{1}H NMR** (400 MHz, $CDCl_3$) δ 7.08 (s, 1H), 6.93 – 6.88 (m, 2H), 6.21 (s, 1H), 5.10 (d, *J* = 8.3 Hz, 1H), 4.53 (d, *J* = 7.1 Hz, 1H), 3.72 (s, 3H), 3.06 - 2.91 (m, 2H), 1.43 (s, 9H). **^{13}C NMR** (101 MHz, $CDCl_3$) δ 172.3, 155.2, 150.8, 129.9, 129.1, 129.0, 120.0, 116.5, 80.2, 54.5, 52.3, 37.3, 28.3. **HRMS** (ESI$^+$, *m/z*) [M+Na$^+$] calcd. for $C_{15}H_{20}NO_5Cl$ 352.0922, found 352.0919.

2-(5-Chloro-6-methoxynaphthalen-2-yl)propanoic acid (Cl-Naproxen) (3v)

General procedure 3.3 was followed with naproxen (46.1 mg, 0.2 mmol), the crude product was purified using flash column chromatography with petroleum ether/ethyl acetate (7/3) as the eluent. **^{1}H NMR** (400 MHz, DMSO-d6) δ 8.05 (d, *J* = 8.8 Hz, 1H), 7.94 (d, *J* = 9.1 Hz, 1H), 7.83 (d, *J* = 1.5 Hz, 1H), 7.57 (dd, *J* = 8.8, 1.8 Hz, 1H), 7.54 (d, *J* = 9.1 Hz, 1H), 3.98 (s, 3H), 3.85 (q, *J* = 7.1 Hz, 1H), 1.46 (d, *J* = 7.1 Hz, 2H). **^{13}C NMR** (101 MHz, DMSO-d6) δ 175.2, 152.3, 137.2, 130.0, 129.0, 128.2, 128.0, 126.2, 122.8, 114.8, 114.5, 56.7, 44.4, 18.3. **HRMS** (ESI$^+$, *m/z*) [M+Na$^+$] calcd. for $C_{14}H_{13}O_3Cl$ 287.0445, found 287.0445.

(5S,5aR,8aR,9R)-5-(2-Chloro-3,4,5-trimethoxyphenyl)-9-hydroxy-5,8,8a,9-tetrahydrofuro[3',4':6,7]naphtho[2,3-d][1,3]dioxol-6(5aH)-one (Cl-Podophyllotoxin) (3w)

General procedure 3.3 was followed with podophyllotoxin (82.9 mg, 0.2 mmol), the crude product was purified using flash column chromatography with petroleum ether/ethyl acetate (1/9) as the eluent. **^{1}H NMR** (400 MHz, $CDCl_3$) δ 7.09 (s, 1H), 6.36 (s, 1H), 6.20 (s, 1H), 5.93 (s, 2H), 5.20 (d, *J* = 5.1 Hz, 1H), 4.78 (t, *J* = 7.4 Hz, 1H), 4.64 (dd, *J* = 8.7, 6.4 Hz, 1H), 4.11 - 4.04 (m, 1H), 3.91 (s, 3H), 3.86 (s, 3H), 3.65 (s, 3H), 3.03 - 2.87 (m, 2H), 2.49 (d, *J* = 6.4 Hz, 1H). **^{13}C NMR** (101 MHz, $CDCl_3$) δ 173.2, 151.6, 150.0, 147.9, 147.6, 142.8, 133.7, 132.7, 131.4, 122.8, 110.6, 109.7, 105.7, 101.5, 71.9, 71.0, 61.1, 61.1, 56.5, 53.4, 44.2, 41.8. **HRMS** (ESI^+, *m/z*) [$M+Na^+$] calcd. for $C_{22}H_{21}O_8Cl$ 471.0817, found 471.0824.

5 Computational modeling

Quantum Mechanics (QM) simulations were performed using the Gaussian 16 program.[6] Local optimizations were performed in acetonitrile by applying a continuum through the solvation model based on solute electron density (SMD) approach, in agreement with the experiments.[7] We employed density functional theory (DFT) level with the B3LYP hybrid functional, Grimme DFT-D3(BJ) empirical dispersion, the 6-31G(d,p) basis set for the C, N, O, F, and H atoms, and the def2-TZVPD basis set for the Mn center (Fe).[8] Saddle points were located using the Synchronous Transit-Guided Quasi-Newton (STQN).[9] Time-dependent DFT (TDDFT) calculations on the optimized ground-state geometries (by requesting 100 excitations) were carried out to simulate the UV-vis spectra of the most stable complexes.

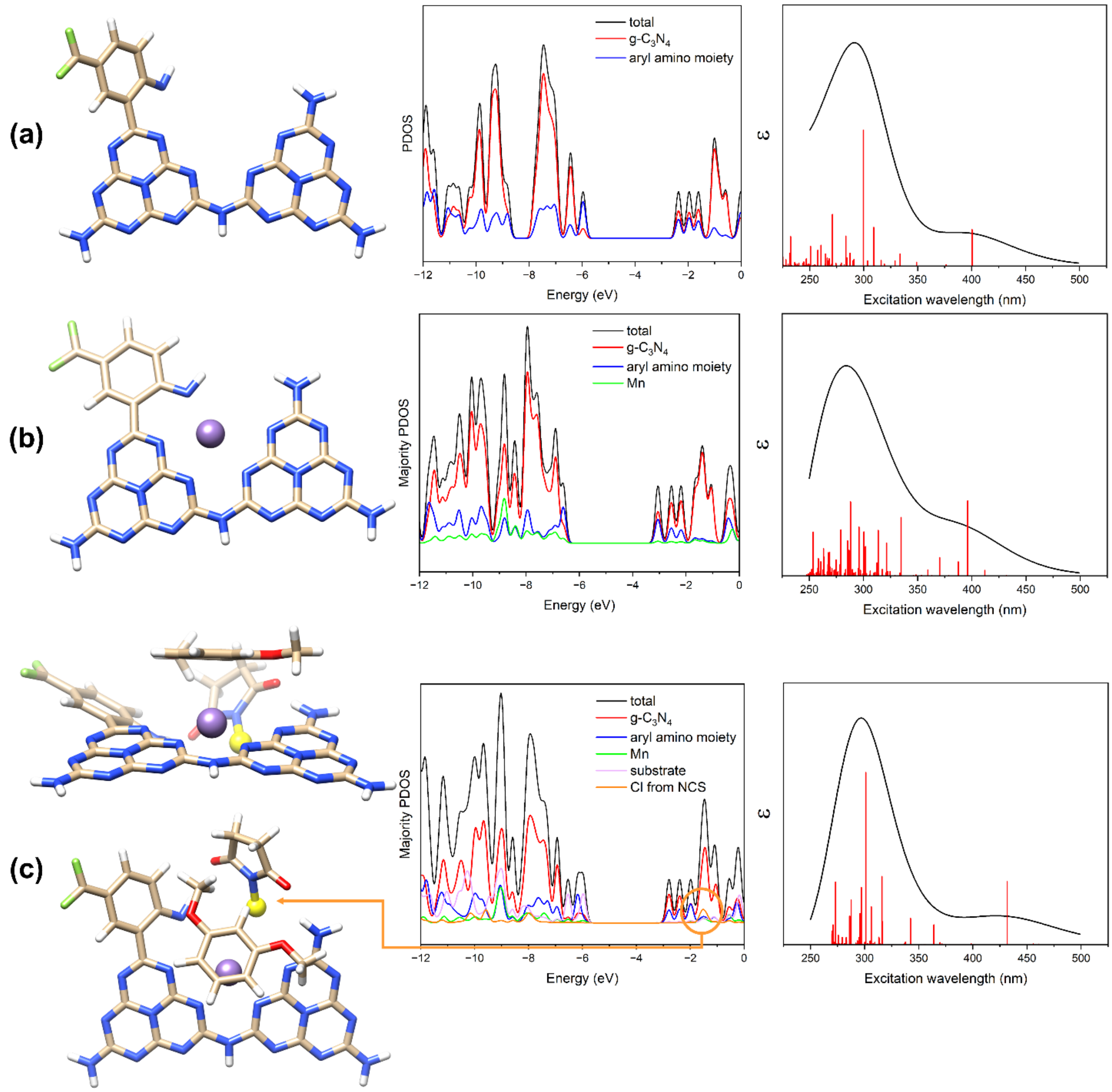

Figure S21: Relaxed geometries, Projected Density of States (pDOS), and simulated absorption spectra of the models corresponding to a) isolated g-C_3N_4 support functionalized with the =CF_2 aryl moiety; b) the same system in (a) with the addition of chelated Mn SAC;

c) the same system in (b) in the presence of substrate **3a** and NCS **2** species. Colour code: C (light brown), N (blue), F (green), O (red), H (white), Cl (yellow), Mn (purple).

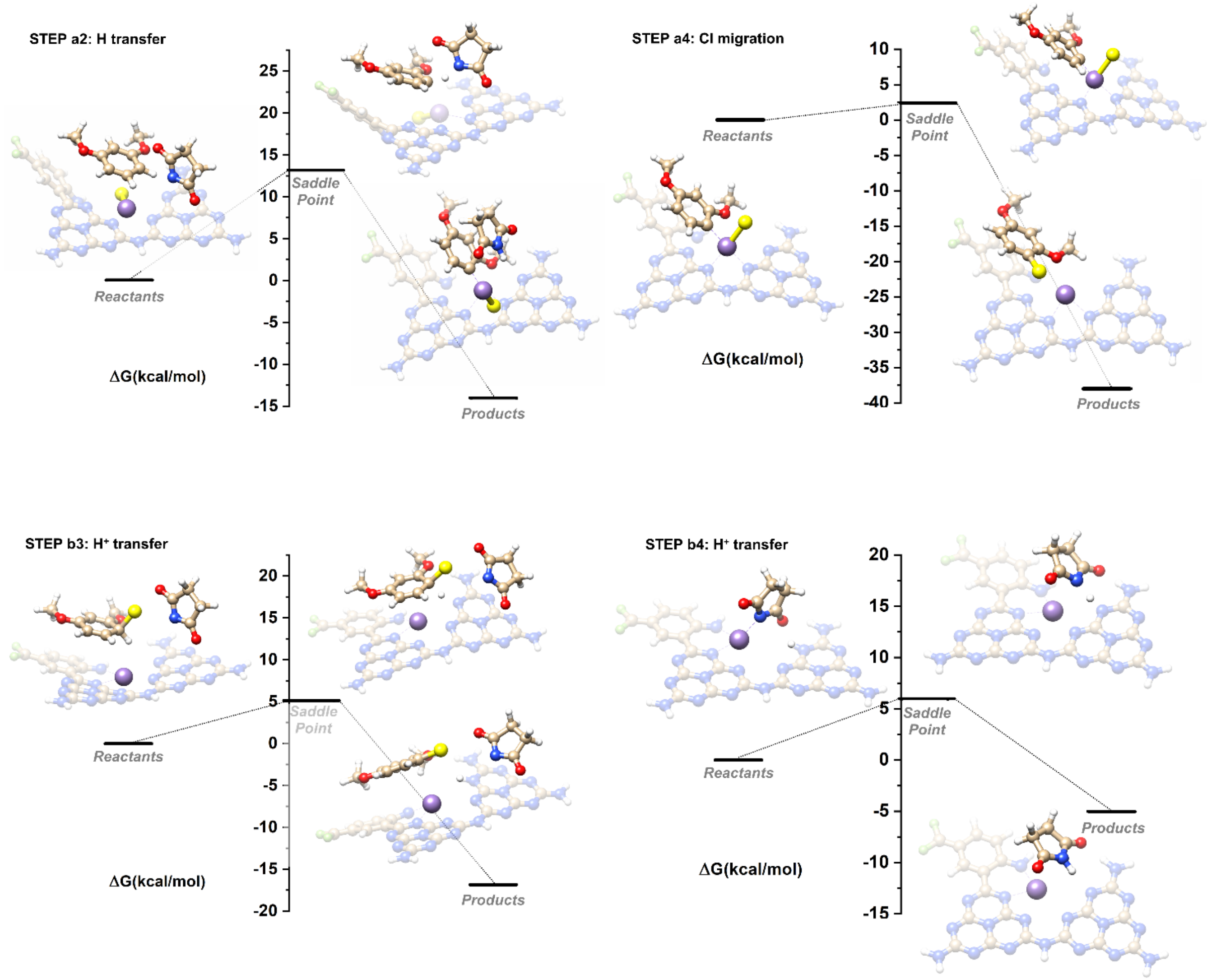

Figure S22: Free energy diagrams, reporting the structure of reactants, products, and saddle points, of Steps a2 and a4 from Path a, and of Steps b3 and n4 from Path b (see Figure 6 of the main text). Colour code: C (light brown), N (blue), F (green), O (red), H (white), Cl (yellow), Mn (purple).

6 References

[1] R. Samanta, S. Singh, R. Rakheja, S. Singh, M. V. Mane, C. K. Hazra, *Adv. Synth. Catal.* **2026**, *368*, e70574.
[2] J. Tauc, R. Grigorovici, A. Vancu, *Phys. Status Solidi B Basic Res.* **1966**, *15*, 627–637.
[3] K. Gelderman, L. Lee, S. W. Donne, *J. Chem. Educ.* **2007**, *84*, 685–688.
[4] L. Simonelli, C. Marini, W. Olszewski, M. Ávila Pérez, N. Ramanan, G. Guilera, V. Cuartero, K. Klementiev, *Cogent Phys.* **2016**, *3*, 1231987–1231996.
[5] B. Ravel, M. Newville, *J. Synchrotron Rad.* **2005**, *12*, 537–541.
[6] M. J. Frisch, et al., *Gaussian 16 Rev. C.01* **2016**, Gaussian, Inc., Wallingford, CT.
[7] A. V. Marenich, C. J. Cramer, D. G. Truhlar, *J. Phys. Chem. B* **2009**, *113*, 6378–6396.
[8] L. E. Roy, P. J. Hay, R. L. Martin, *J. Chem. Theory Comput.* **2008**, *4*, 1029–1031.
[9] C. Peng, H. Bernhard Schlegel, *Isr. J. Chem.* **1993**, *33*, 449–454.

7 Spectra

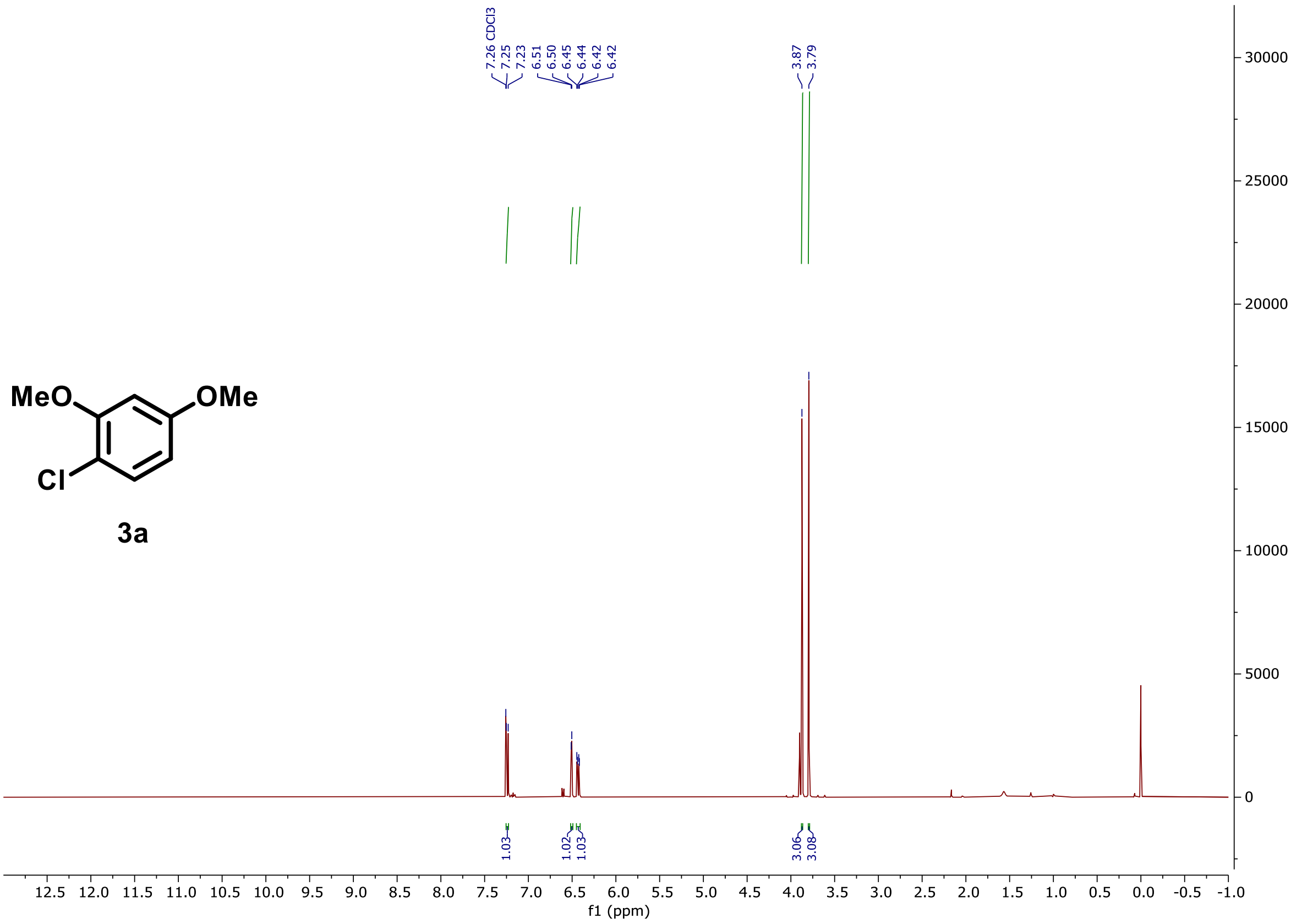

MeO
OMe
Cl
3a
7.26 CDCl3
7.25
7.23
6.51
6.50
6.45
6.44
6.42
6.42
3.87
3.79
1.03
1.02
1.03
3.06
3.08
f1 (ppm)

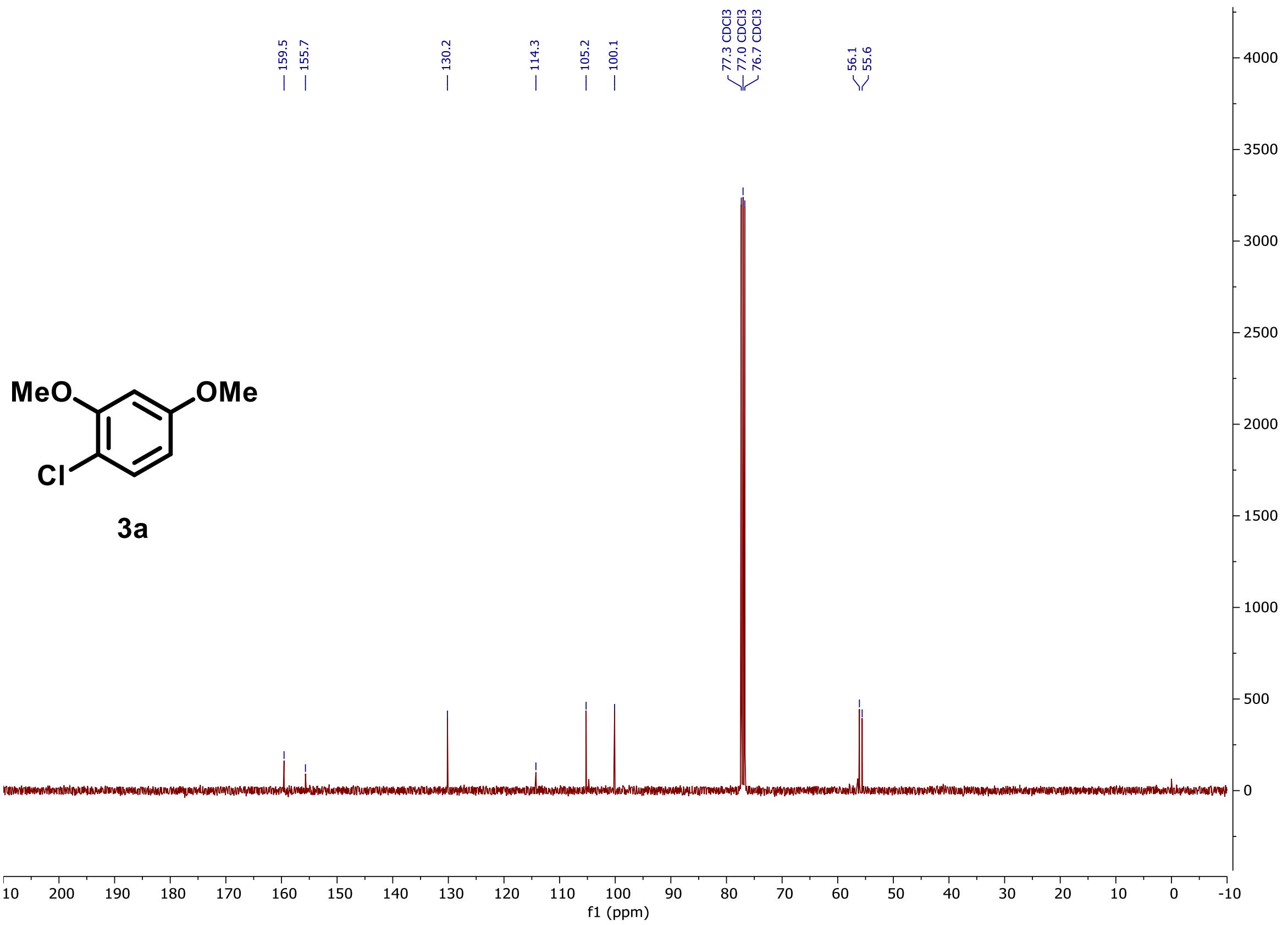

MeO
OMe
Cl
3a
159.5
155.7
130.2
114.3
105.2
100.1
77.3 CDCl3
77.0 CDCl3
76.7 CDCl3
56.1
55.6
f1 (ppm)

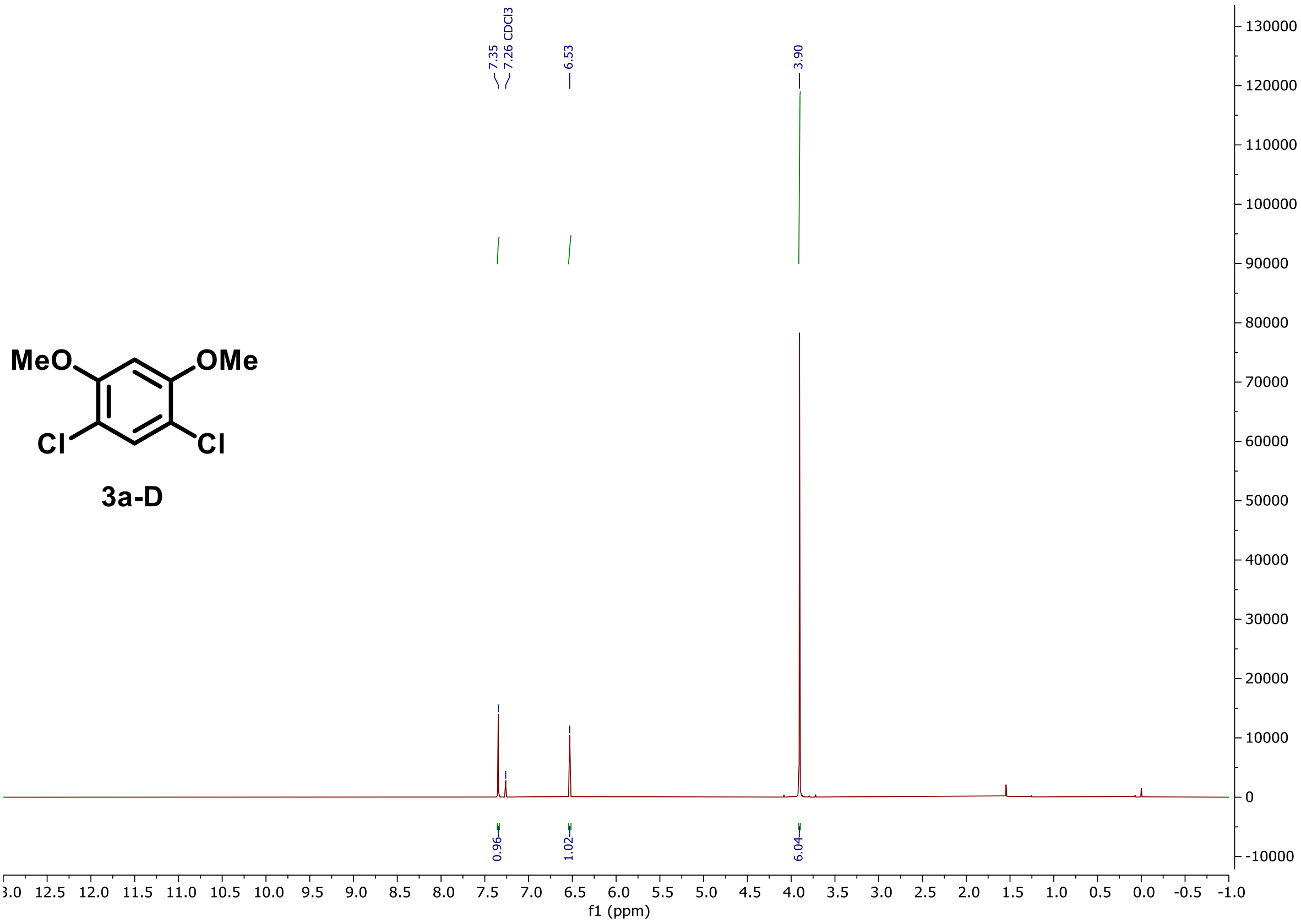

7.35
7.26 CDCl3
6.53
3.90
MeO
OMe
Cl
Cl
0.96
1.02
6.04
f1 (ppm)

3a-D

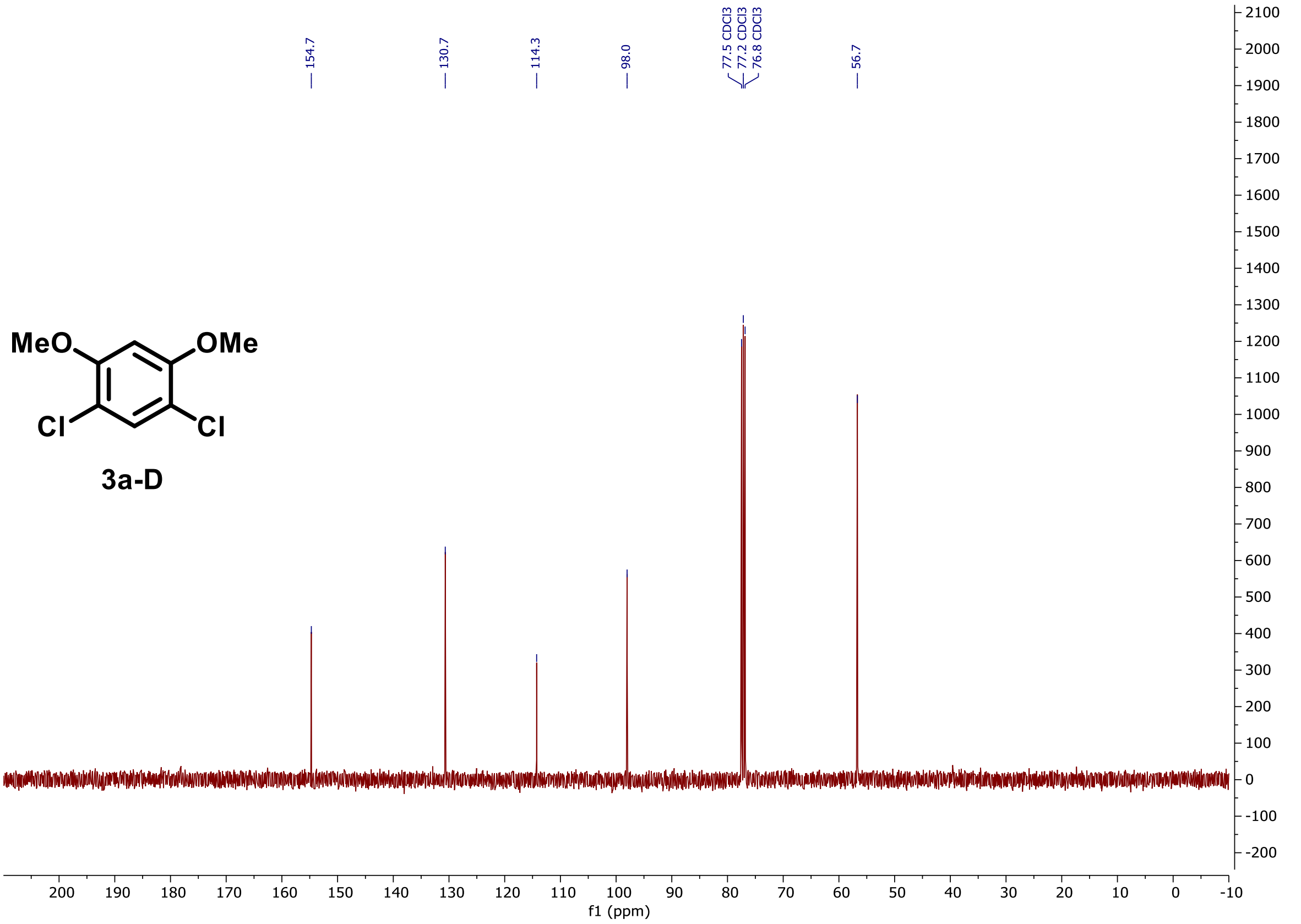

154.7
130.7
114.3
98.0
77.5 CDCl3
77.2 CDCl3
76.8 CDCl3
56.7
MeO
OMe
Cl
Cl
f1 (ppm)

3a-D

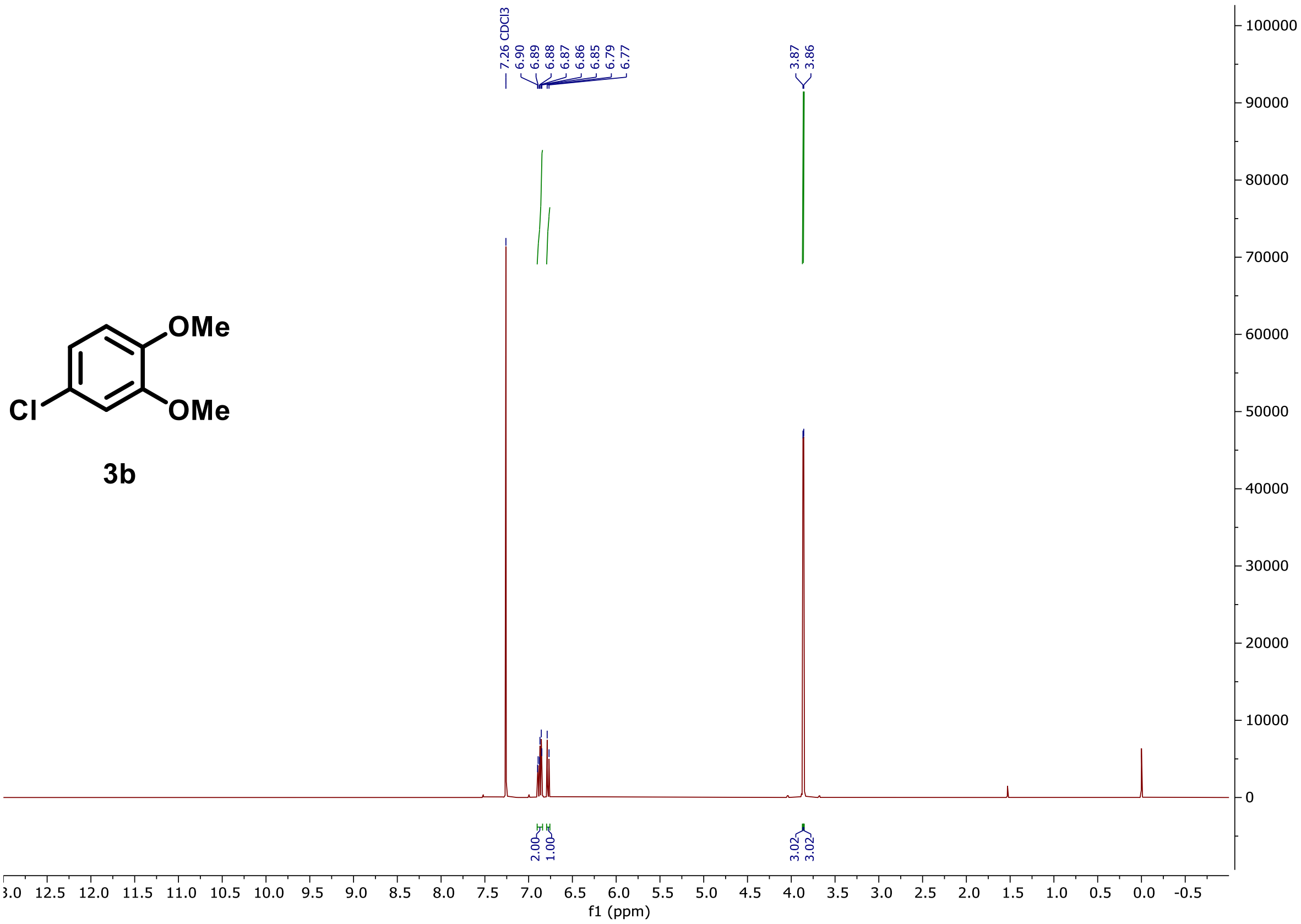
7.26 CDCl3
6.90
6.89
6.88
6.87
6.86
6.85
6.79
6.77
3.87
3.86
2.00
1.00
3.02
3.02
f1 (ppm)
OMe
OMe
Cl

3b

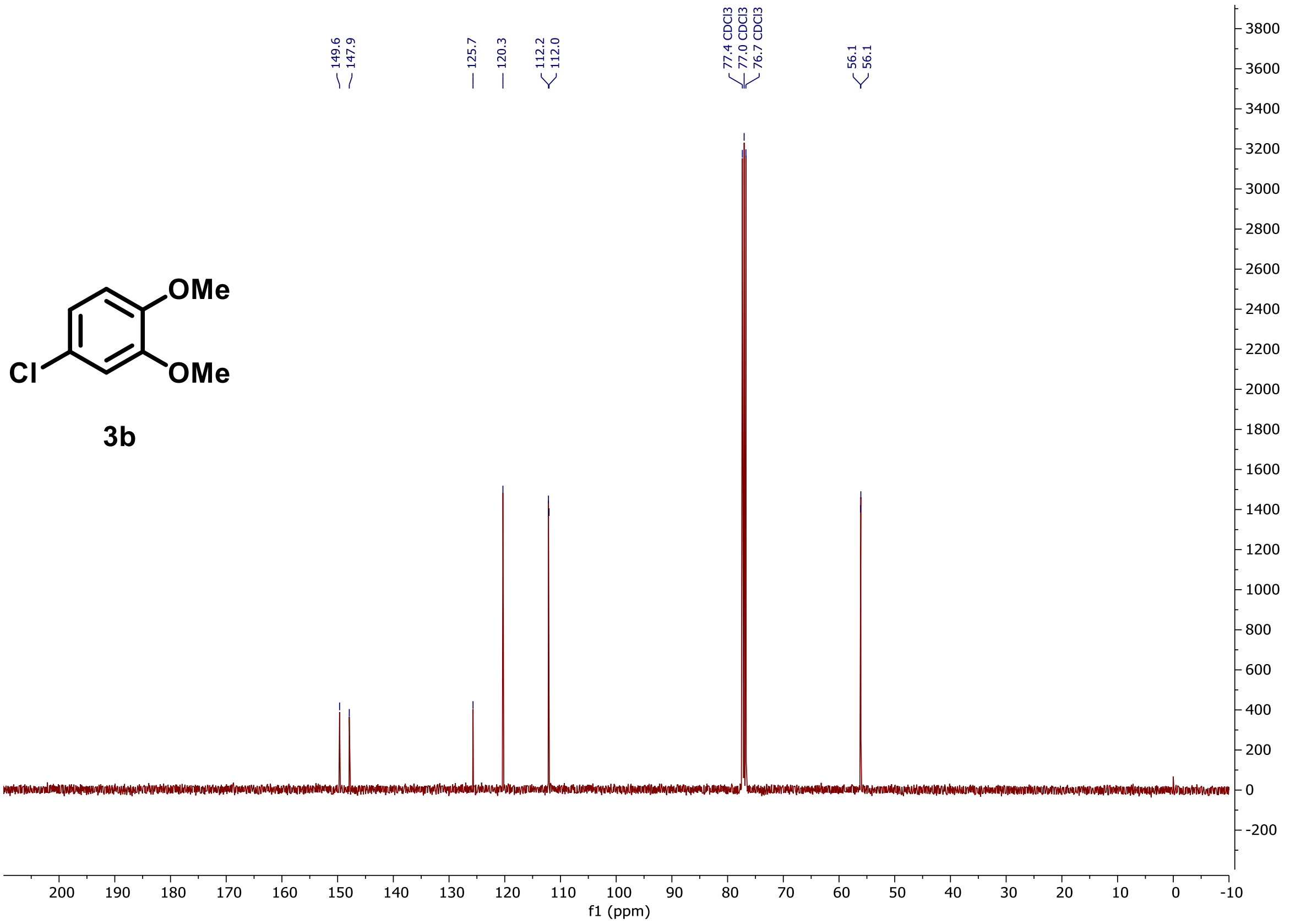
149.6
147.9
125.7
120.3
112.2
112.0
77.4 CDCl3
77.0 CDCl3
76.7 CDCl3
56.1
56.1
f1 (ppm)
OMe
OMe
Cl

3b

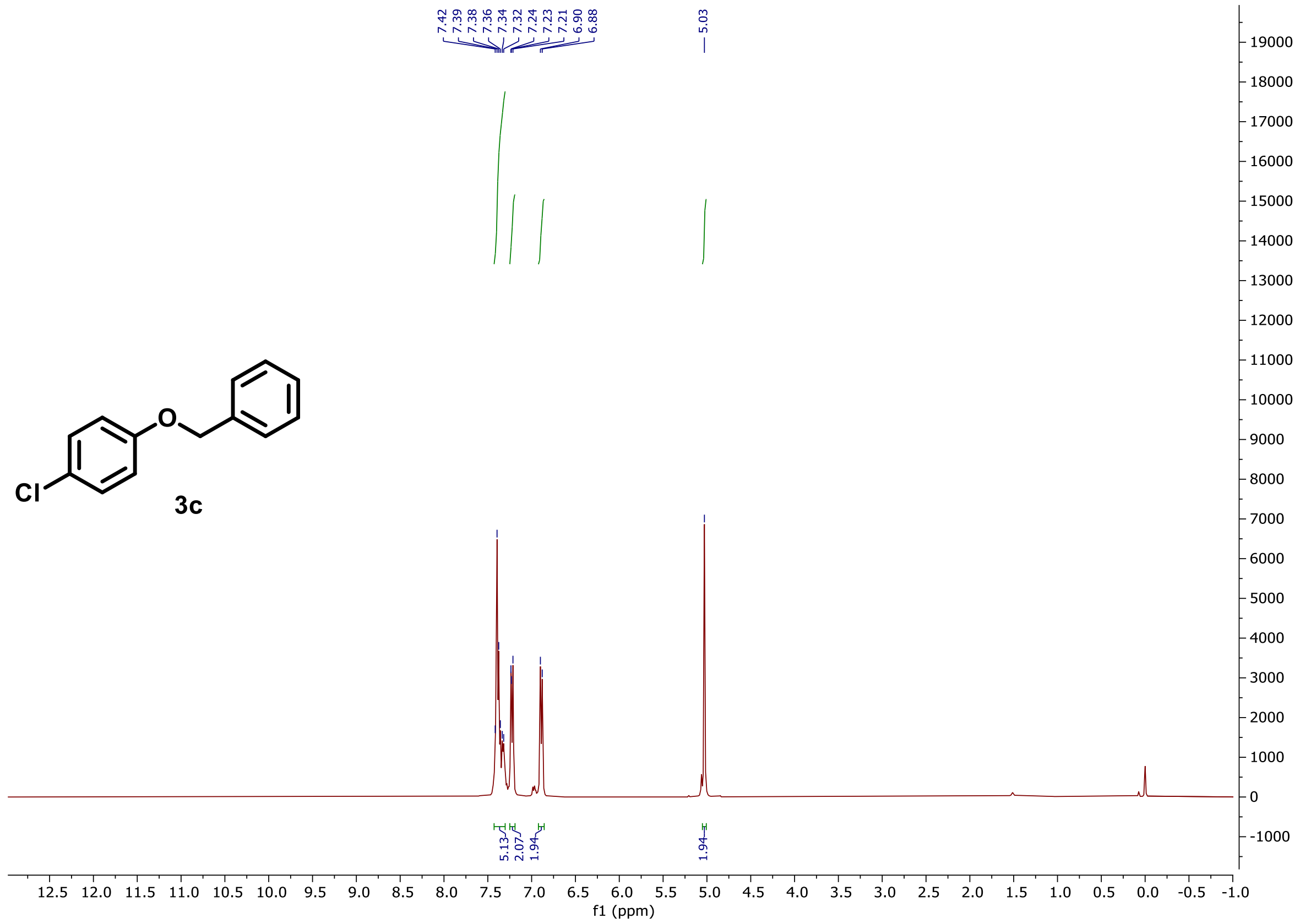
7.42
7.39
7.38
7.36
7.34
7.32
7.24
7.23
7.21
6.90
6.88
5.03
Cl
O
3c
5.13
2.07
1.94
1.94
f1 (ppm)

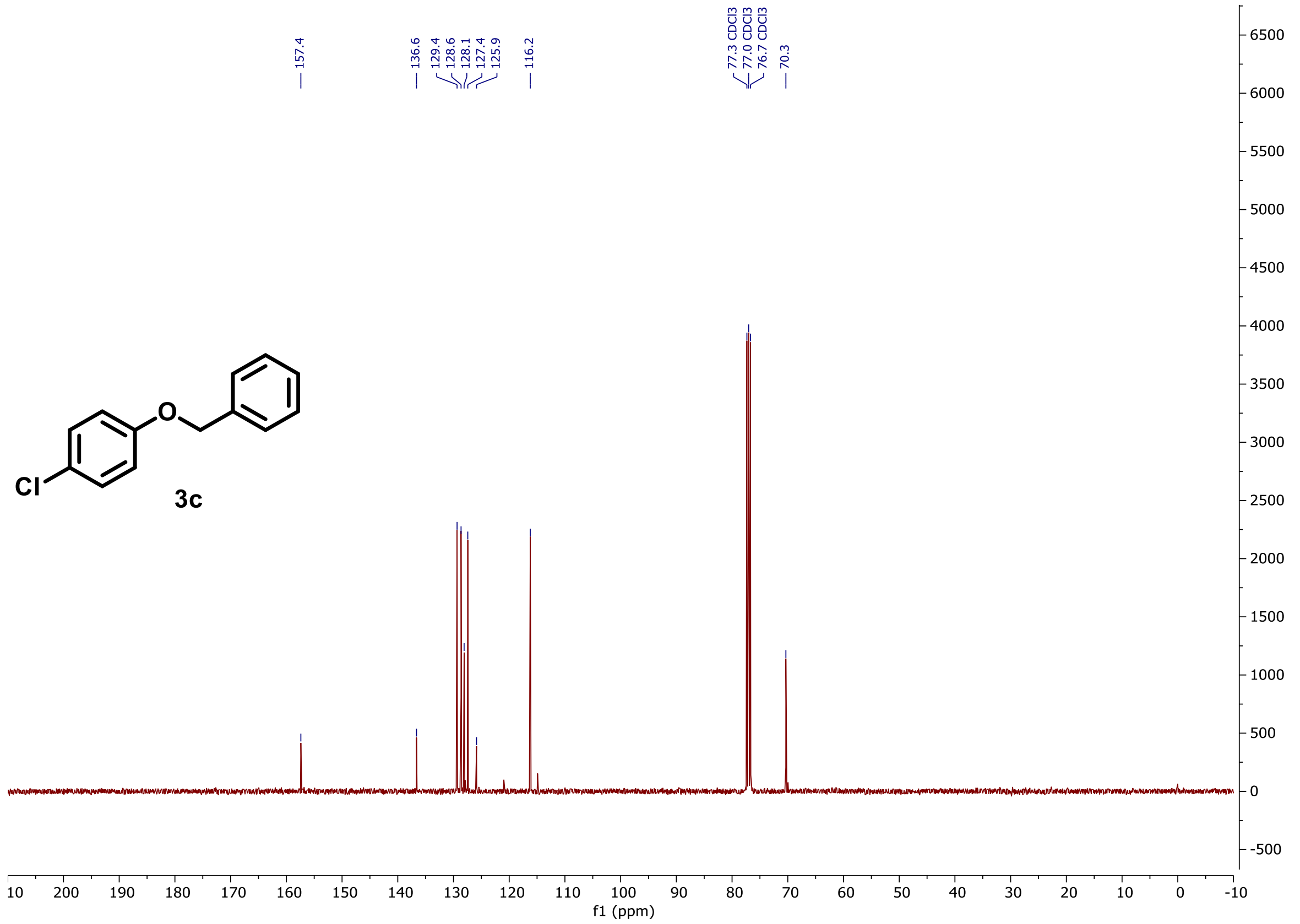
157.4
136.6
129.4
128.6
128.1
127.4
125.9
116.2
77.3 CDCl3
77.0 CDCl3
76.7 CDCl3
70.3
Cl
O
3c
f1 (ppm)

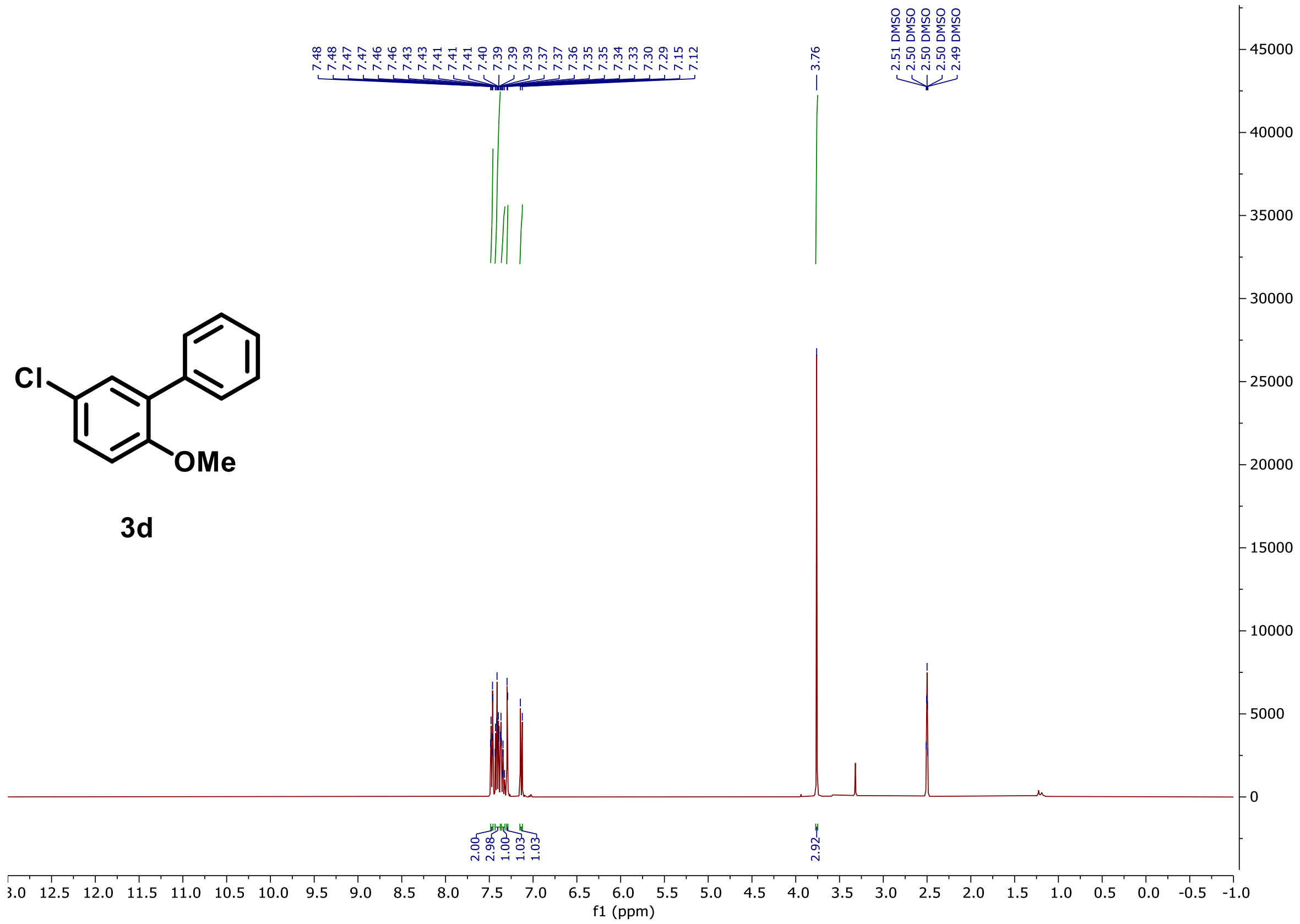
7.48 7.48 7.47 7.47 7.46 7.43 7.43 7.41 7.41 7.41 7.40 7.39 7.39 7.39 7.39 7.37 7.37 7.36 7.35 7.35 7.34 7.33 7.30 7.29 7.15 7.12
3.76
2.51 DMSO 2.50 DMSO 2.50 DMSO 2.50 DMSO 2.49 DMSO
Cl
OMe
3d
2.00 2.98 1.00 1.03 1.03
2.92
f1 (ppm)

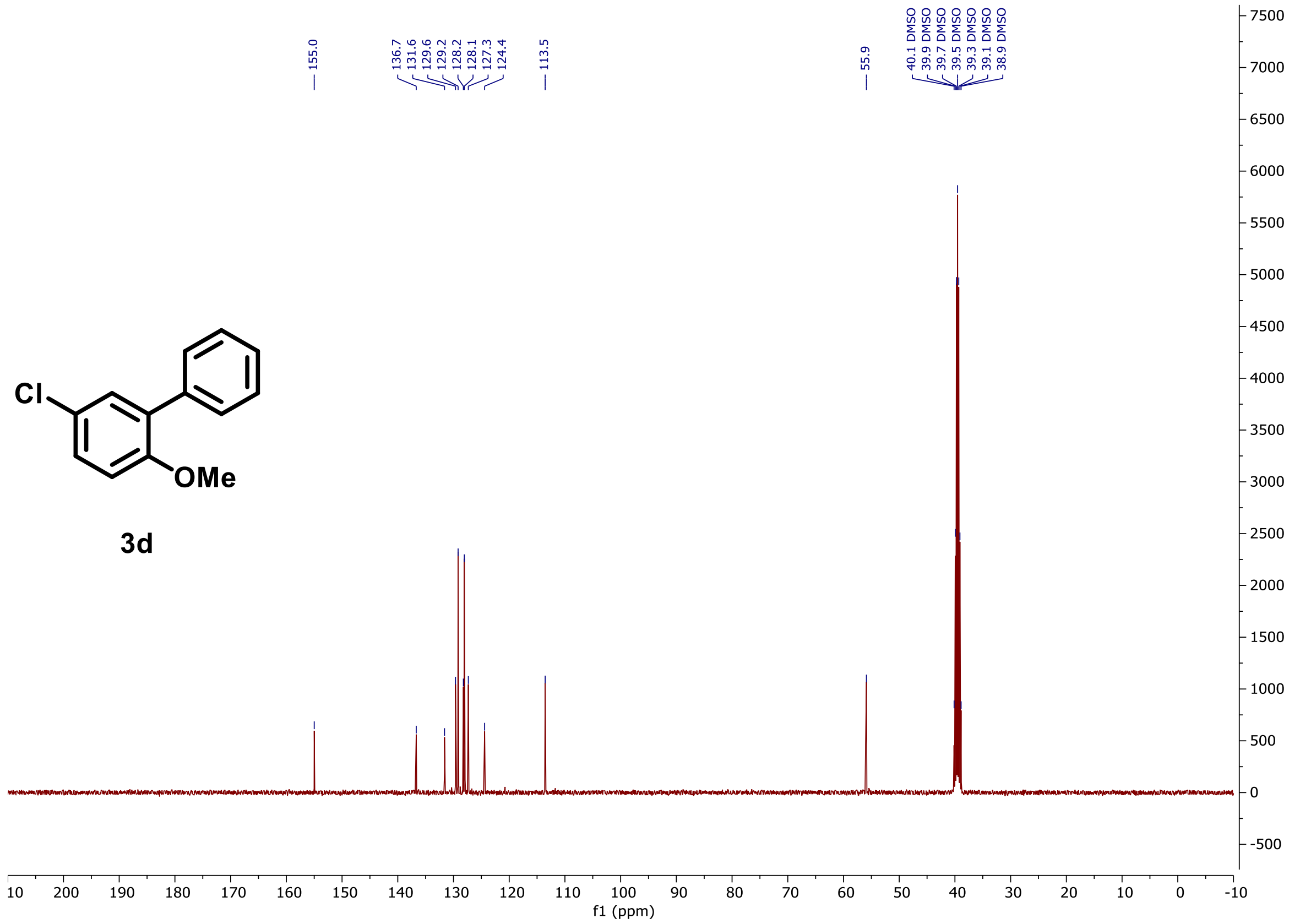
155.0
136.7 131.6 129.6 129.2 128.2 128.1 127.3 124.4
113.5
55.9
40.1 DMSO 39.9 DMSO 39.7 DMSO 39.5 DMSO 39.3 DMSO 39.1 DMSO 38.9 DMSO
Cl
OMe
3d
f1 (ppm)

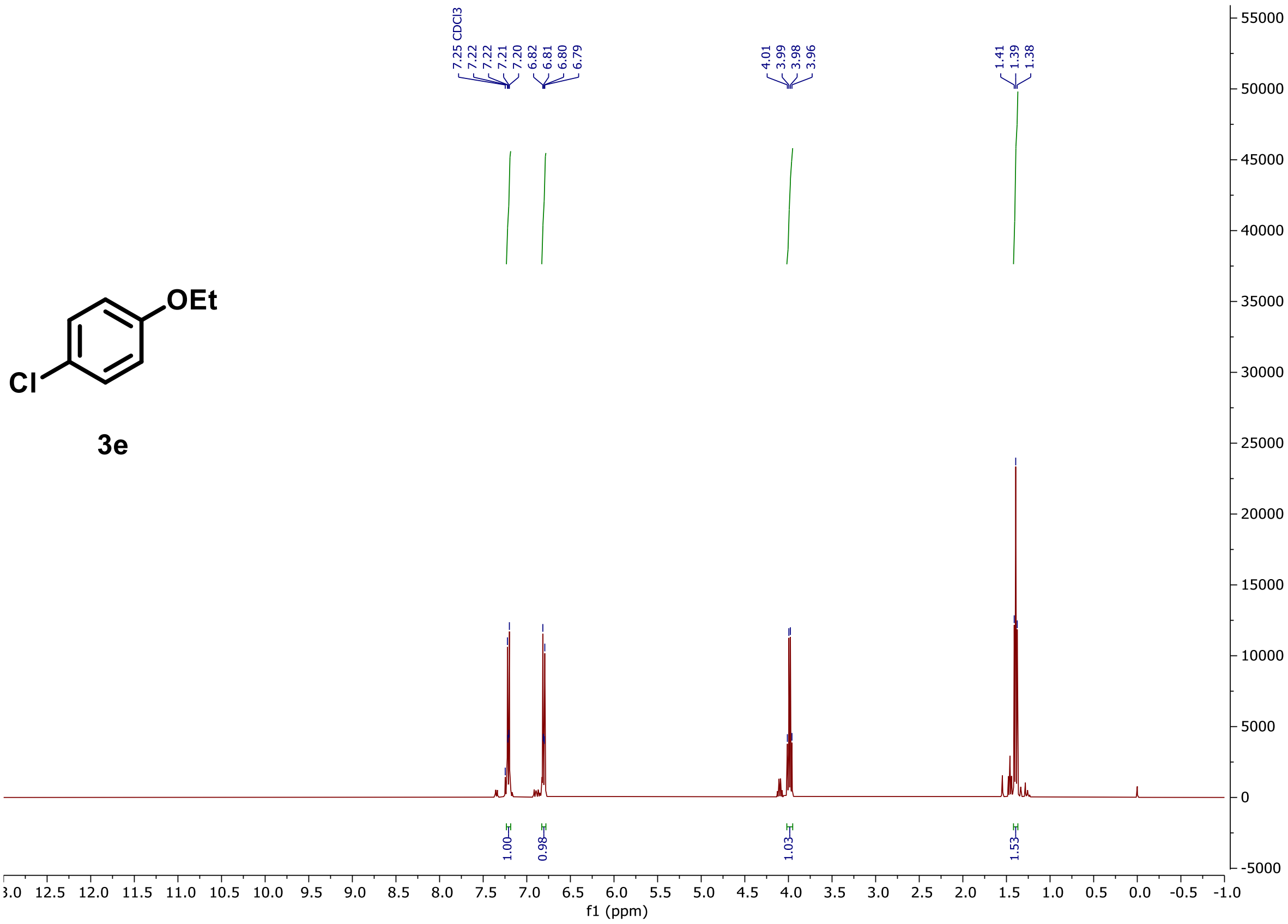

OEt
Cl
3e
f1 (ppm)

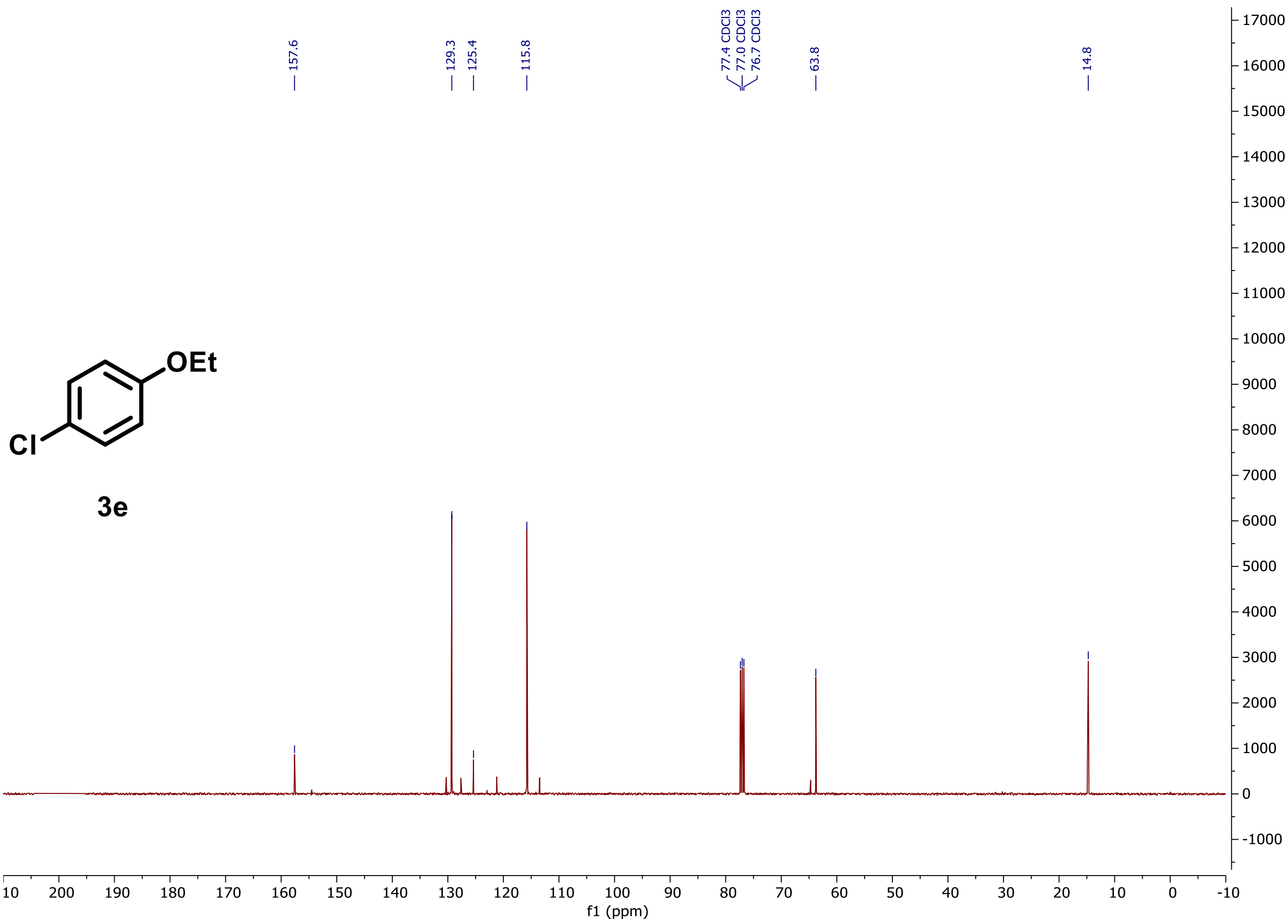

OEt
Cl
3e
f1 (ppm)

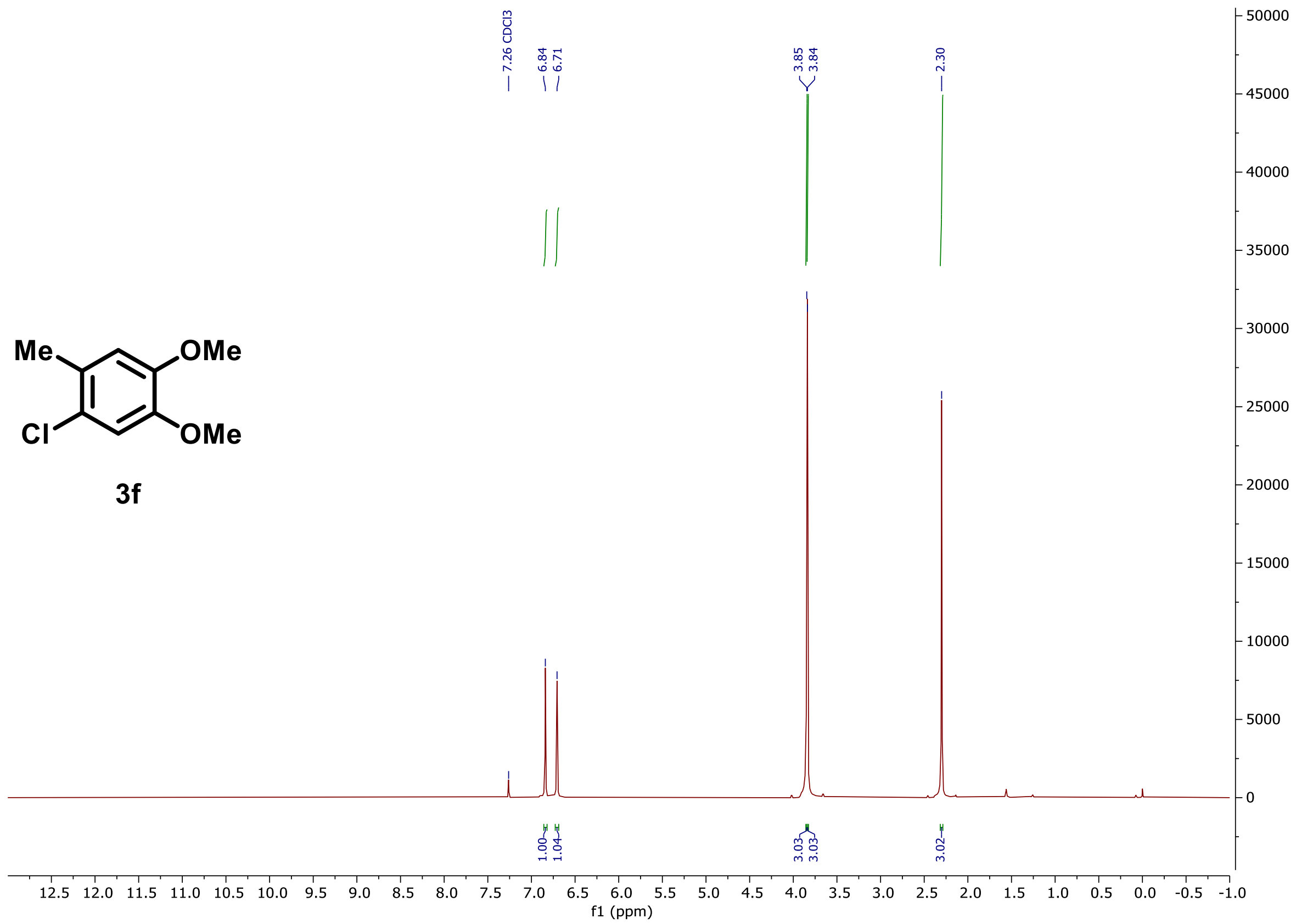
Me
OMe
Cl
OMe
3f
7.26 CDCl3
6.84
6.71
3.85
3.84
2.30
1.00
1.04
3.03
3.03
3.02
f1 (ppm)

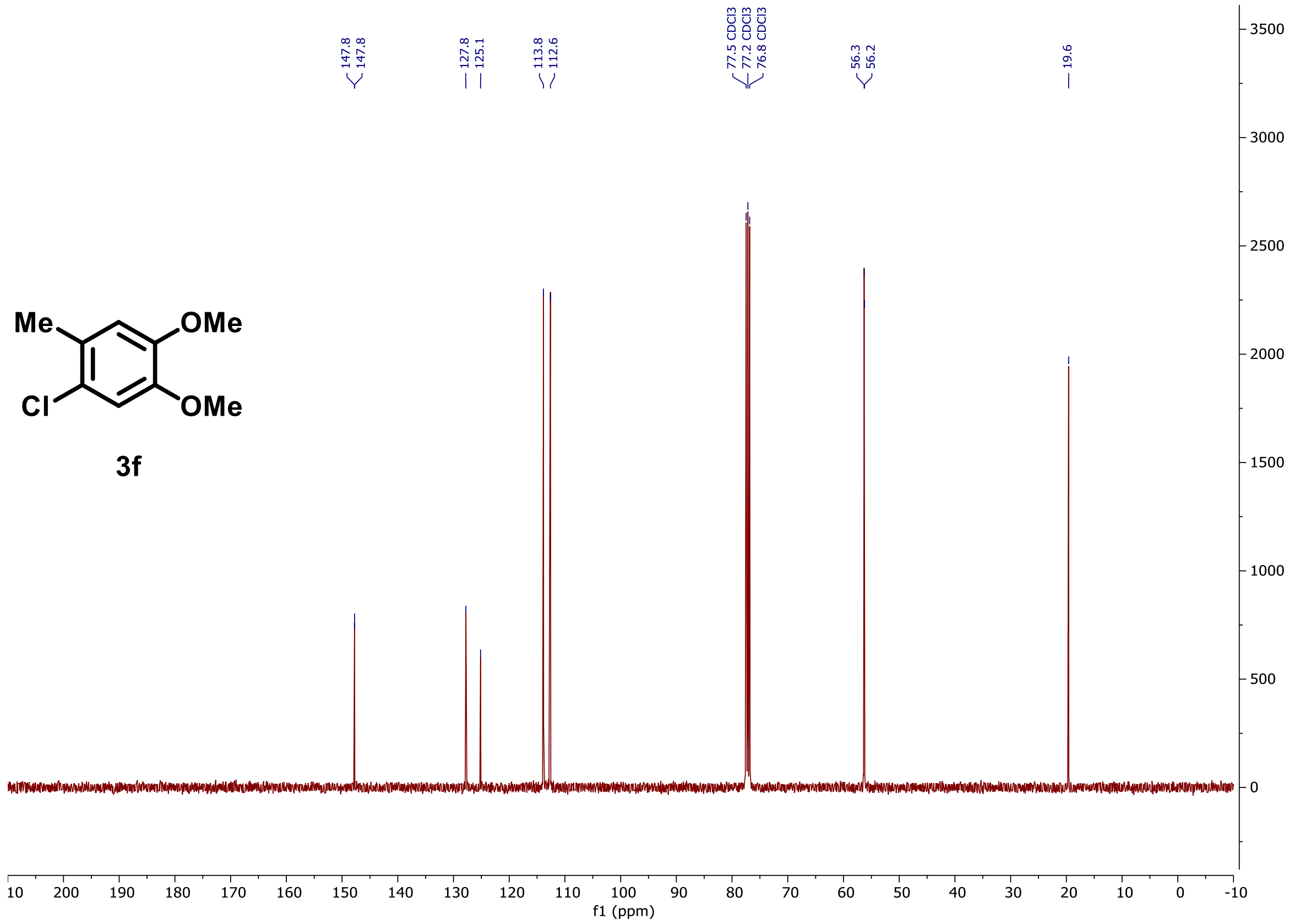
Me
OMe
Cl
OMe
3f
147.8
147.8
127.8
125.1
113.8
112.6
77.5 CDCl3
77.2 CDCl3
76.8 CDCl3
56.3
56.2
19.6
f1 (ppm)

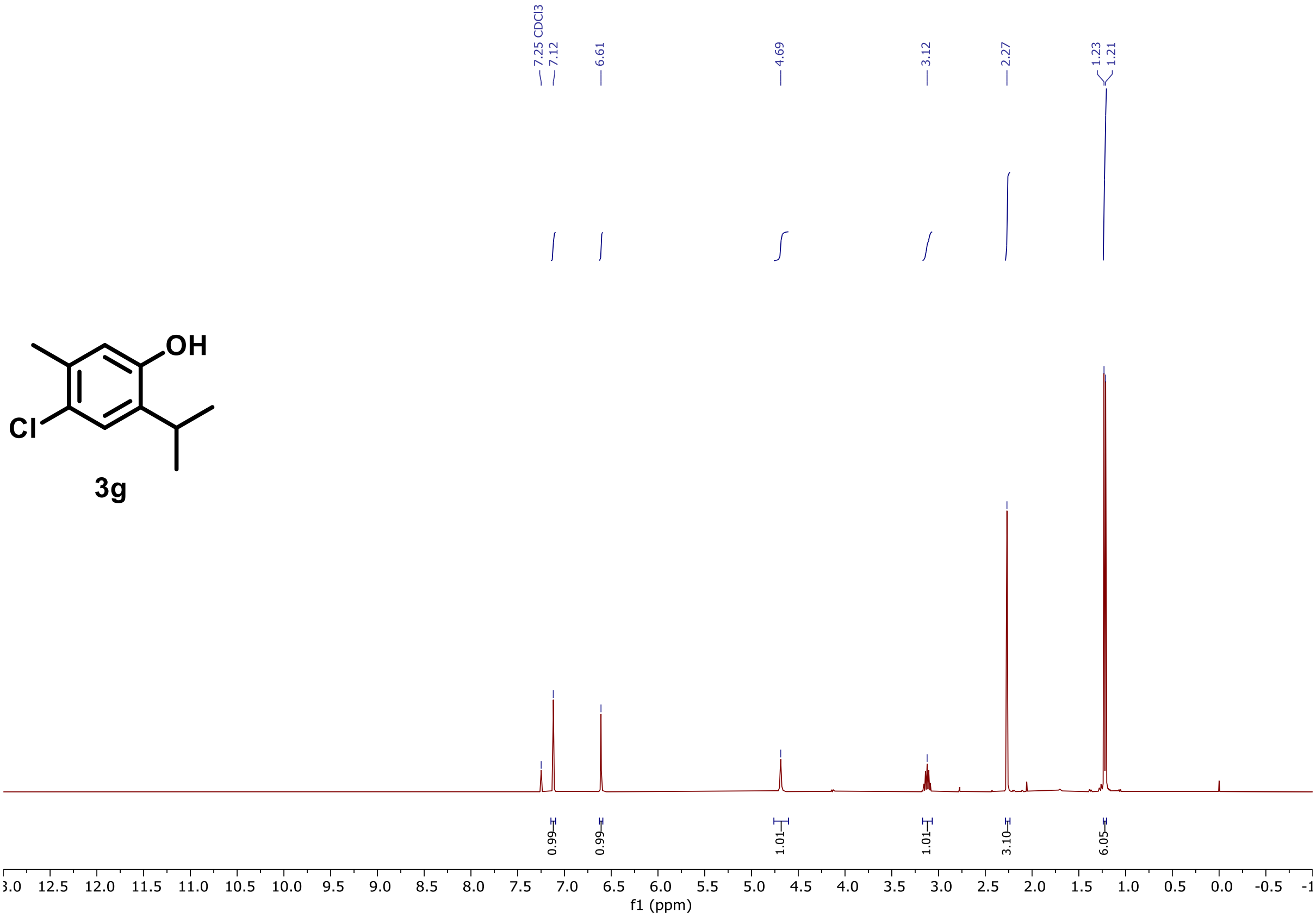
7.25 CDCl3
7.12
6.61
4.69
3.12
2.27
1.23
1.21
OH
Cl
3g
0.99
0.99
1.01
1.01
3.10
6.05
f1 (ppm)

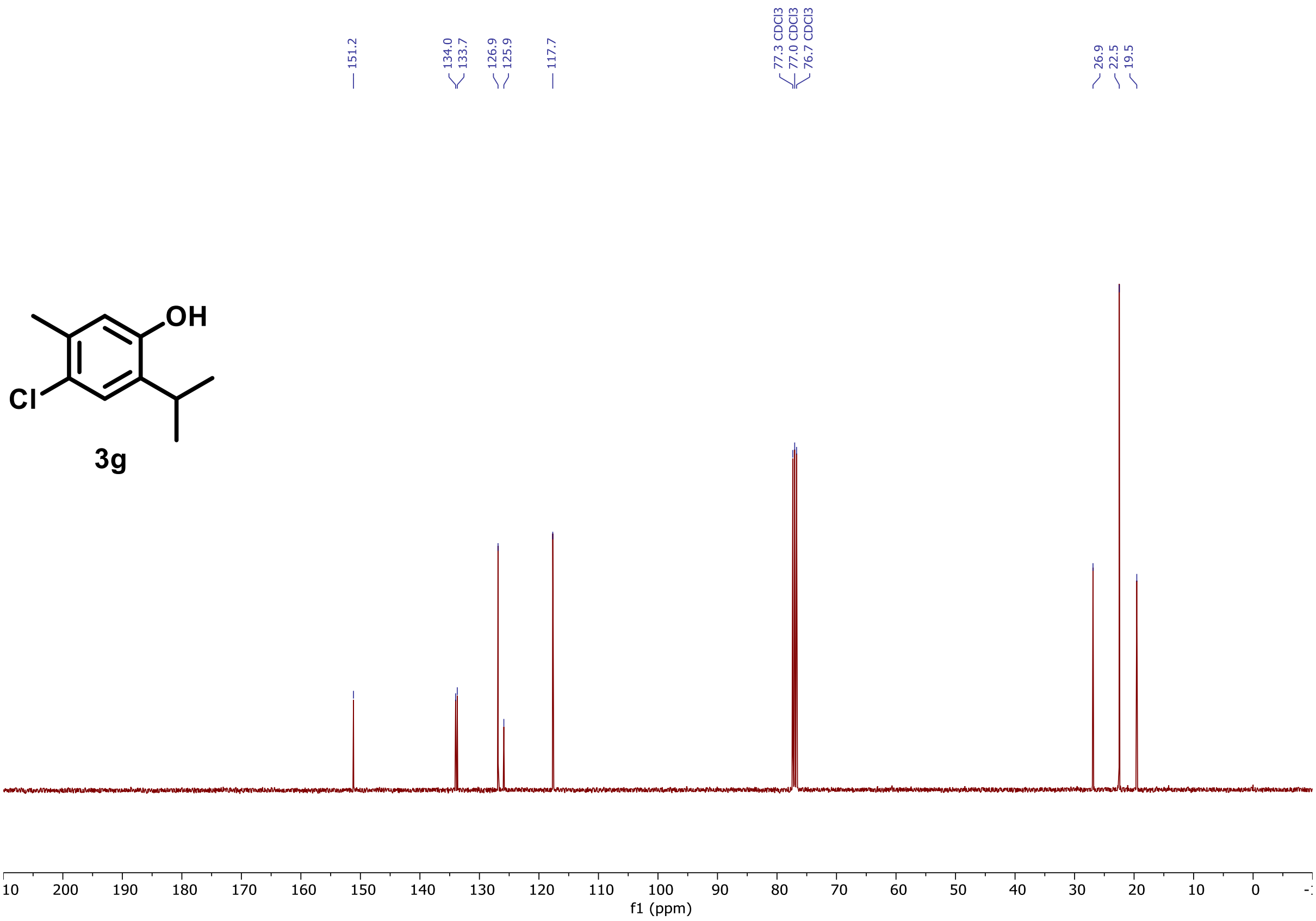
151.2
134.0
133.7
126.9
125.9
117.7
77.3 CDCl3
77.0 CDCl3
76.7 CDCl3
26.9
22.5
19.5
OH
Cl
3g
f1 (ppm)

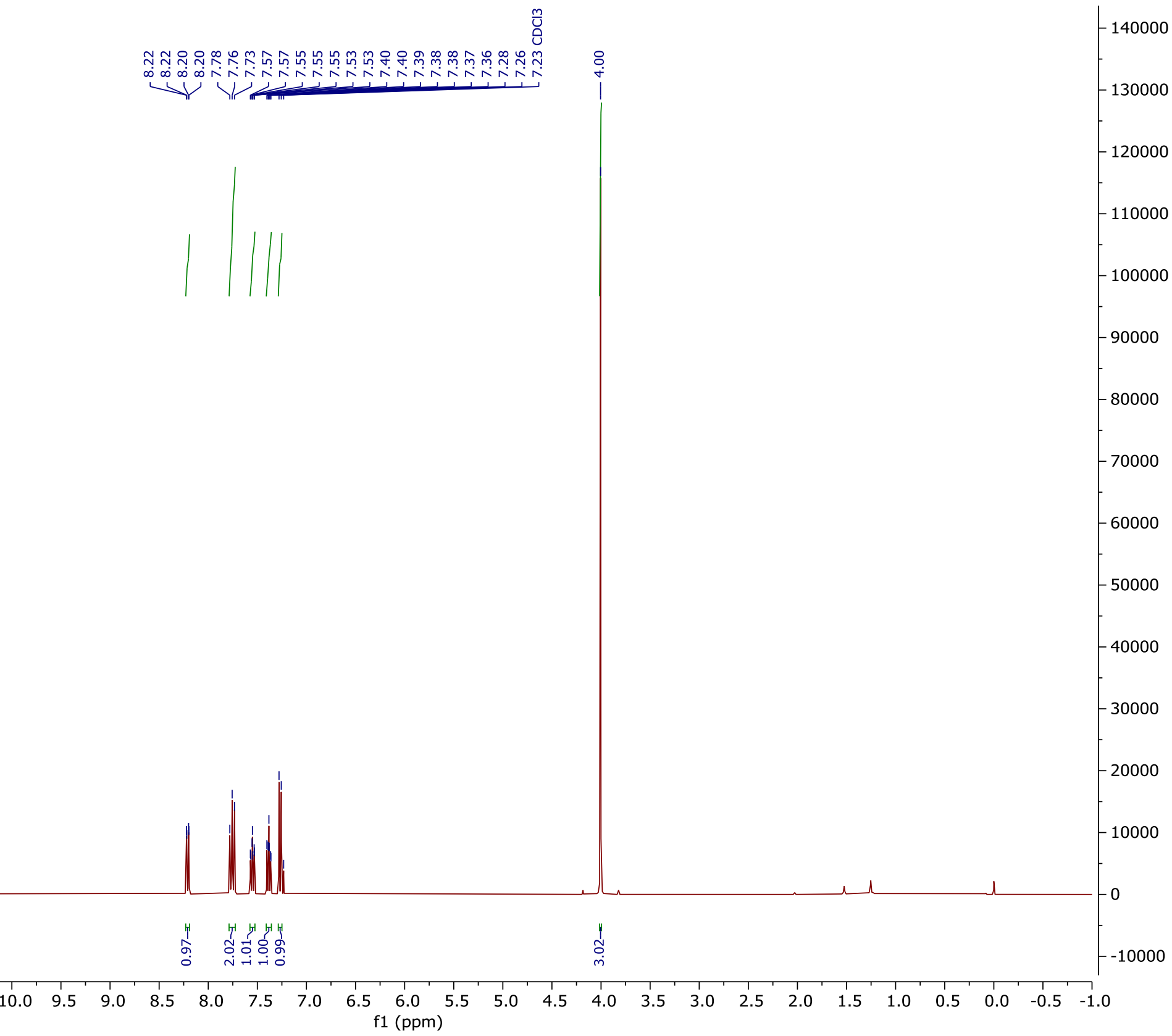

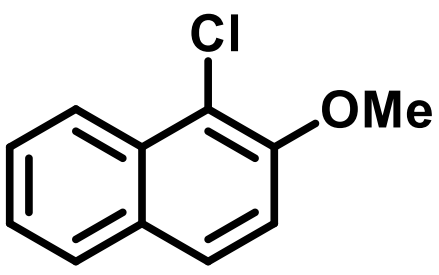

3h

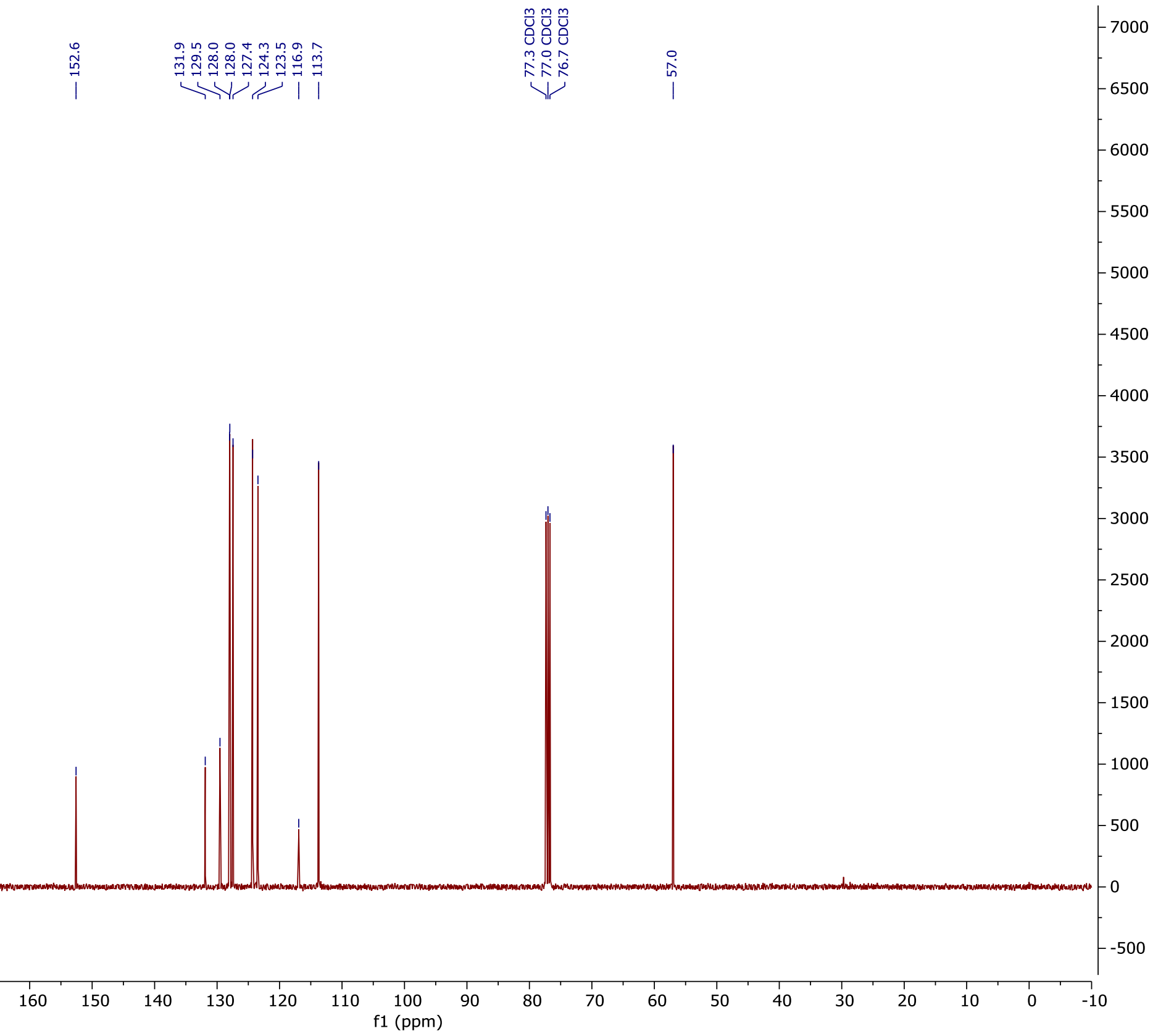

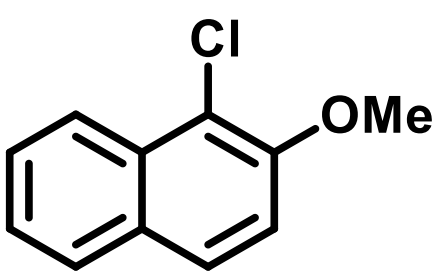

3h

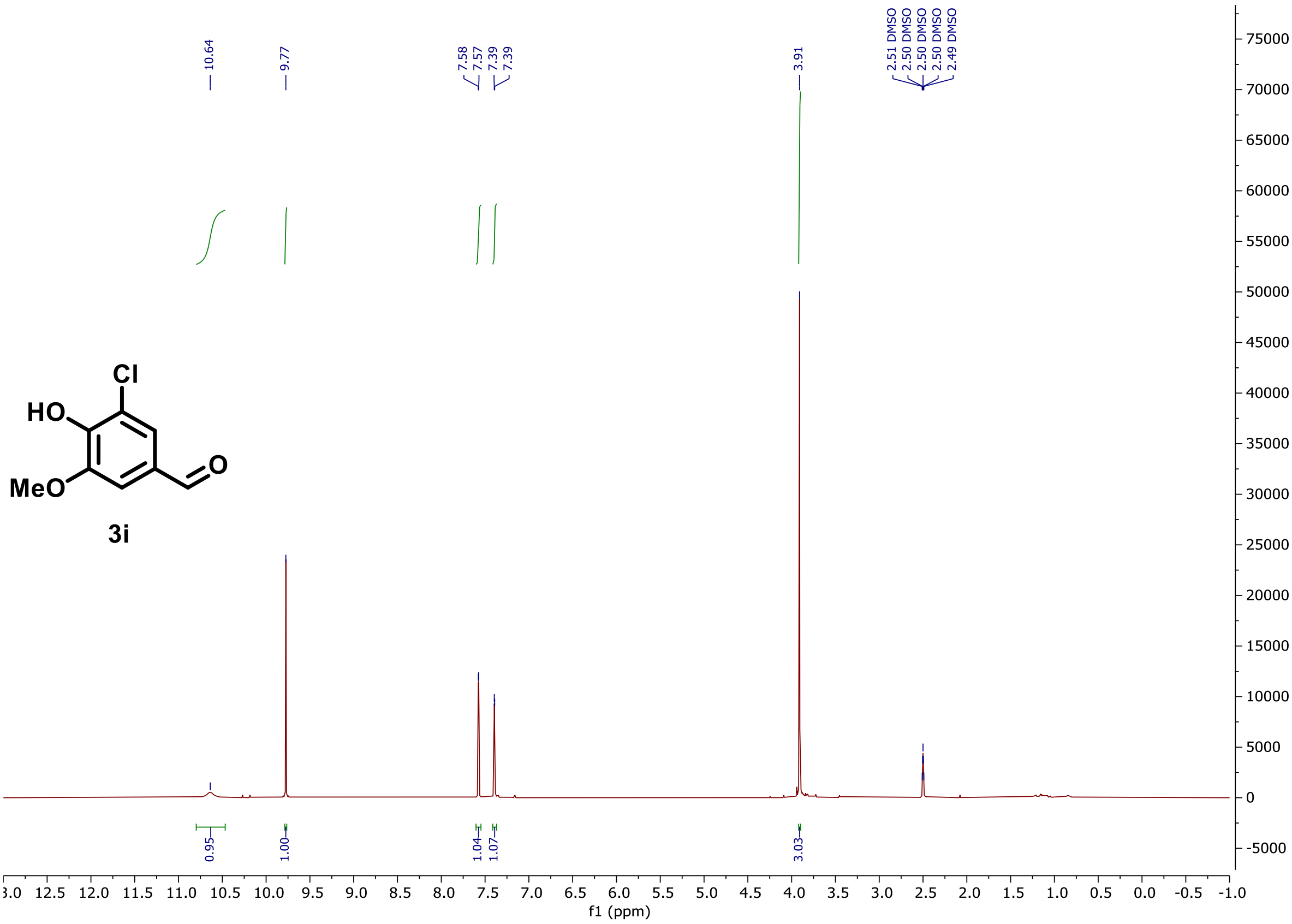
10.64
9.77
7.58
7.57
7.39
7.39
3.91
2.51 DMSO
2.50 DMSO
2.50 DMSO
2.50 DMSO
2.49 DMSO
Cl
HO
MeO
O
3i
0.95
1.00
1.04
1.07
3.03
f1 (ppm)

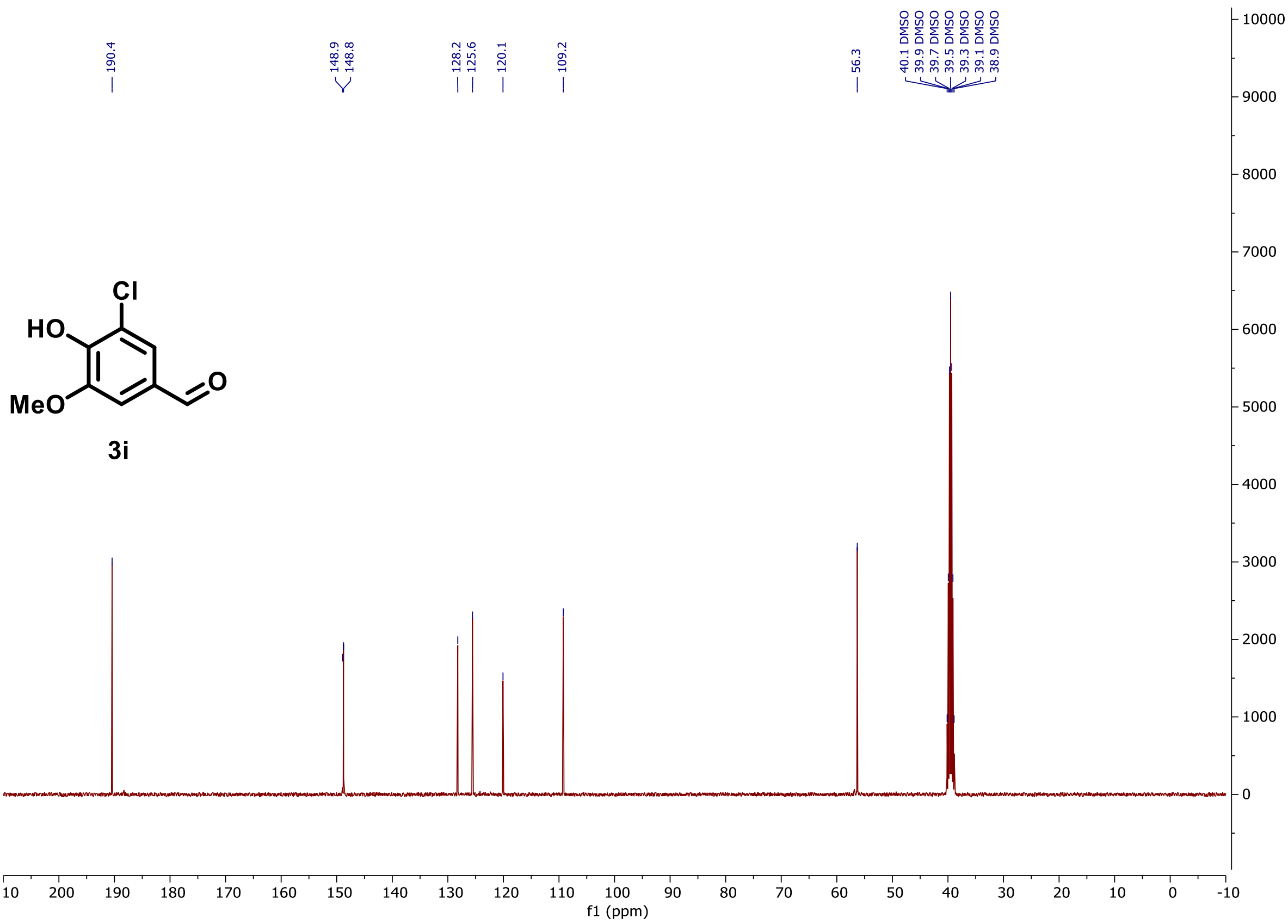
190.4
148.9
148.8
128.2
125.6
120.1
109.2
56.3
40.1 DMSO
39.9 DMSO
39.7 DMSO
39.5 DMSO
39.3 DMSO
39.1 DMSO
38.9 DMSO
Cl
HO
MeO
O
3i
f1 (ppm)

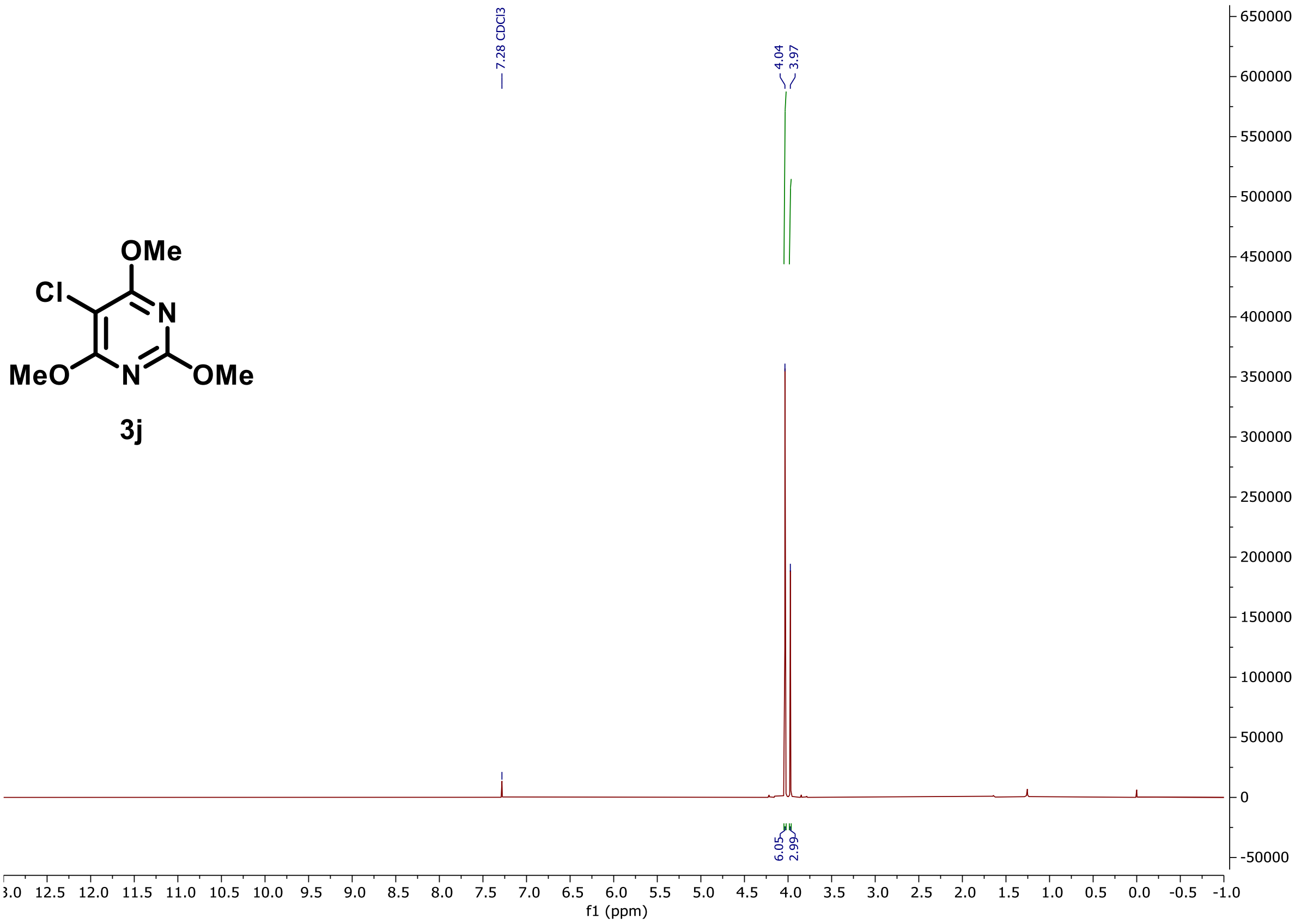

7.28 CDCl3
4.04
3.97
OMe
Cl
N
MeO
N
OMe
3j
6.05
2.99
f1 (ppm)

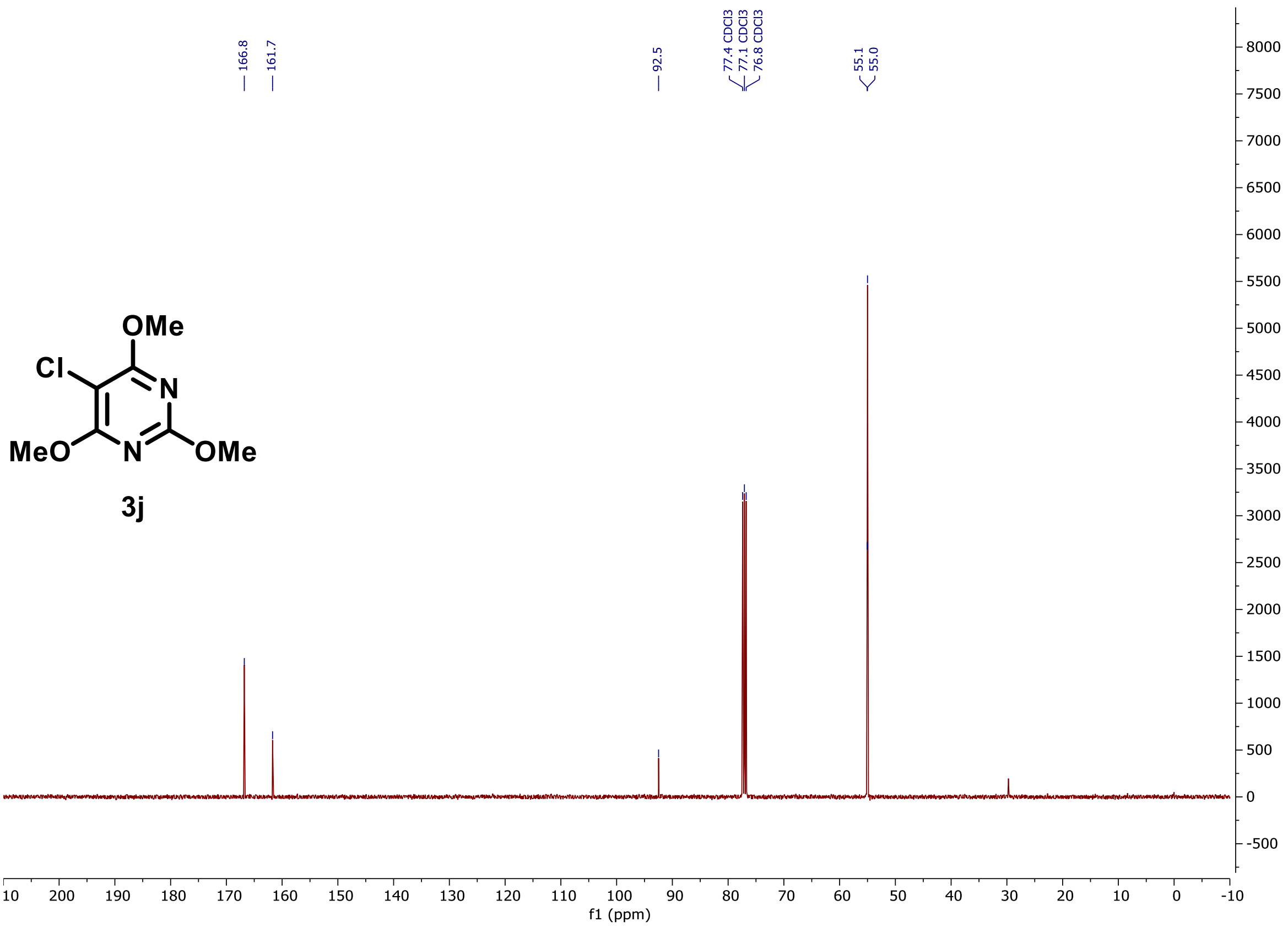

166.8
161.7
92.5
77.4 CDCl3
77.1 CDCl3
76.8 CDCl3
55.1
55.0
OMe
Cl
N
MeO
N
OMe
3j
f1 (ppm)

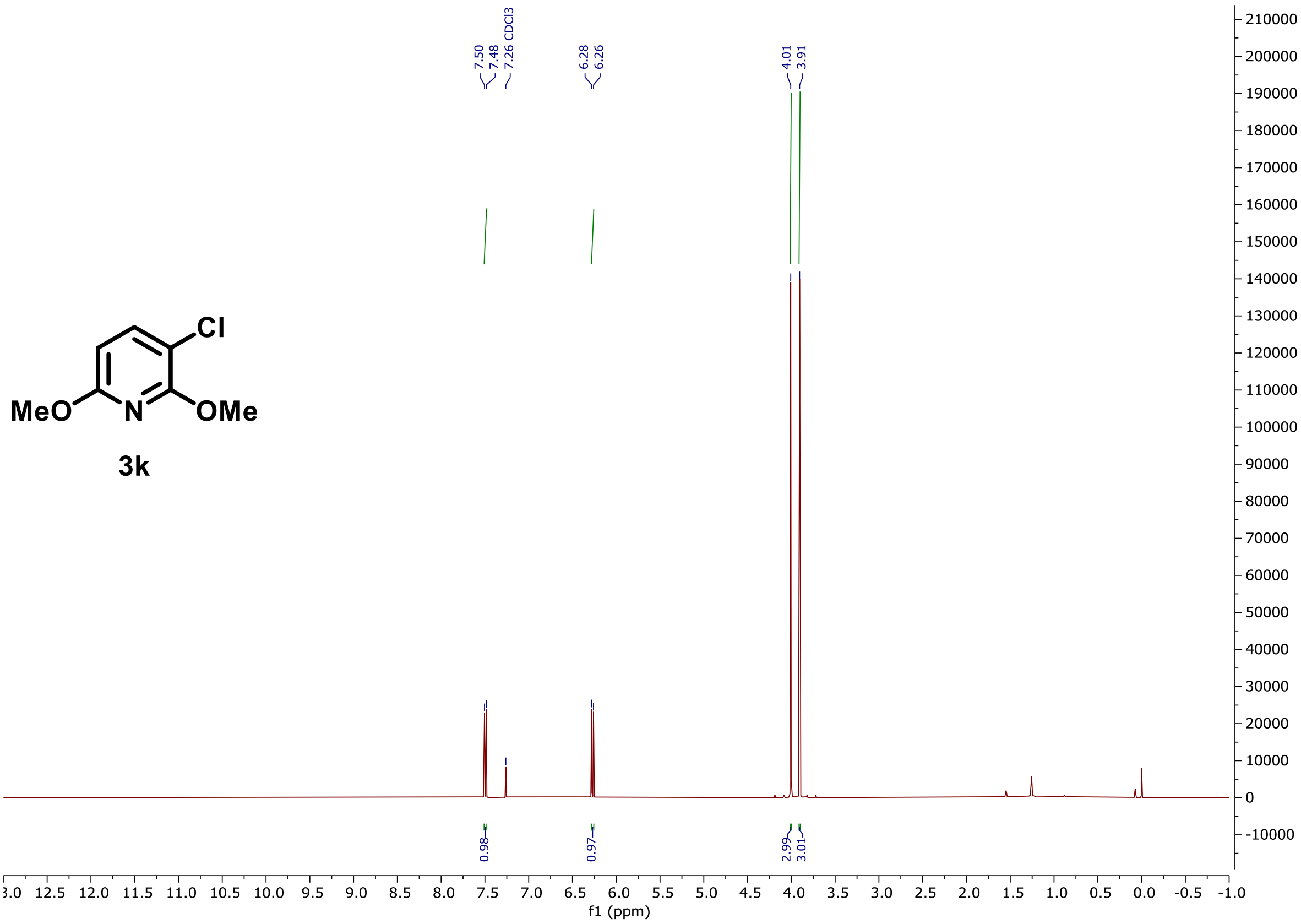

7.50
7.48
7.26 CDCl3
6.28
6.26
4.01
3.91
Cl
MeO
N
OMe
3k
0.98
0.97
2.99
3.01
f1 (ppm)

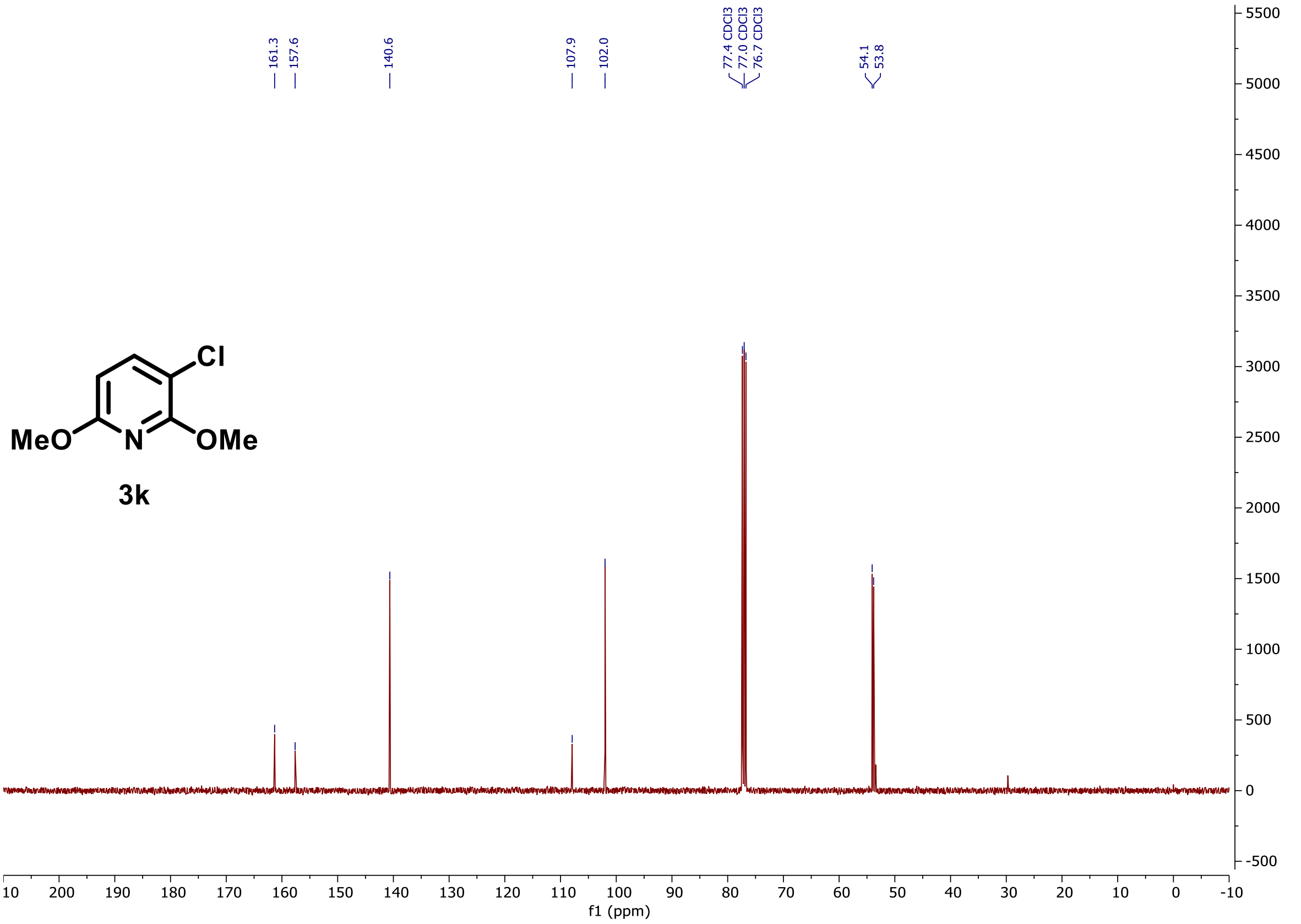

161.3
157.6
140.6
107.9
102.0
77.4 CDCl3
77.0 CDCl3
76.7 CDCl3
54.1
53.8
Cl
MeO
N
OMe
3k
f1 (ppm)

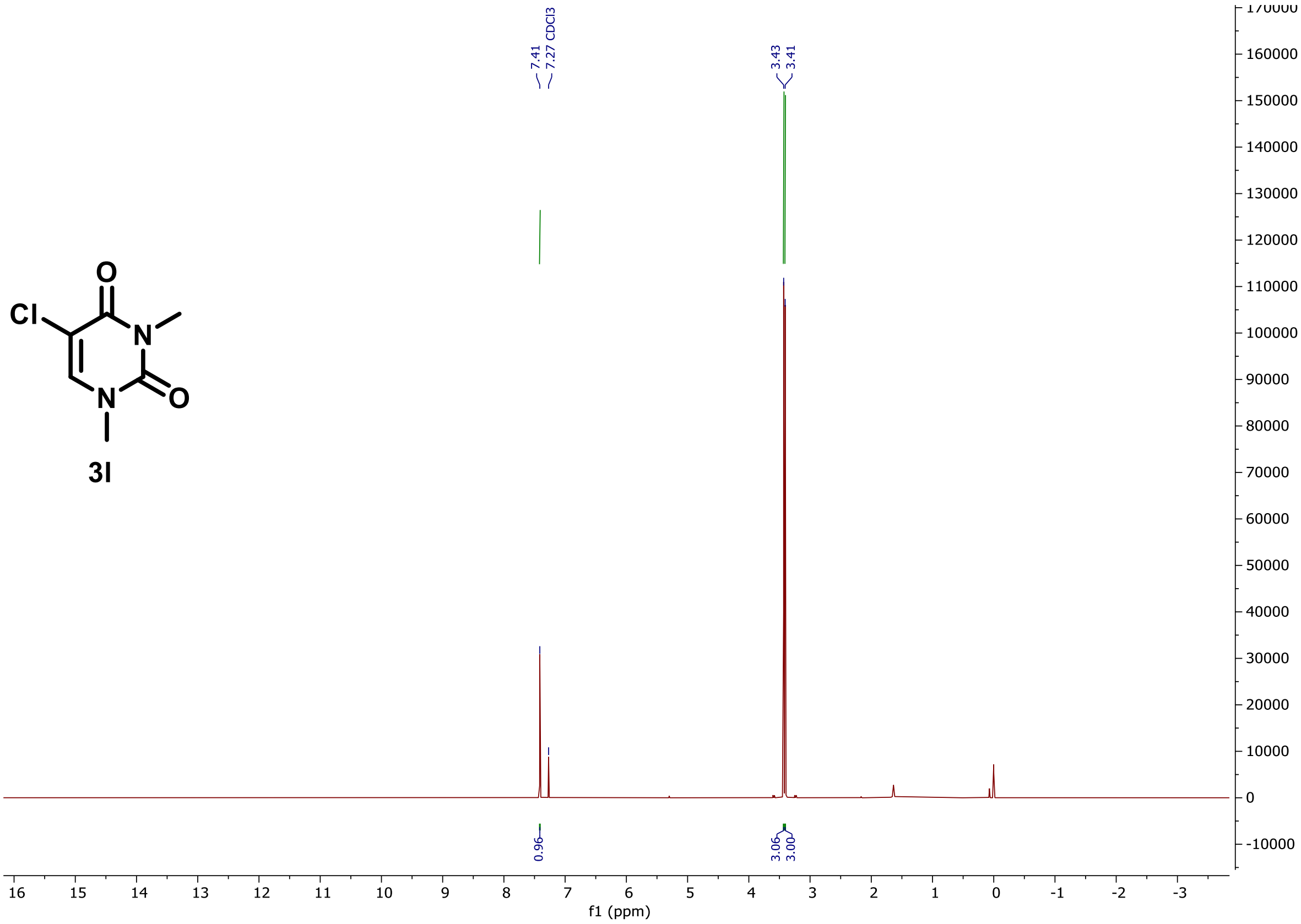
3l
7.41
7.27 CDCl3
3.43
3.41
0.96
3.06
3.00
f1 (ppm)

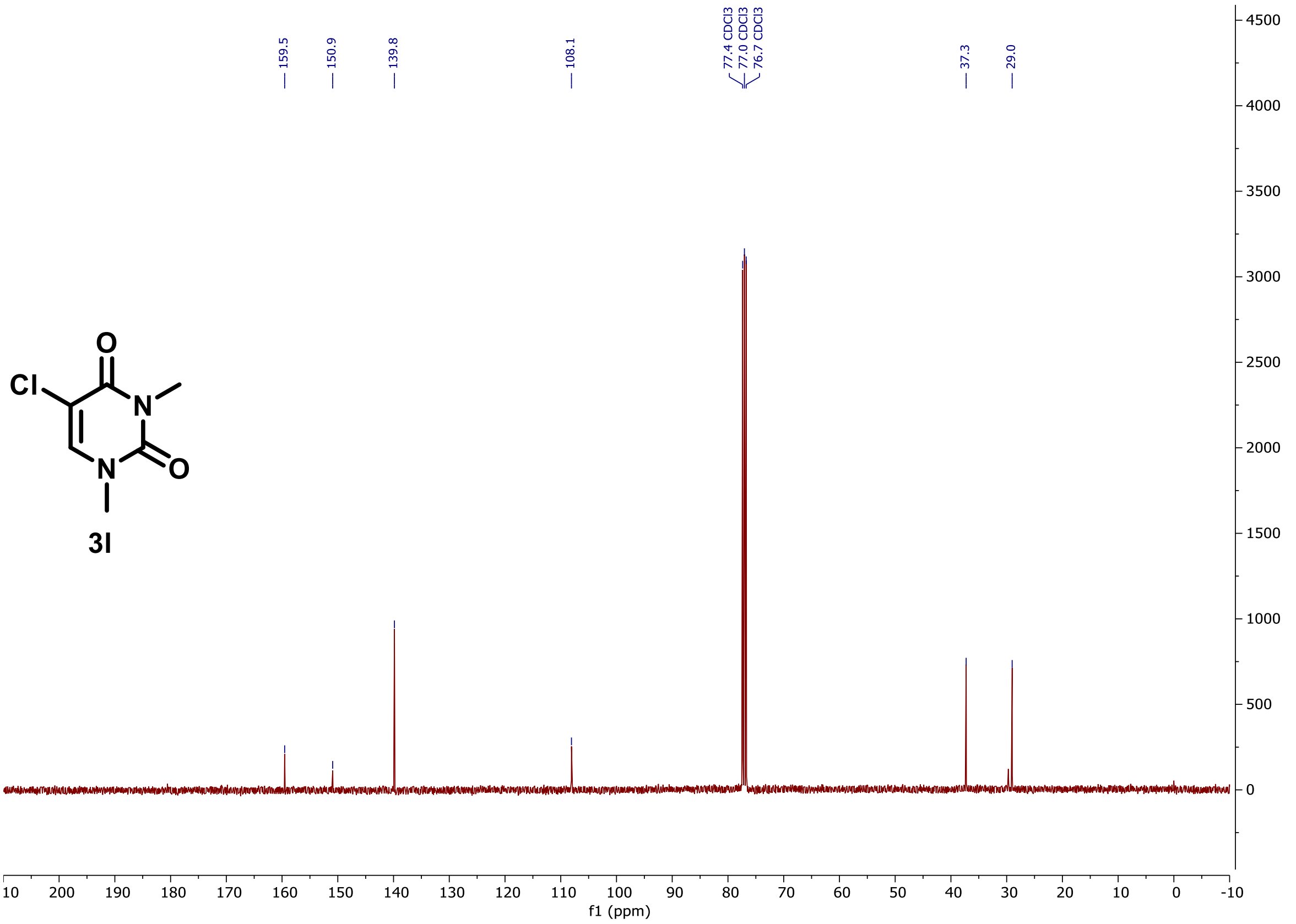
3l
159.5
150.9
139.8
108.1
77.4 CDCl3
77.0 CDCl3
76.7 CDCl3
37.3
29.0
f1 (ppm)

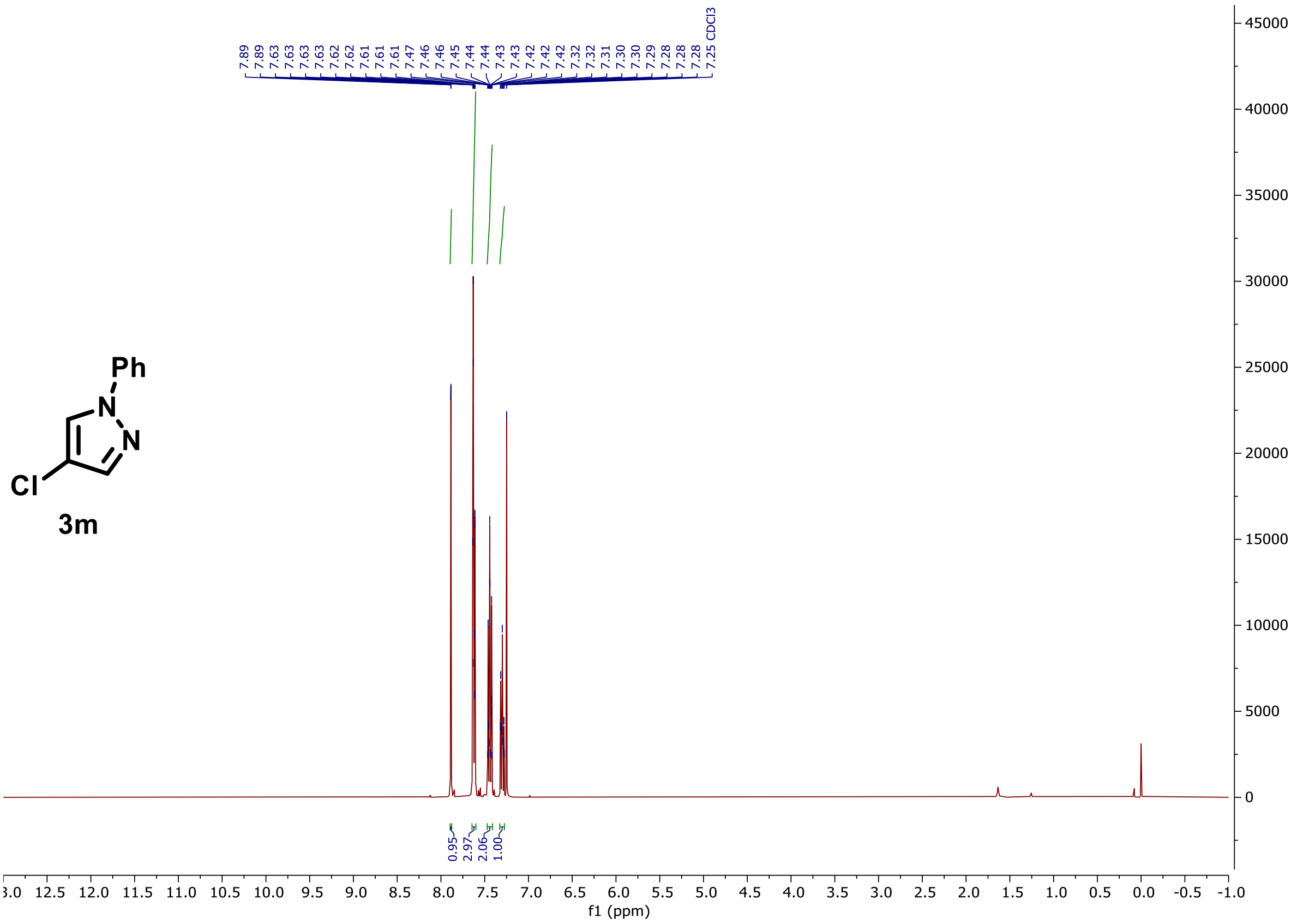
7.89
7.89
7.63
7.63
7.63
7.63
7.62
7.62
7.61
7.61
7.61
7.47
7.46
7.45
7.44
7.44
7.43
7.43
7.42
7.42
7.42
7.32
7.32
7.31
7.30
7.30
7.29
7.28
7.28
7.28
7.25 CDCl3
Ph
N
N
Cl
3m
0.95
2.97
2.06
1.00
f1 (ppm)

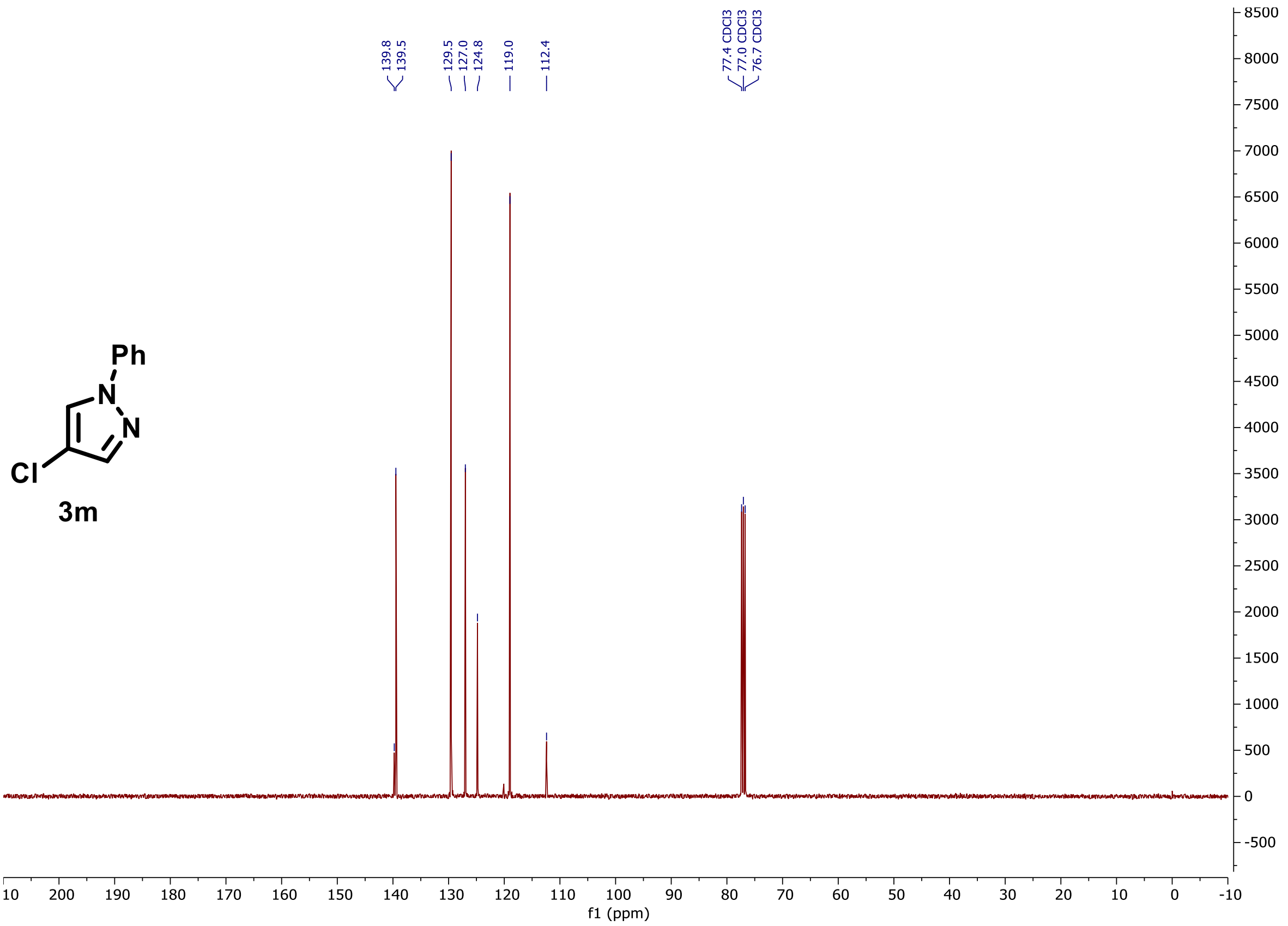
139.8
139.5
129.5
127.0
124.8
119.0
112.4
77.4 CDCl3
77.0 CDCl3
76.7 CDCl3
Ph
N
N
Cl
3m
f1 (ppm)

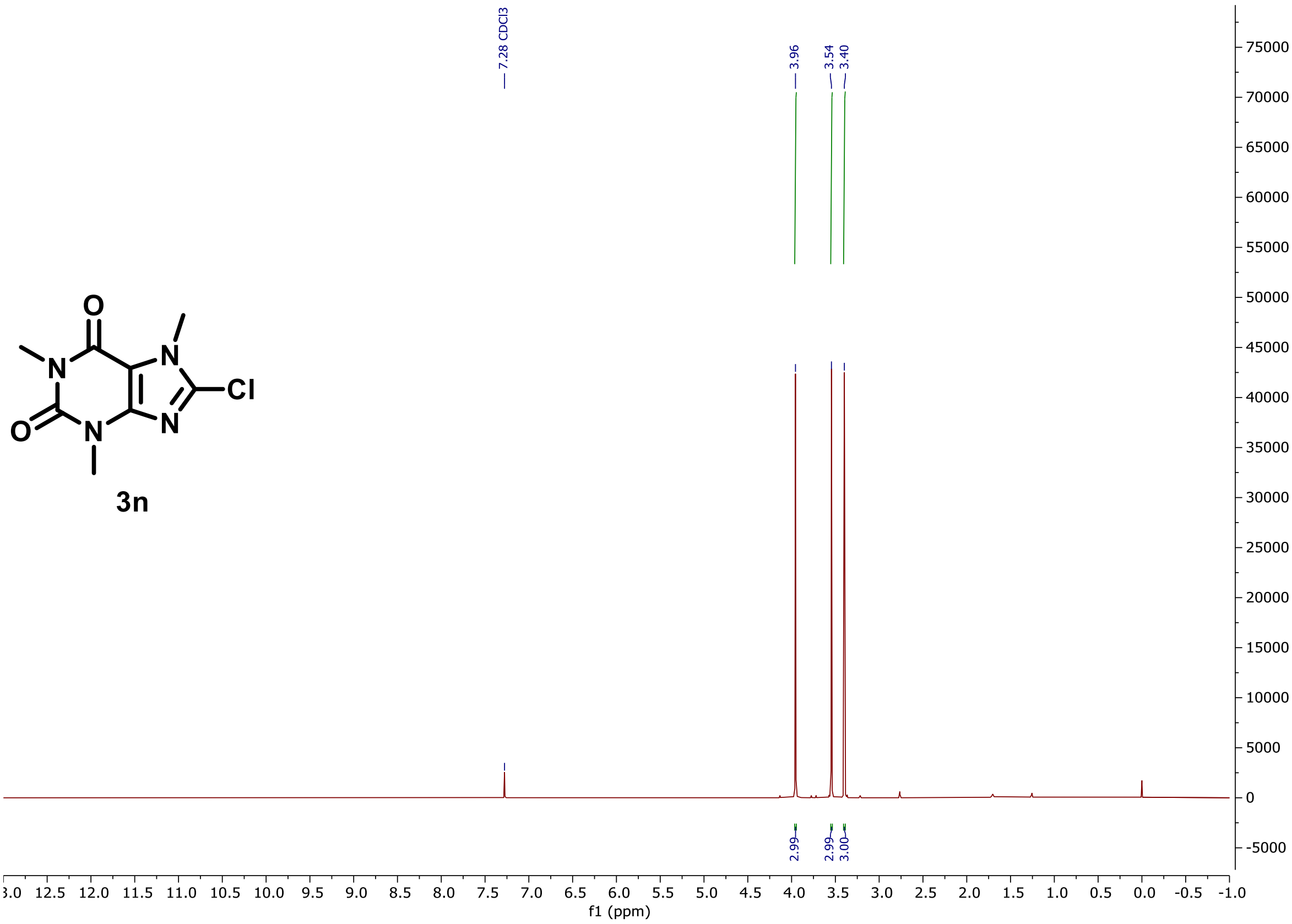

3n
7.28 CDCl3
3.96
3.54
3.40
2.99
2.99
3.00
f1 (ppm)

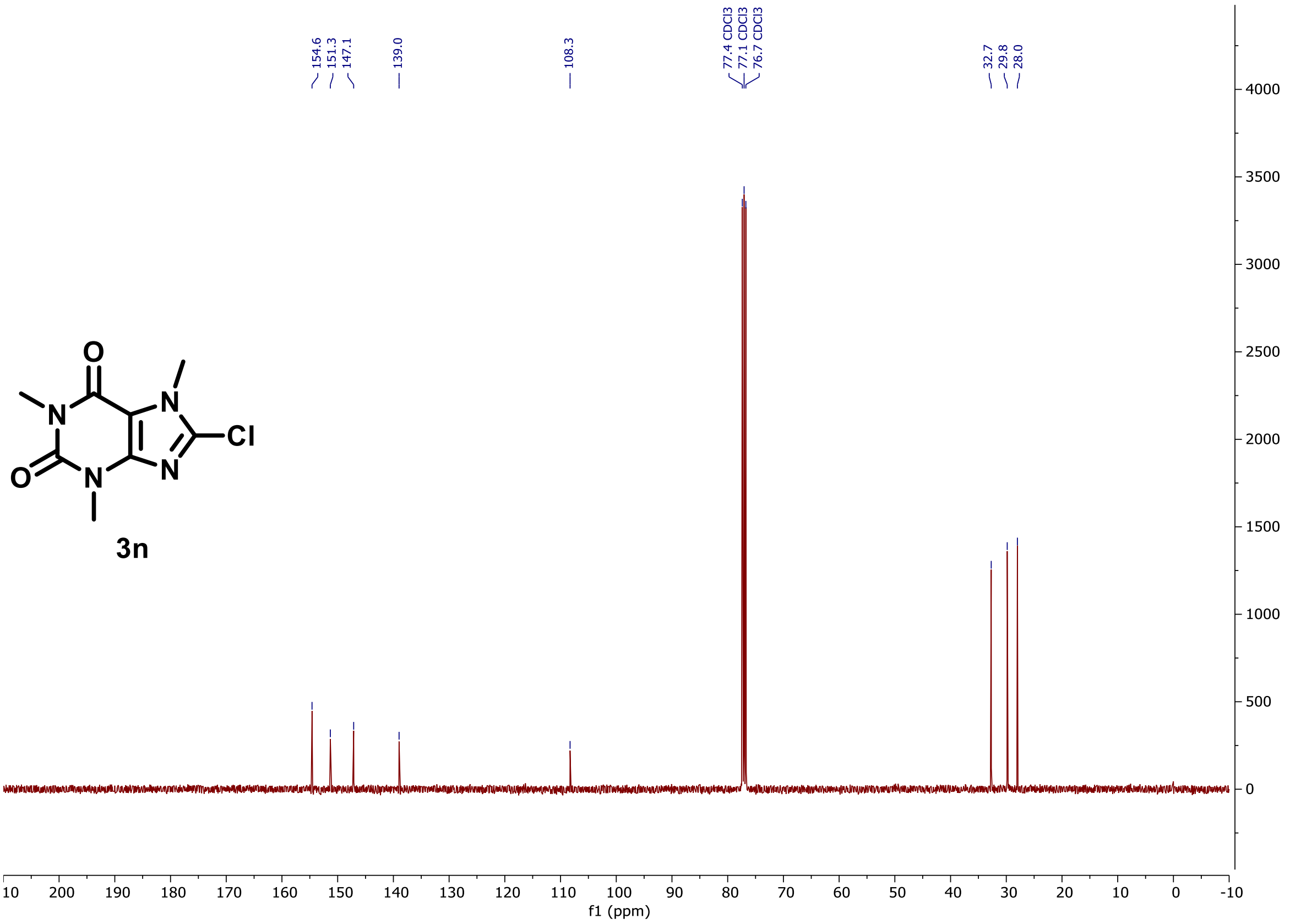

3n
154.6
151.3
147.1
139.0
108.3
77.4 CDCl3
77.1 CDCl3
76.7 CDCl3
32.7
29.8
28.0
f1 (ppm)

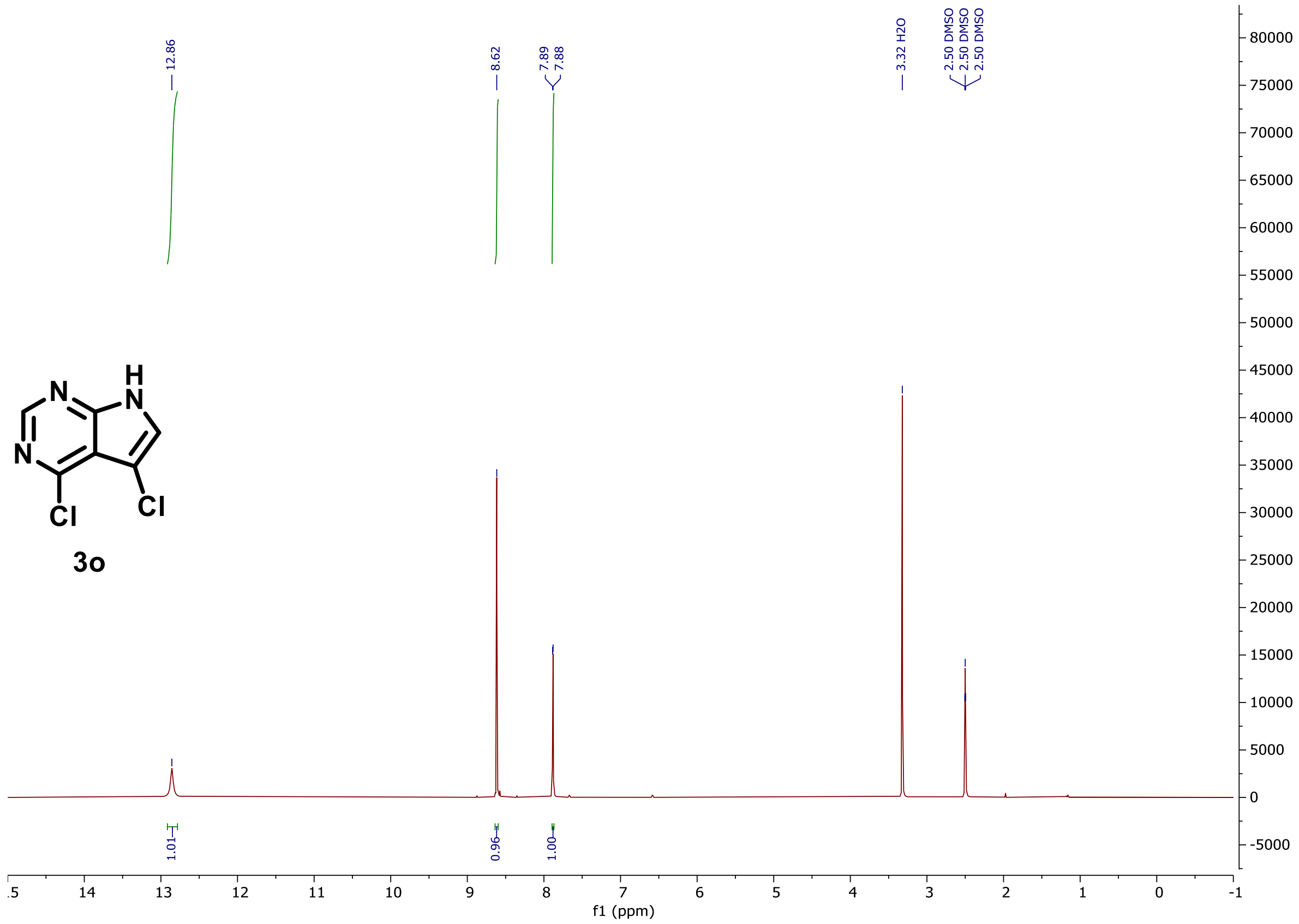
12.86
8.62
7.89
7.88
3.32 H2O
2.50 DMSO
2.50 DMSO
2.50 DMSO
1.01
0.96
1.00
3o
f1 (ppm)

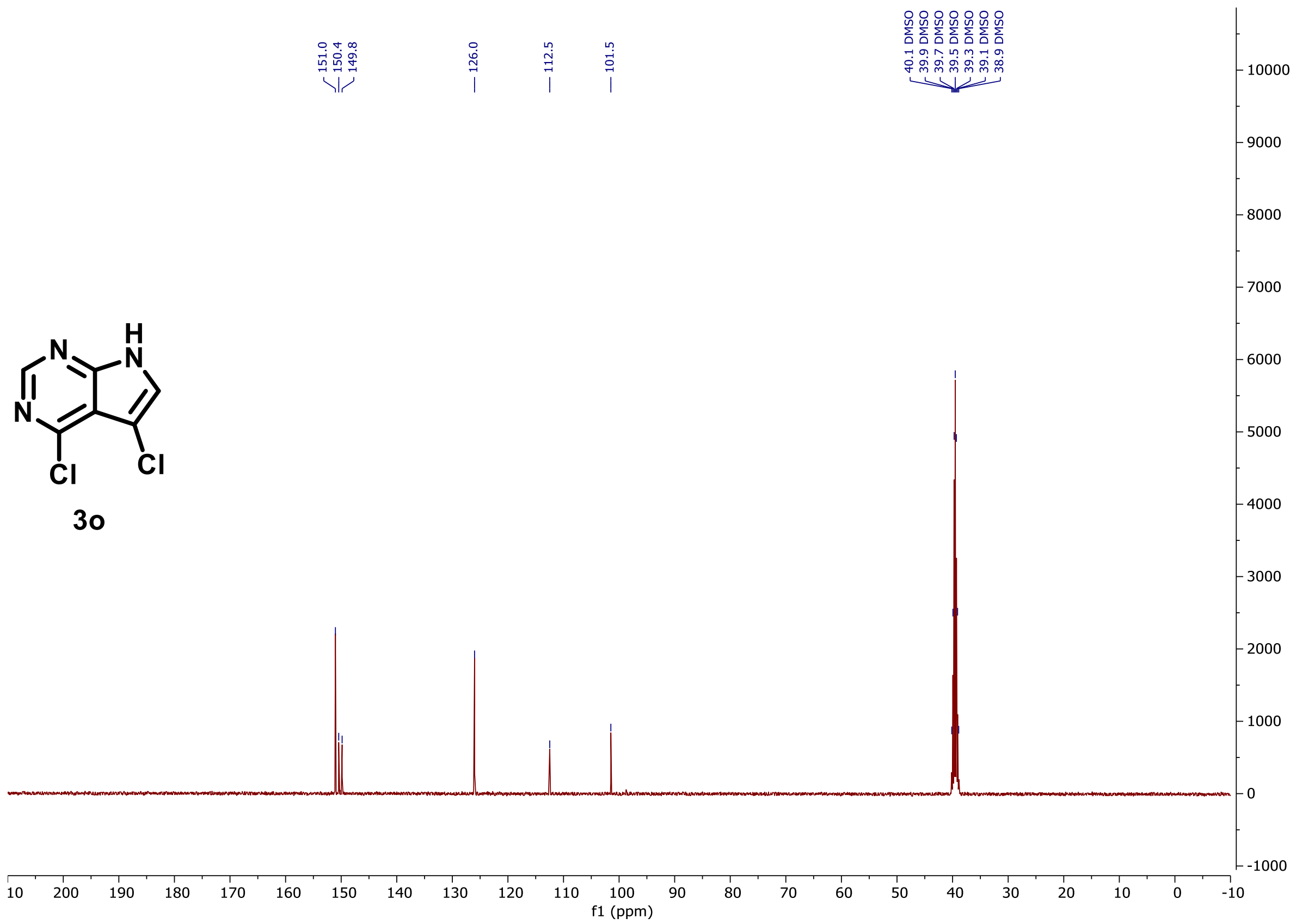
151.0
150.4
149.8
126.0
112.5
101.5
40.1 DMSO
39.9 DMSO
39.7 DMSO
39.5 DMSO
39.3 DMSO
39.1 DMSO
38.9 DMSO
3o
f1 (ppm)

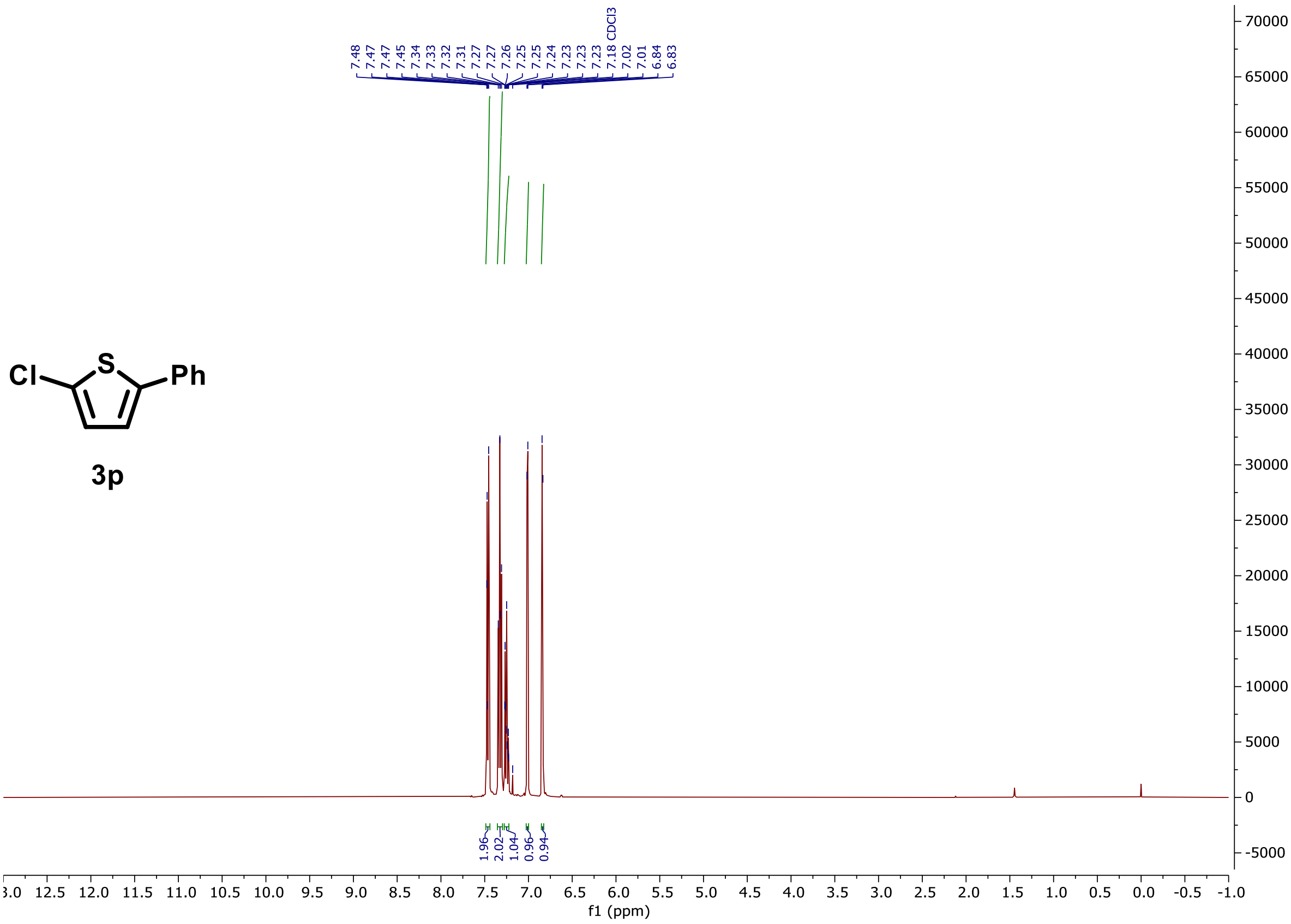
7.48 7.47 7.47 7.45 7.34 7.33 7.32 7.31 7.27 7.27 7.26 7.25 7.25 7.24 7.23 7.23 7.23 7.18 CDCl3 7.02 7.01 6.84 6.83
1.96 2.02 1.04 0.96 0.94
70000 65000 60000 55000 50000 45000 40000 35000 30000 25000 20000 15000 10000 5000 0 -5000
13.0 12.5 12.0 11.5 11.0 10.5 10.0 9.5 9.0 8.5 8.0 7.5 7.0 6.5 6.0 5.5 5.0 4.5 4.0 3.5 3.0 2.5 2.0 1.5 1.0 0.5 0.0 -0.5 -1.0
f1 (ppm)
Cl
S
Ph

3p

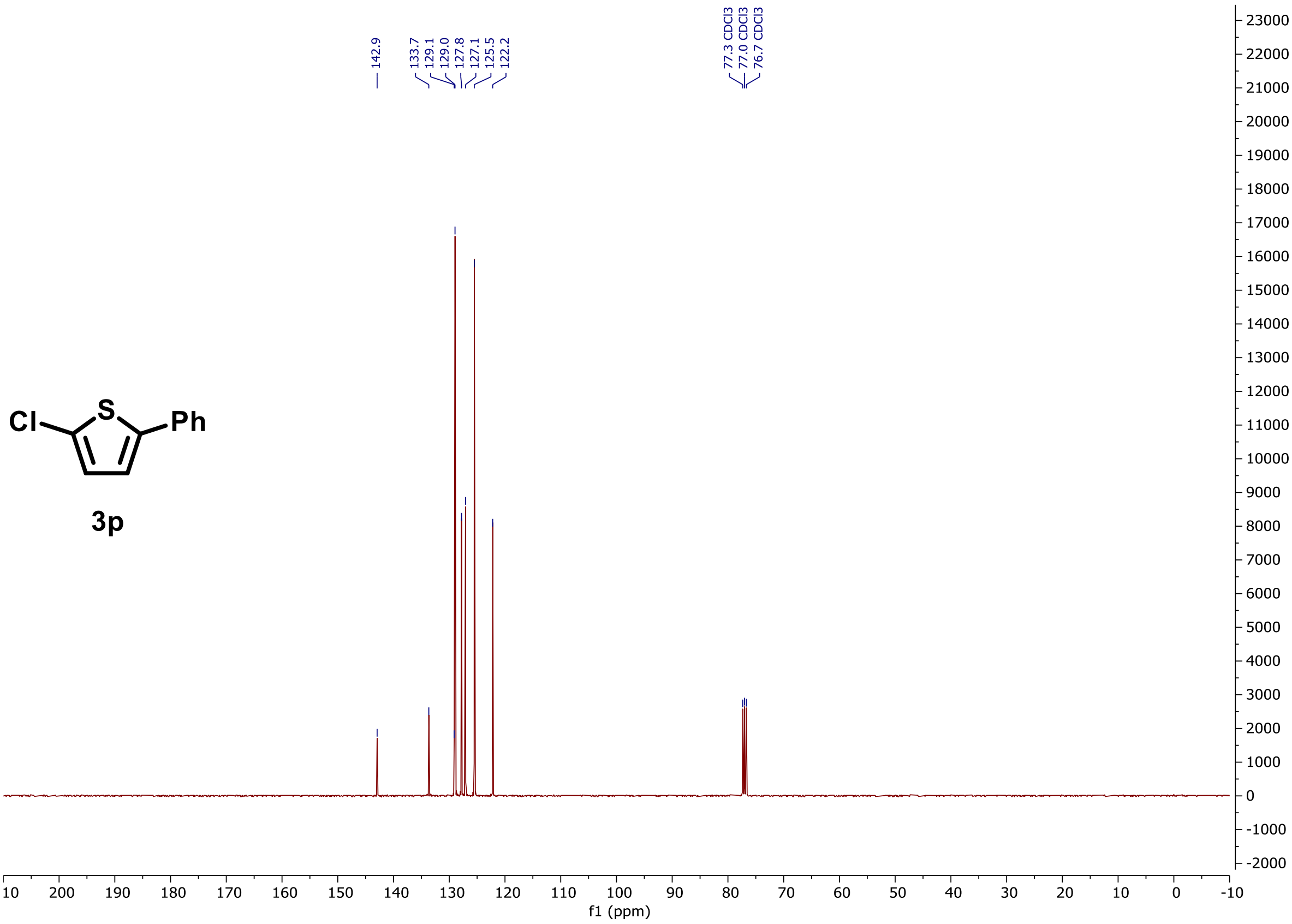
142.9
133.7 129.1 129.0 127.8 127.1 125.5 122.2
77.3 CDCl3 77.0 CDCl3 76.7 CDCl3
23000 22000 21000 20000 19000 18000 17000 16000 15000 14000 13000 12000 11000 10000 9000 8000 7000 6000 5000 4000 3000 2000 1000 0 -1000 -2000
210 200 190 180 170 160 150 140 130 120 110 100 90 80 70 60 50 40 30 20 10 0 -10
f1 (ppm)
Cl
S
Ph

3p

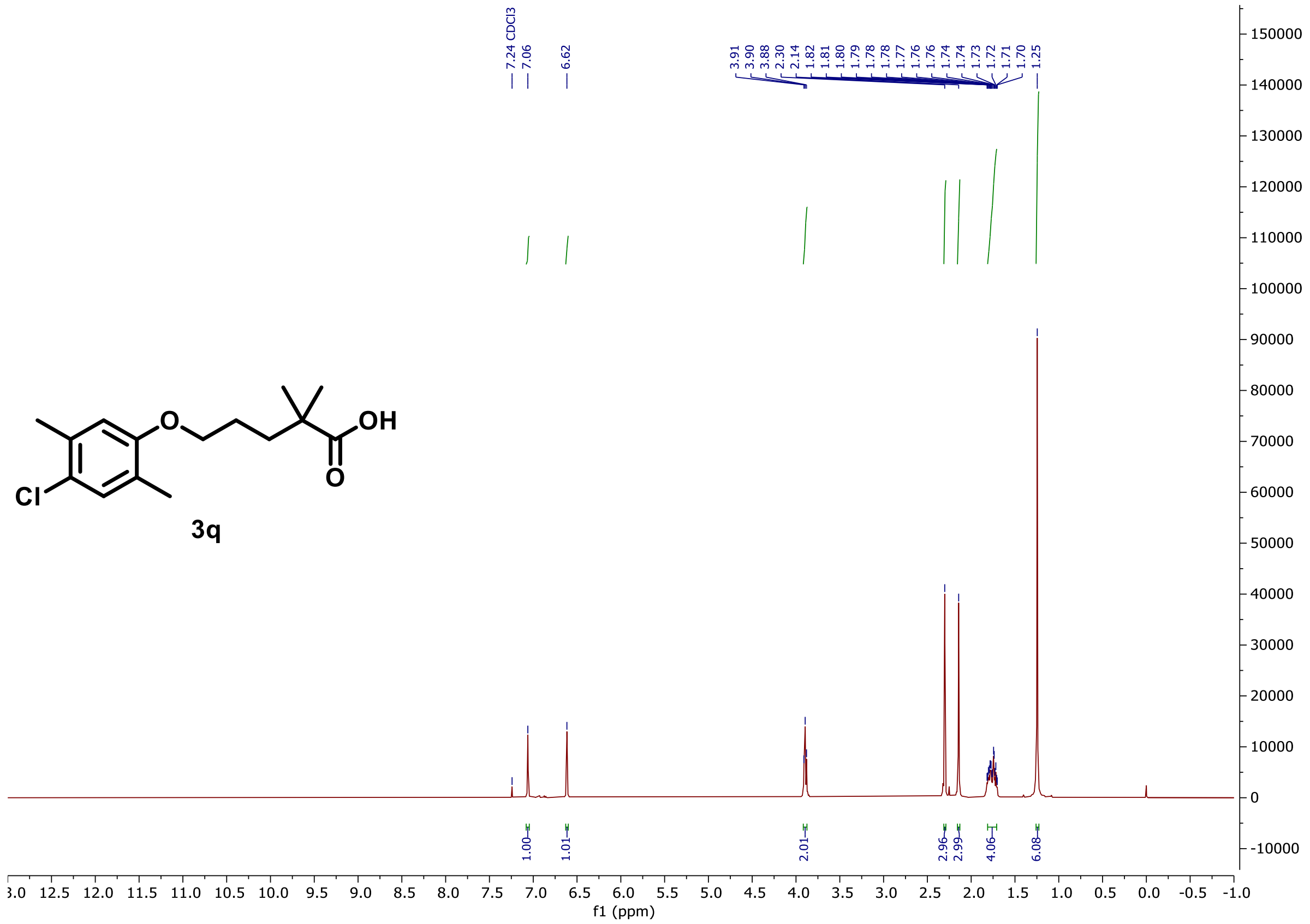
3q
OH
O
Cl
7.24 CDCl3
7.06
6.62
3.91
3.90
3.88
2.30
2.14
1.82
1.81
1.80
1.79
1.78
1.77
1.76
1.74
1.74
1.73
1.72
1.71
1.70
1.25
1.00
1.01
2.01
2.96
2.99
4.06
6.08
f1 (ppm)

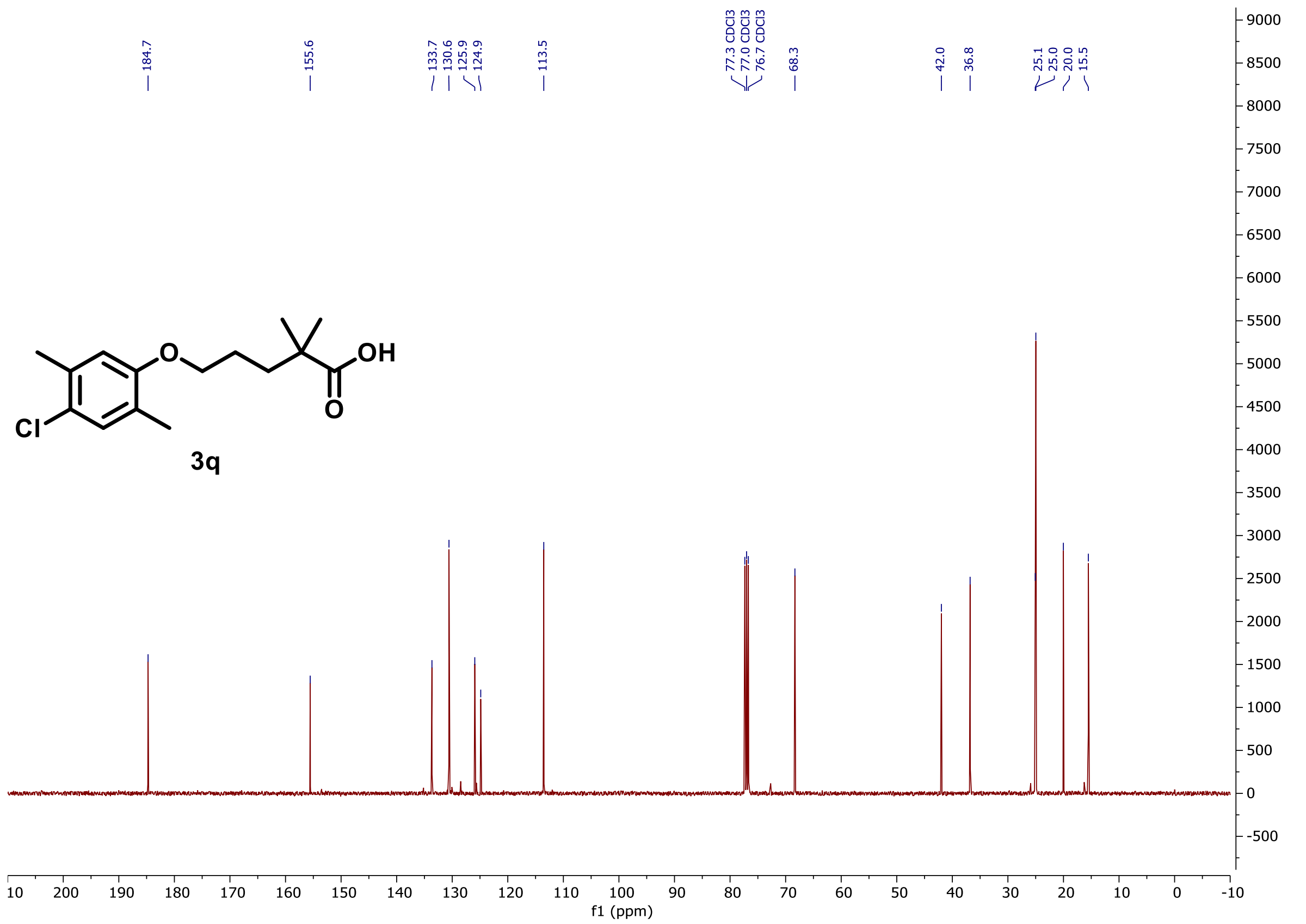
3q
OH
O
Cl
184.7
155.6
133.7
130.6
125.9
124.9
113.5
77.3 CDCl3
77.0 CDCl3
76.7 CDCl3
68.3
42.0
36.8
25.1
25.0
20.0
15.5
f1 (ppm)

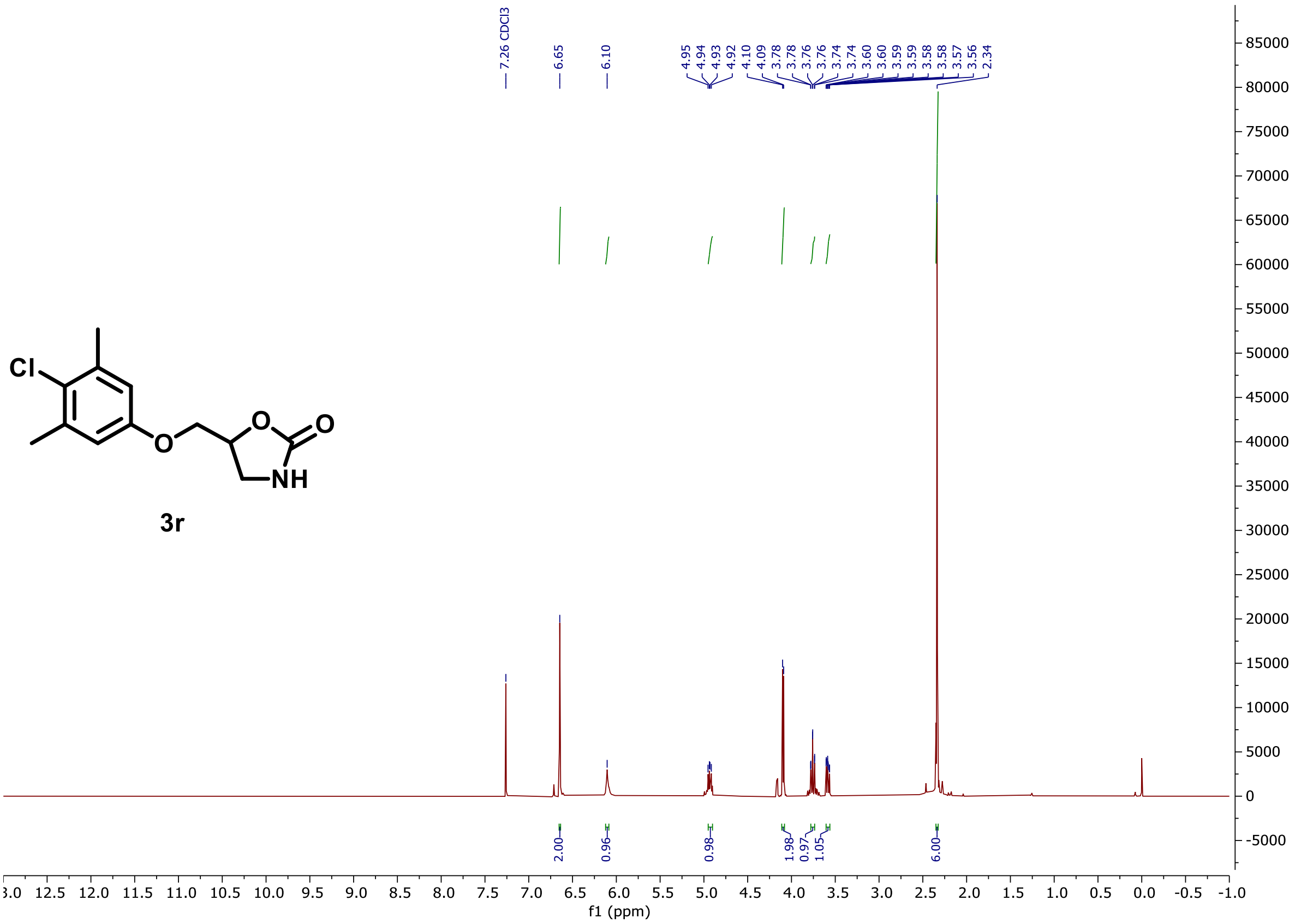
7.26 CDCl3
6.65
6.10
4.95
4.94
4.93
4.92
4.10
4.09
3.78
3.78
3.76
3.76
3.74
3.74
3.60
3.59
3.59
3.58
3.58
3.57
3.56
2.34
2.00
0.96
0.98
1.98
0.97
1.05
6.00
f1 (ppm)
Cl
O
O
O
NH

3r

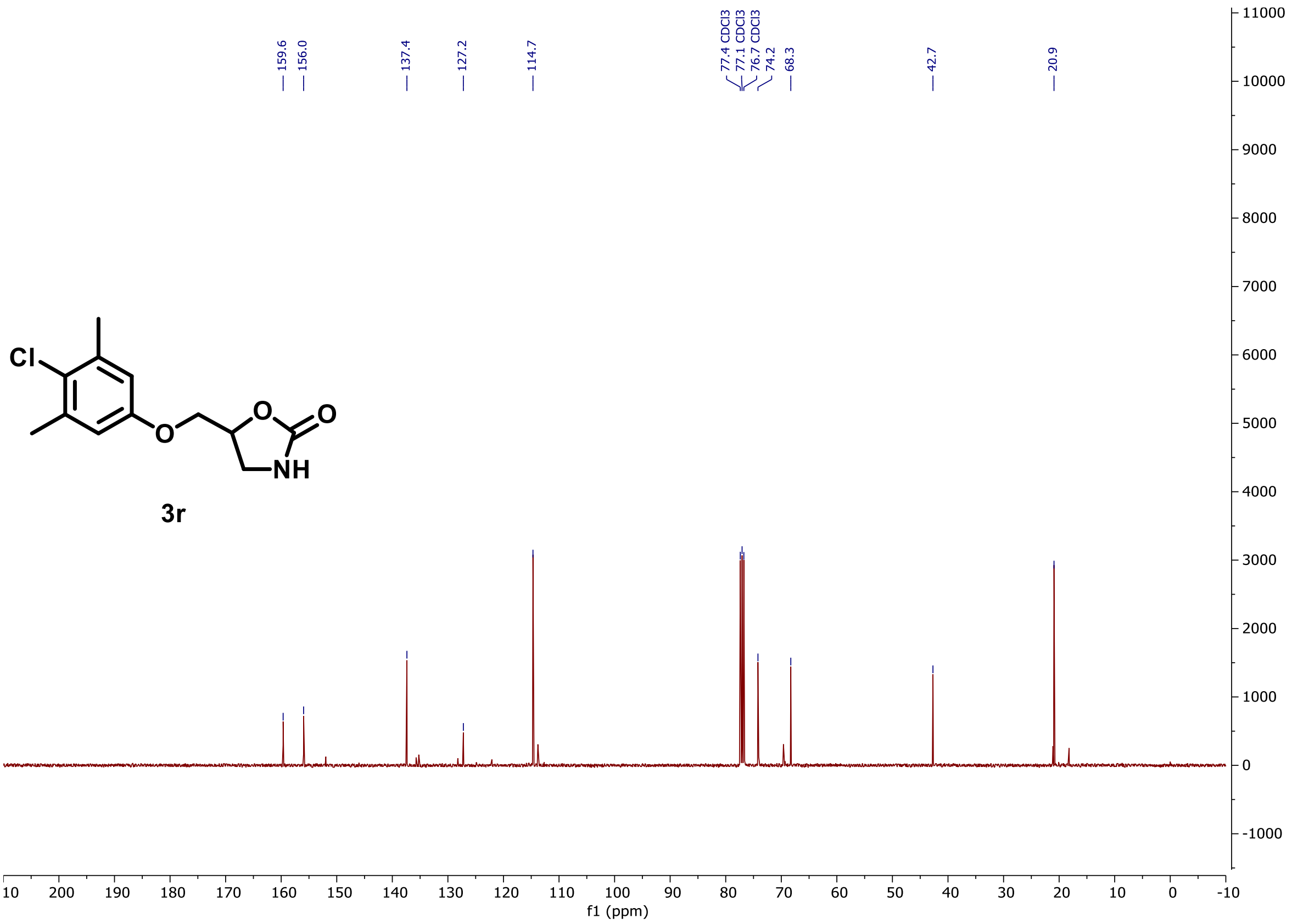
159.6
156.0
137.4
127.2
114.7
77.4 CDCl3
77.1 CDCl3
76.7 CDCl3
74.2
68.3
42.7
20.9
f1 (ppm)
Cl
O
O
O
NH

3r

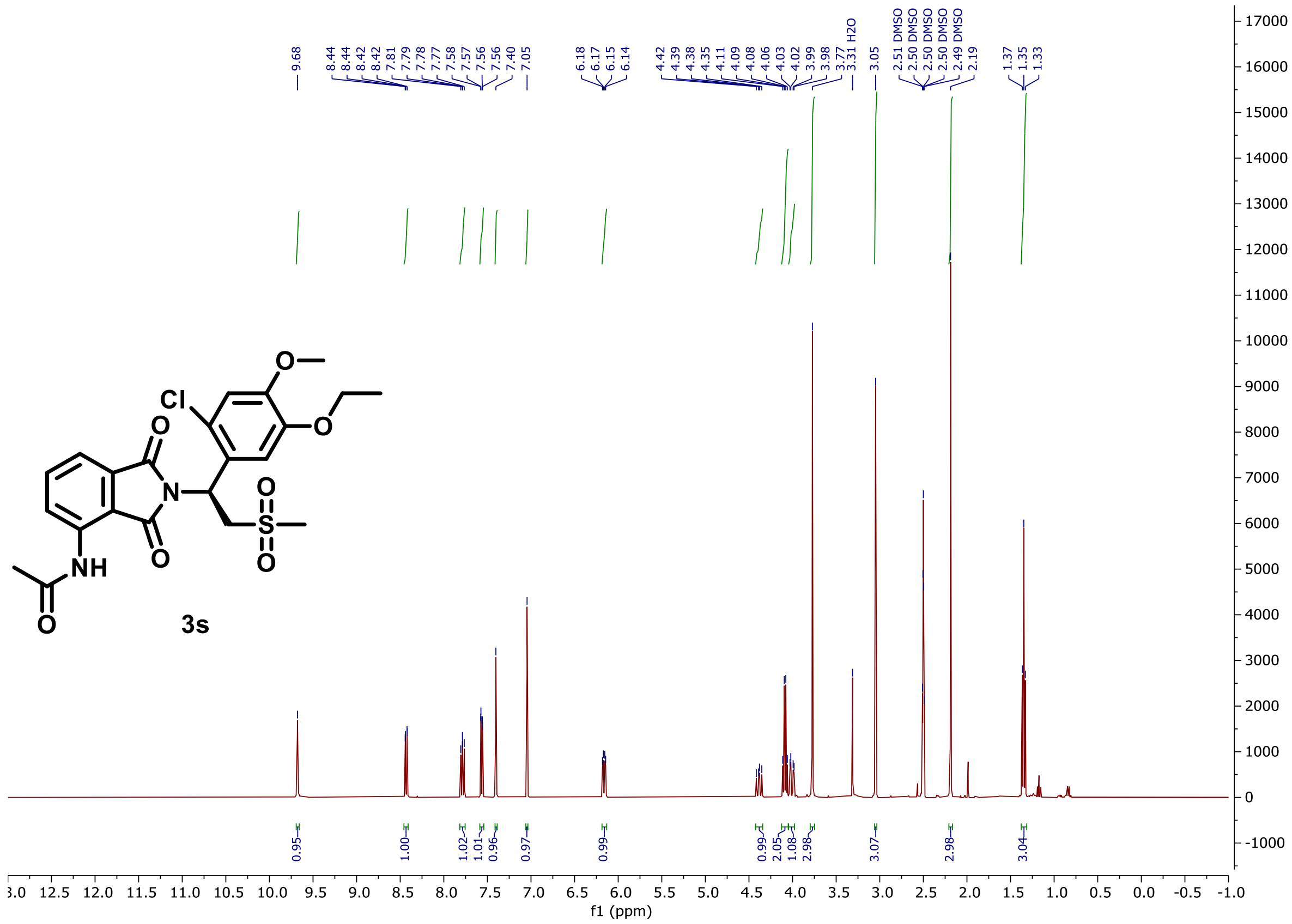
9.68
8.44 8.44 8.42 8.42 7.81 7.79 7.78 7.77 7.58 7.57 7.56 7.56 7.40 7.05
6.18 6.17 6.15 6.14
4.42 4.39 4.38 4.35 4.11 4.09 4.08 4.06 4.03 4.02 3.99 3.98 3.77
3.31 H2O
3.05
2.51 DMSO 2.50 DMSO 2.50 DMSO 2.50 DMSO 2.49 DMSO
2.19
1.37 1.35 1.33
0.95 1.00 1.02 1.01 0.96 0.97 0.99 0.99 2.05 1.08 2.98 3.07 2.98 3.04
Cl
O
N
NH
S
3s
f1 (ppm)

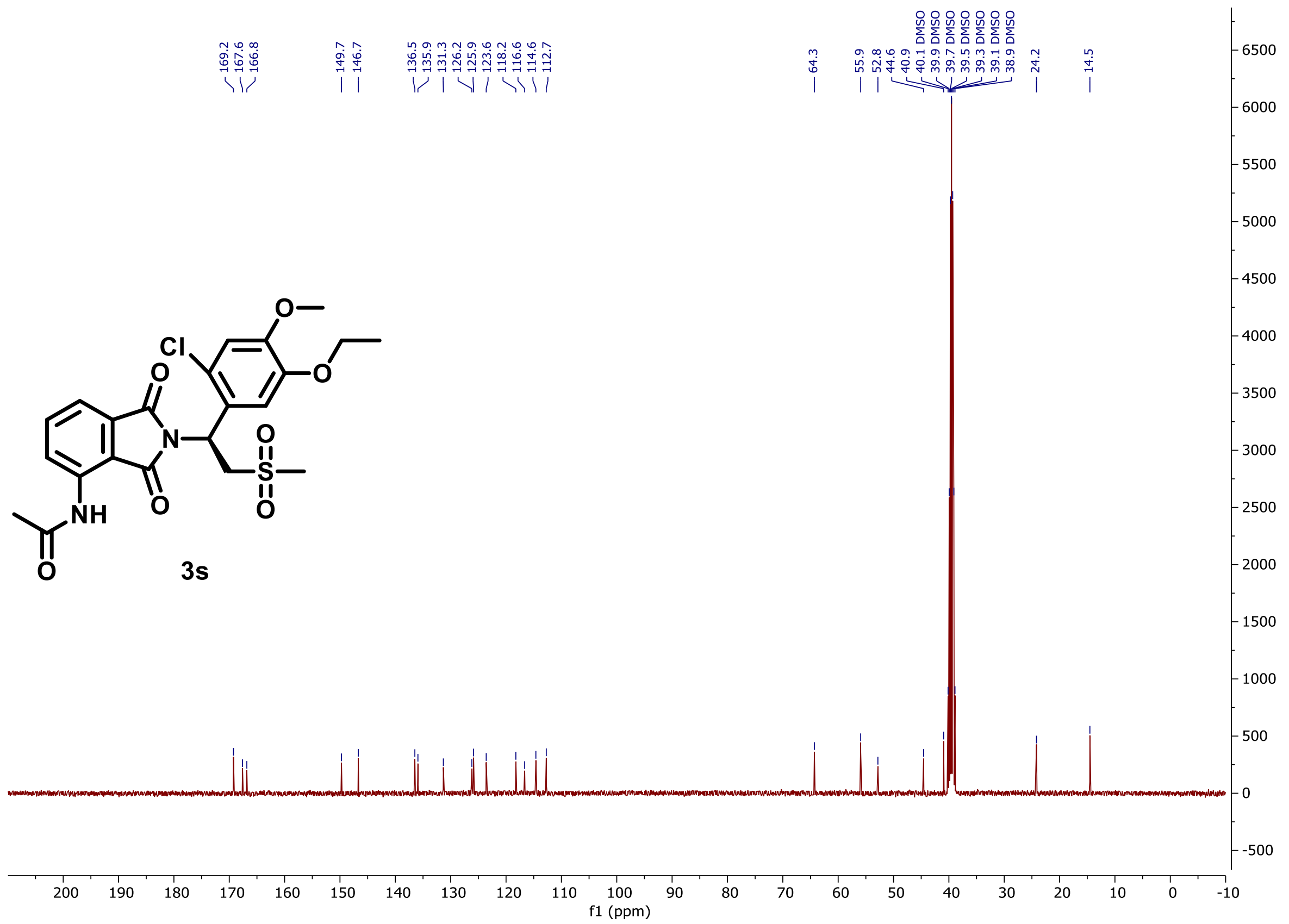
169.2 167.6 166.8
149.7 146.7
136.5 135.9 131.3 126.2 125.9 123.6 118.2 116.6 114.6 112.7
64.3
55.9 52.8 44.6 40.9
40.1 DMSO 39.9 DMSO 39.7 DMSO 39.5 DMSO 39.3 DMSO 39.1 DMSO 38.9 DMSO
24.2
14.5
Cl
O
N
NH
S
3s
f1 (ppm)

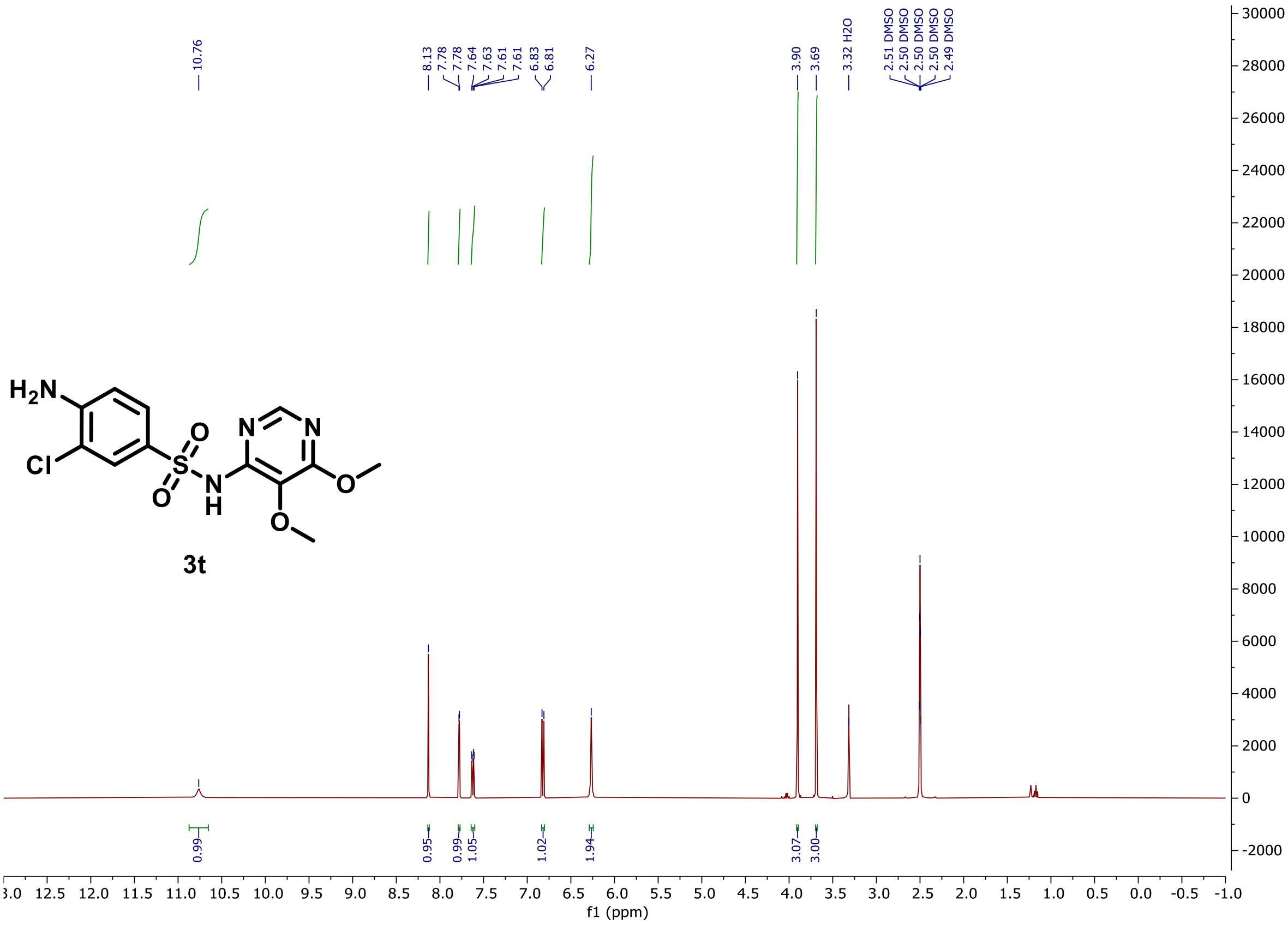

3t

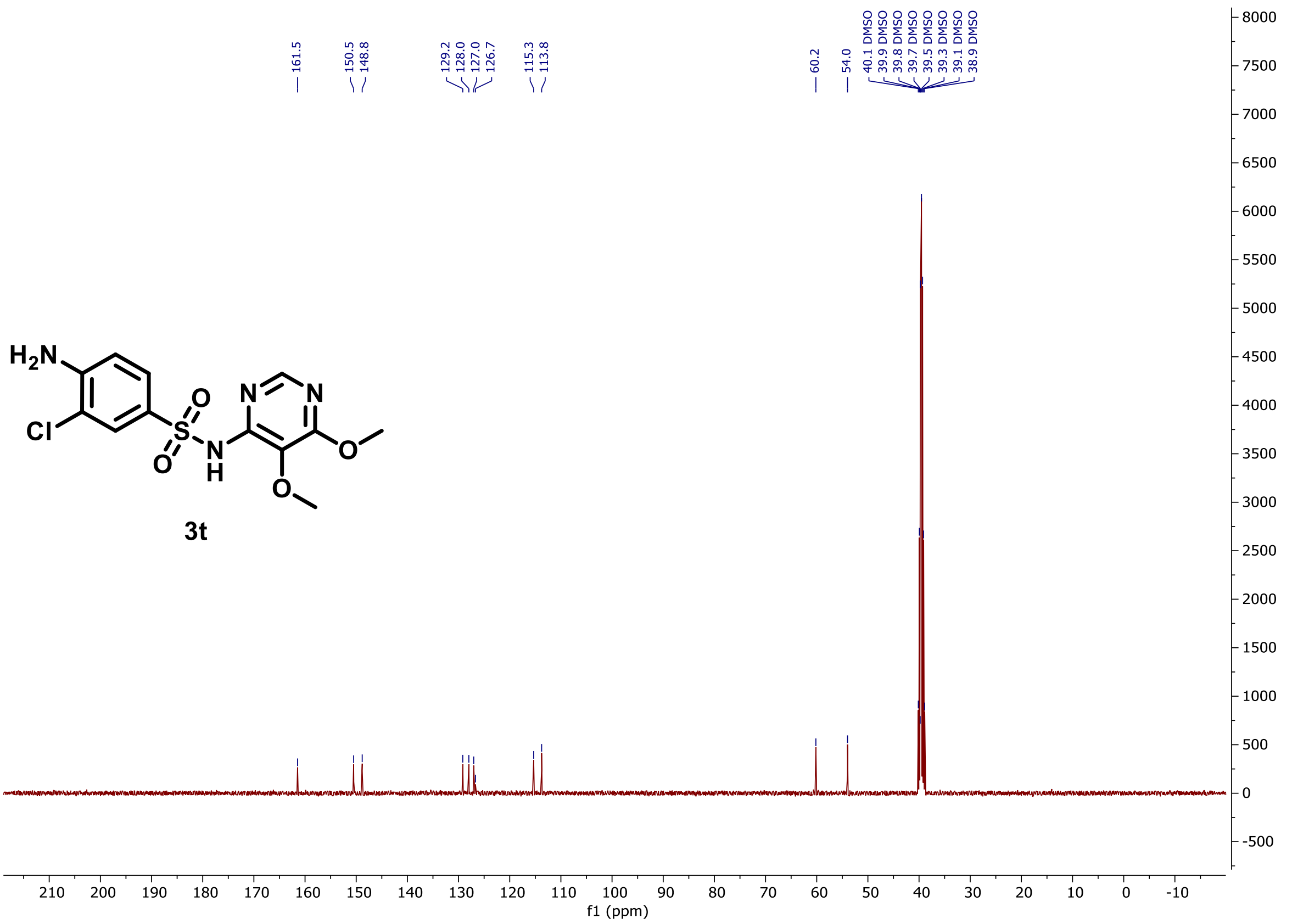

3t

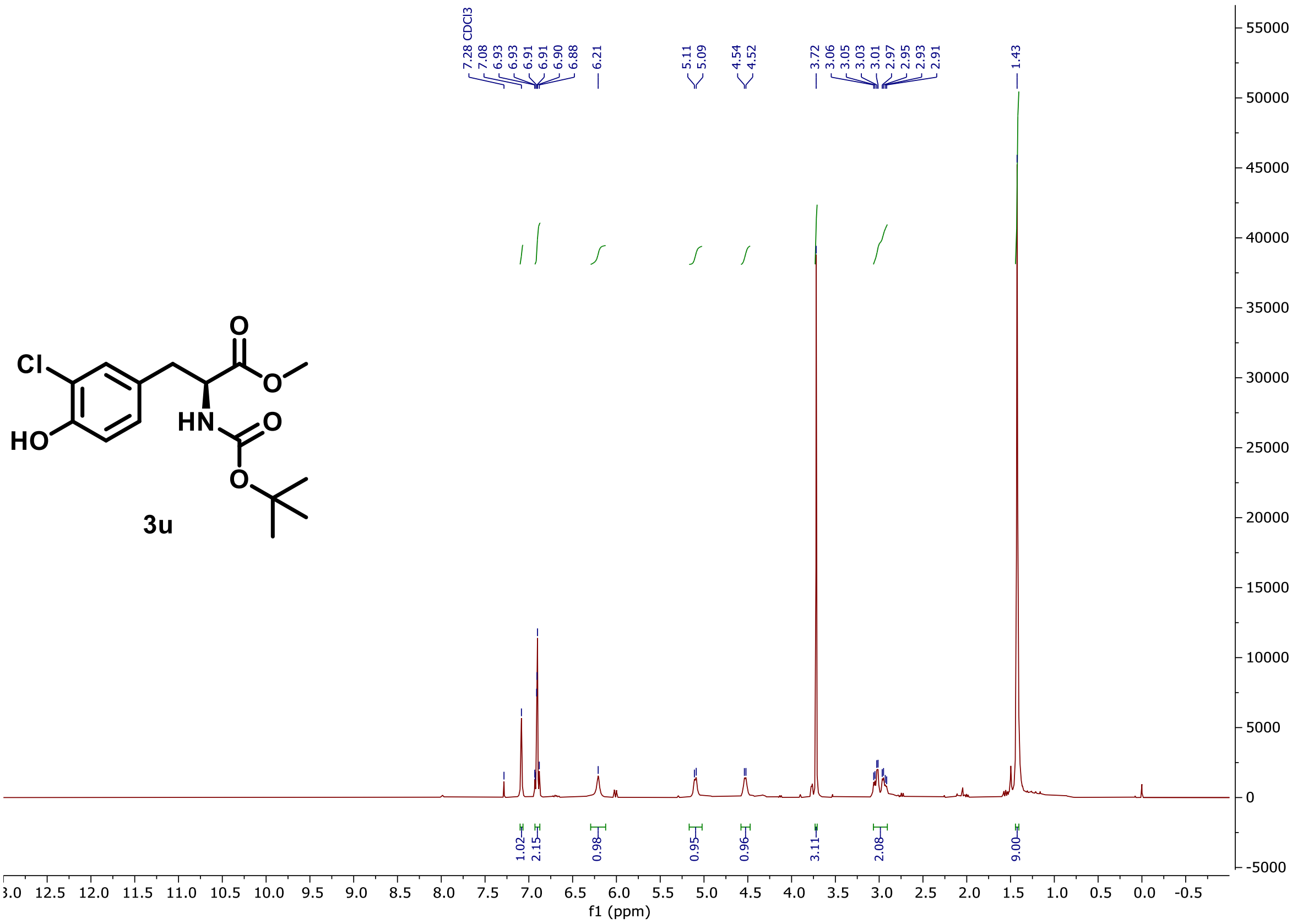
7.28 CDCl3
7.08
6.93
6.93
6.91
6.91
6.90
6.88
6.21
5.11
5.09
4.54
4.52
3.72
3.06
3.05
3.03
3.01
2.97
2.95
2.93
2.91
1.43
1.02
2.15
0.98
0.95
0.96
3.11
2.08
9.00
Cl
HO
HN
O
3u
f1 (ppm)

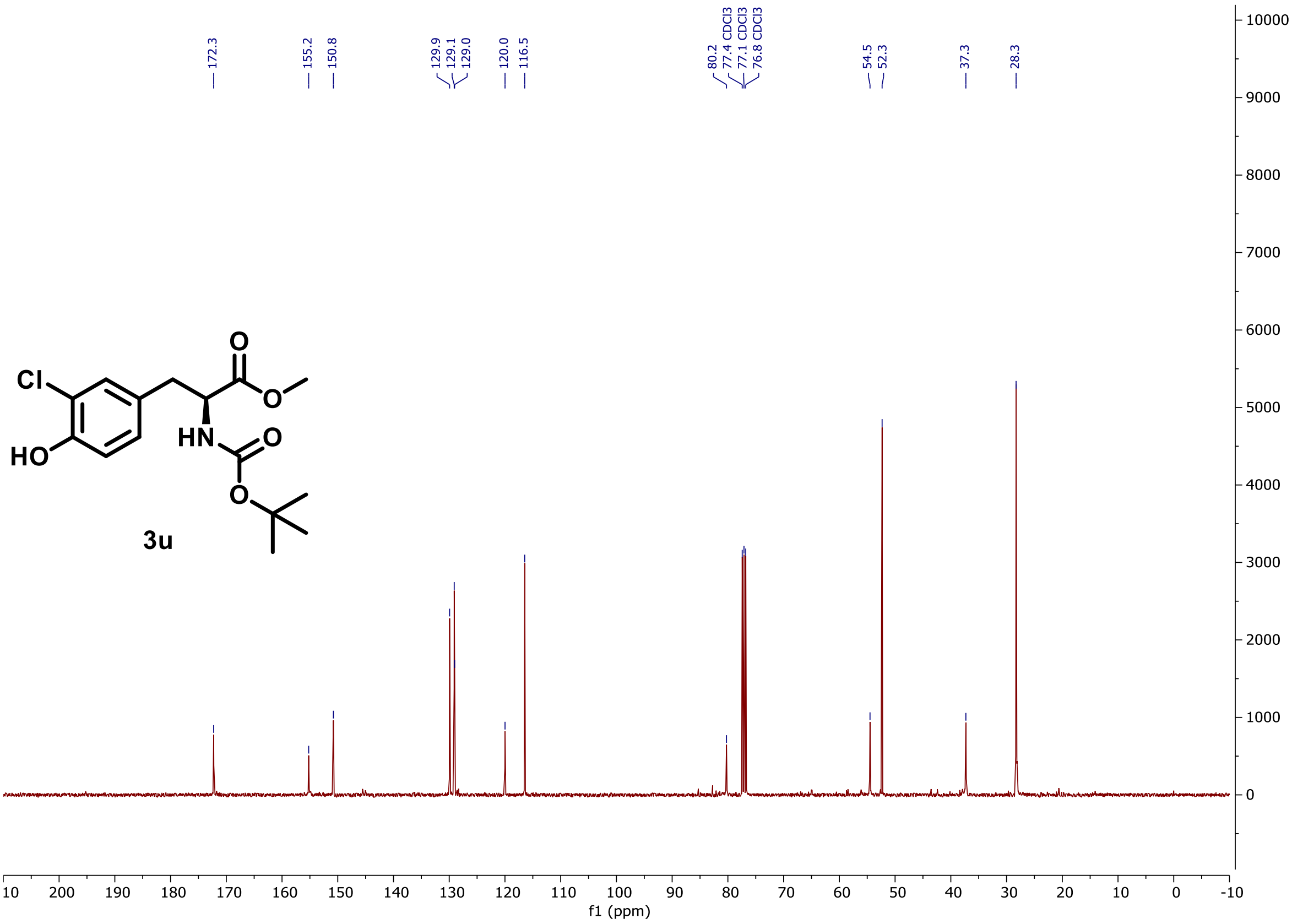
172.3
155.2
150.8
129.9
129.1
129.0
120.0
116.5
80.2
77.4 CDCl3
77.1 CDCl3
76.8 CDCl3
54.5
52.3
37.3
28.3
Cl
HO
HN
O
3u
f1 (ppm)

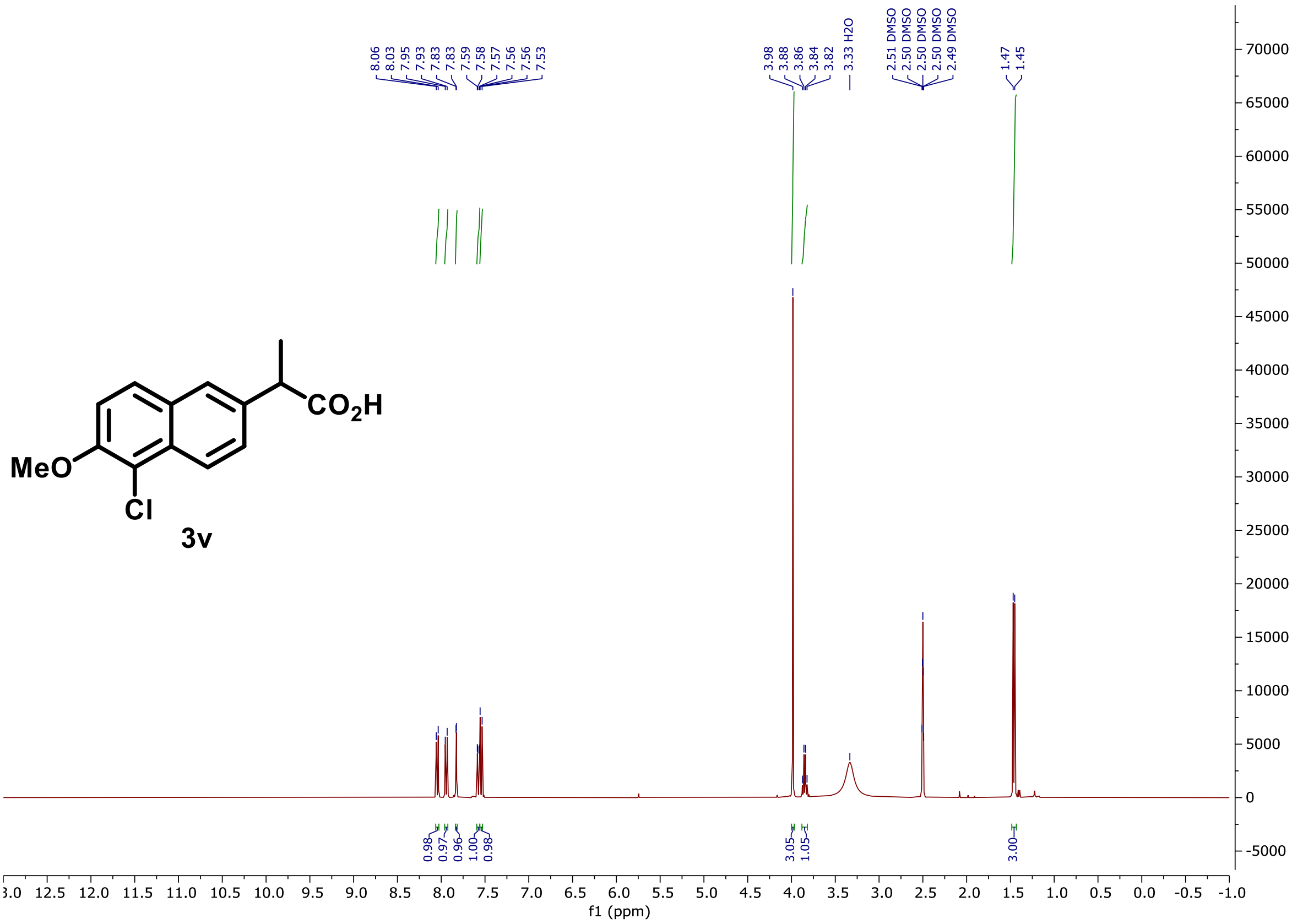

MeO
Cl
CO2H
3v
8.06 8.03 7.95 7.93 7.83 7.83 7.59 7.58 7.57 7.56 7.56 7.53
3.98 3.88 3.86 3.84 3.82
3.33 H2O
2.51 DMSO 2.50 DMSO 2.50 DMSO 2.50 DMSO 2.49 DMSO
1.47 1.45
0.98 0.97 0.96 1.00 0.98
3.05 1.05
3.00
f1 (ppm)

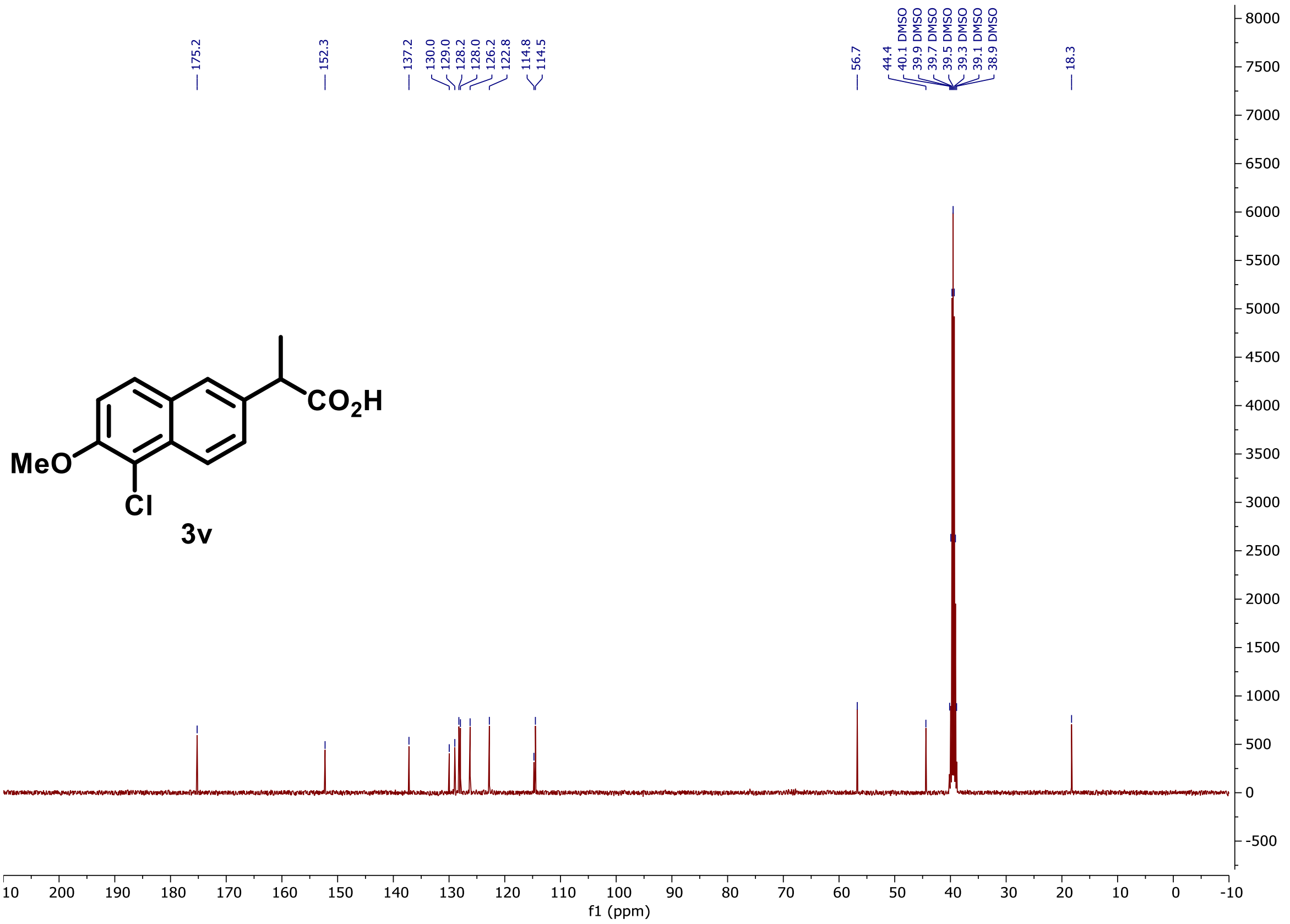

MeO
Cl
CO2H
3v
f1 (ppm)

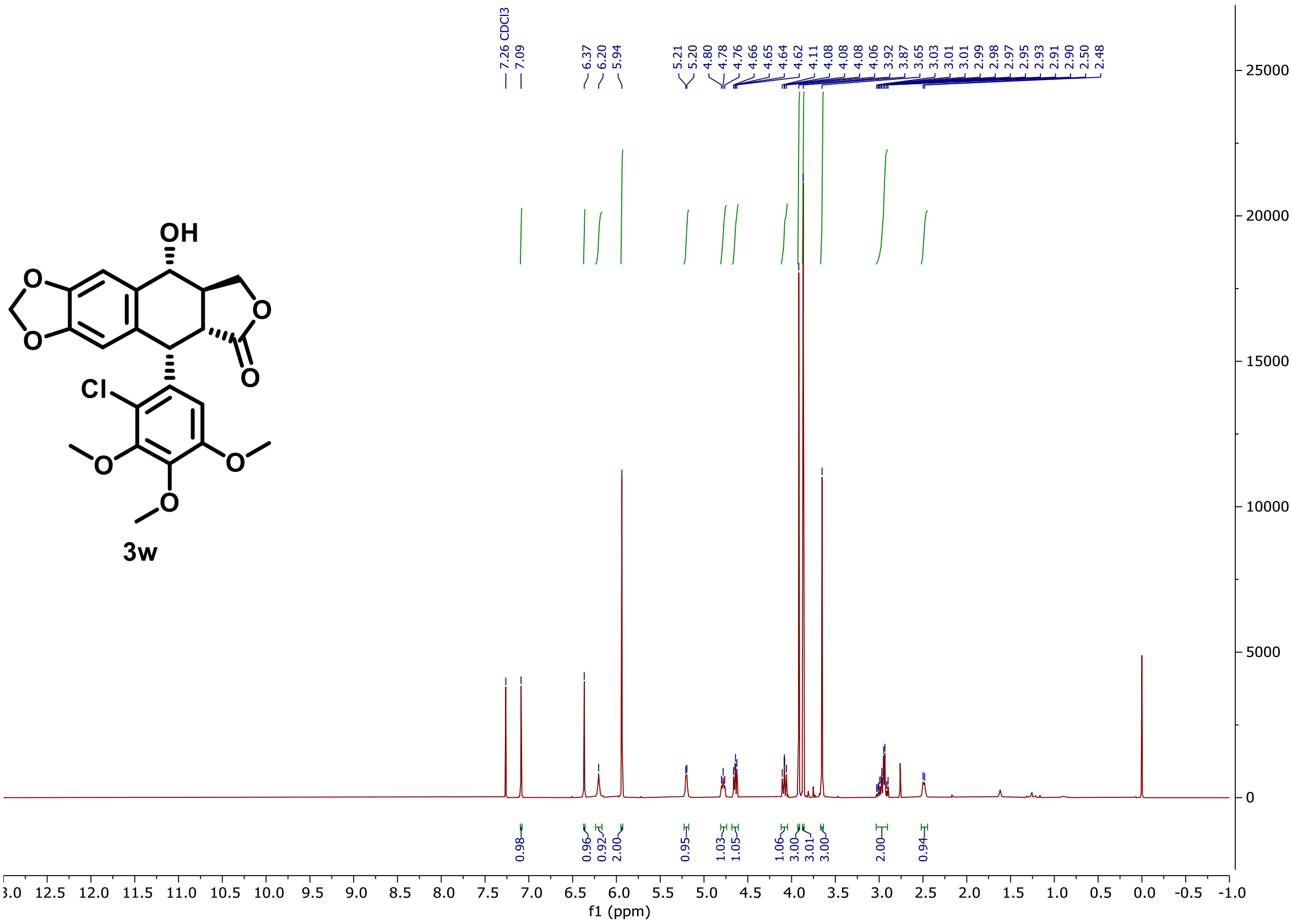
7.26 CDCl3
7.09
6.37
6.20
5.94
5.21
5.20
4.80
4.78
4.76
4.66
4.65
4.64
4.62
4.11
4.08
4.08
4.06
3.92
3.87
3.65
3.03
3.01
3.01
2.99
2.98
2.97
2.95
2.93
2.91
2.90
2.50
2.48
OH
O
O
O
O
Cl
O
O
O
3w
0.98
0.96
0.92
2.00
0.95
1.03
1.05
1.06
3.00
3.01
3.00
2.00
0.94
f1 (ppm)

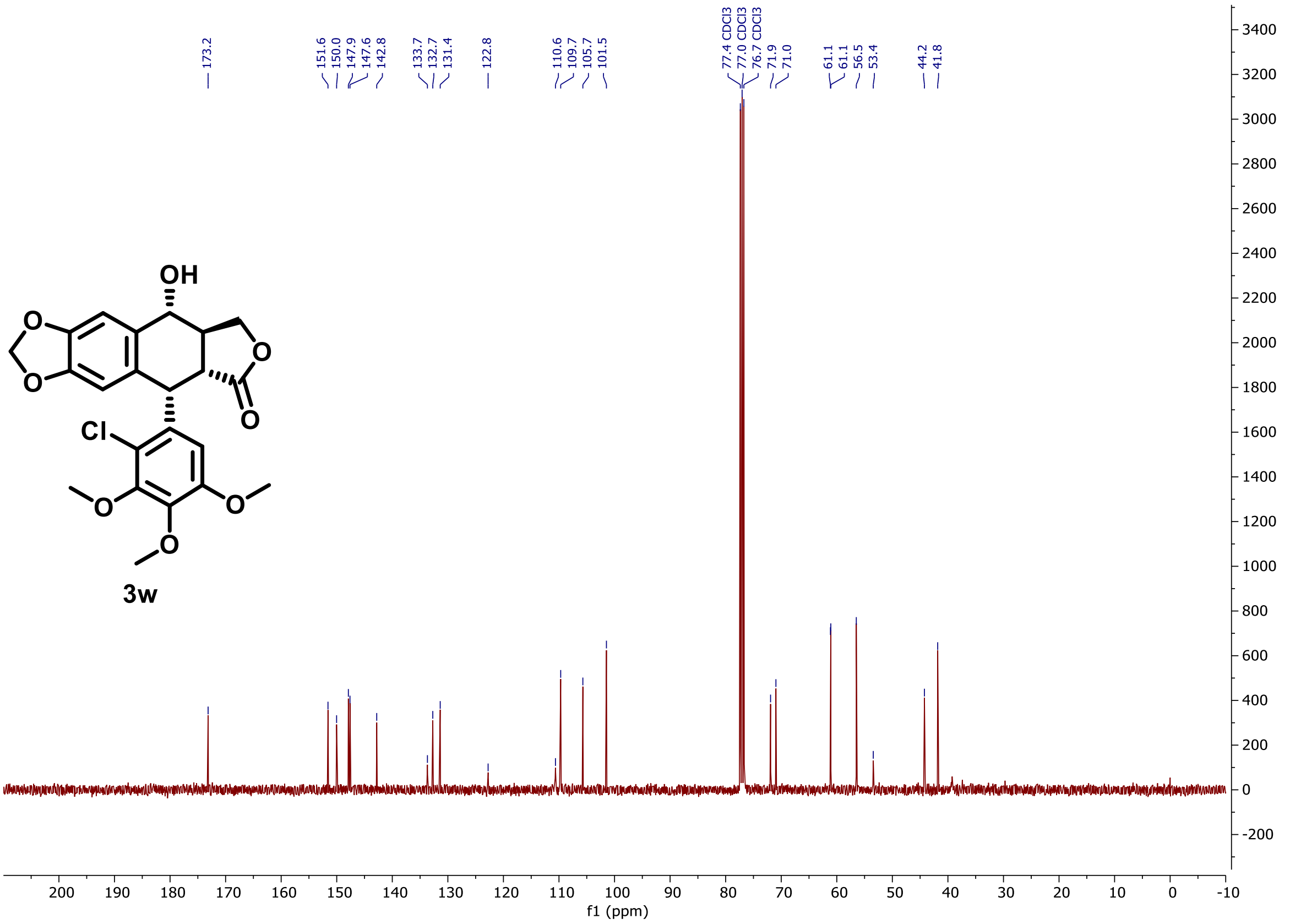
173.2
151.6
150.0
147.9
147.6
142.8
133.7
132.7
131.4
122.8
110.6
109.7
105.7
101.5
77.4 CDCl3
77.0 CDCl3
76.7 CDCl3
71.9
71.0
61.1
61.1
56.5
53.4
44.2
41.8
OH
O
O
O
O
Cl
O
O
O
3w
f1 (ppm)
